# Longitudinal evaluation of interventions on antimicrobial use and antimicrobial resistance on broiler farms in West Java, Indonesia

**DOI:** 10.1016/j.psj.2025.106010

**Published:** 2025-10-27

**Authors:** Rianna Anwar Sani, Annisa Rachmawati, Sunandar Sunandar, Gian Pertela, Elvina J. Jahja, Imron Suandy, CORNERSTONE group, Jan van den Broek, Sjaak J. de Wit, Jaap A. Wagenaar, David C. Speksnijder, Francisca C. Velkers

**Affiliations:** a Division of Infectious Diseases and Immunology, Faculty of Veterinary Medicine, Utrecht University, 3584 CL Utrecht, The Netherlands; b Center for Indonesian Veterinary Analytical Studies (CIVAS), Bogor 16130, Indonesia; c Animal Health Department, PT Medion Farma Jaya, Bandung 40552, Indonesia; d Directorate General of Livestock and Animal Health Services, Ministry of Agriculture, Indonesia; e Department of Population Health Sciences, Faculty of Veterinary Medicine, Utrecht University, 3584 CL Utrecht, The Netherlands; f Royal GD, Arnsbergstraat 7, 7418 EZ Deventer, The Netherlands; g WHO Collaborating Center for Reference and Research on Campylobacter and Antimicrobial Resistance from a One-Health Perspective/WOAH Reference Laboratory for Campylobacteriosis, 3584 CL Utrecht, The Netherlands

**Keywords:** Antimicrobial use, Antimicrobial resistance, Intervention study, Indonesia, Poultry

## Abstract

To address the growing public health concern of antimicrobial resistance (AMR), this study evaluated the impact of a combination of targeted interventions on antimicrobial use (AMU) and AMR in commensal *Escherichia coli* within small- and medium-scale broiler farms in West Java, Indonesia. This longitudinal study was conducted from 2019 to 2023 and included data from 98 production cycles (across 19 farms) pre-intervention and 55 production cycles (across 14 farms) post-intervention. AMU was assessed using count-based treatment frequency per standardized 30-day cycle. Changes in AMU and AMR were analyzed using a hierarchical Bayesian negative-binomial regression and Bayesian Gaussian linear model, respectively, and included potential risk factors for AMU including mortality rates, biosecurity levels and seasonality. Fluoroquinolones were the most frequently used antimicrobials pre- and post-intervention. The observed total AMU (aggregated across all classes) decreased from 0.4 to a treatment frequency of 0.3, corresponding to a reduction from an average of 11 to 9 treatment days per cycle. Although the model did not indicate a significant effect of the intervention on overall AMU**,** a significant decline of 92.6 % in colistin use was observed, most likely attributable to a national ban on colistin use implemented in the study period. The proportion of *E. coli* isolates exhibiting non-wild-type phenotypes—defined as those with minimum inhibitory concentration (MIC) values above the epidemiological cutoff (ECOFF), indicating a deviation from the wild-type population—did not decline post-intervention across all 14 tested antimicrobials. The AMR model did not demonstrate a clear effect of the included risk factors but suggested a potential increase in resistance associated with higher levels of AMU. This study provides a comprehensive longitudinal assessment of AMU and AMR in small- and medium-scale broiler farms in West Java. The outcomes provide valuable baseline AMU and AMR levels and underscore the potential, and complexity, of targeted interventions to reduce AMU and mitigate AMR, and the need for further investigation into the effectiveness of AMU reduction strategies.

## Introduction

Indonesia, the fourth largest country in the world, has a substantial poultry industry. The Indonesian broiler sector counted 3.1 billion birds in 2023 (Livestock and Animal Health Services, 2024), and average yearly consumption of poultry meat in Indonesia had increased from 5.7 kg per capita in 2019 to 7.4 kg in 2024 (Livestock and Animal Health Services, 2024). With the growing demand for animal protein, the industry is expected to expand even further in the coming years. Considering the large and increasing size of the broiler sector, addressing potential public health challenges related to broiler production is of high relevance (Chapot et al., 2023).

Antimicrobial use (**AMU**) in livestock has been identified as one of the key contributors to antimicrobial resistance (**AMR**) in bacteria, which impacts animal, human and environmental health (Holmes et al., 2016; Magnusson et al., 2021; Malijan et al., 2022; Malik et al., 2023). In Indonesia’s broiler sector, approximately 80 % of antimicrobials are used preventively, with enrofloxacin being most used (Suandy, 2019). Enrofloxacin is classified as a highest priority critically important antimicrobial (**HPCIA**) by the World Health Organization (**WHO**) (WHO, 2024). To address AMR, the Indonesian government’s National Action Plan aimed to reduce preventive AMU in broilers from the estimated 80 % in 2020 to 50 % by 2024 (Ministry of Health of the Republic of Indonesia, 2022).

The Indonesian broiler sector is concentrated mainly on Java, with West Java contributing to 45 % of national production (Suandy, 2019). Although large commercial companies hold over 80 % market share, a large number of poultry is still raised by small-scale (housing 5,001–50,000 broilers) and medium-scale (housing 50,001–1,000,000 broilers) enterprises (Pakage et al., 2018; Suandy, 2019; Nugroho, 2020; Chapot et al., 2023). These classifications of small- and medium-scale farms are based on standards defined by the Indonesian Ministry of Agriculture (Ministry of Agriculture of the Republic of Indonesia, 2020). According to the Food and Agriculture Organization of the United Nations (**FAO**), these farms fall under sector 2 and 3 categories when considering the level of biosecurity (FAO, 2008). Management practices on these farms vary widely, partly due to distinct business models. These business models create differing incentives for biosecurity and disease prevention, as responsibilities and financial risks vary across farmer types.

To address the risk of AMR, there is a need for targeted interventions stimulating antimicrobial stewardship (**AMS**). In this study we use the WHO definition of AMS, being a coherent set of actions which promote the responsible use of antimicrobials (Smith et al., 2019). Acknowledging the diversity in business types, interventions cannot be “one size fits all” but must be specifically tailored to each farm. Previous studies in other countries on interventions to promote prudent AMU in livestock have primarily focused on improving biosecurity and husbandry practices (Speksnijder et al., 2015a; Roskam et al., 2019; Phu et al., 2020). However, to the best of the authors’ knowledge no intervention study targeting AMU on small- and medium-scale broiler farms in Indonesia has been published.

The study was part of the larger CORNERSTONE project (“Containment of antimicrobial resistance: towards a sustainable poultry production chain in Indonesia”) (Wagenaar, 2019). The project aimed to investigate which interventions would be best to promote prudent AMU by improving on-farm management practices on small- and medium-scale broiler farms in West Java, Indonesia.

The study evaluated the effect of interventions on AMU and AMR, using commensal *Escherichia coli* (***E. coli****)* as indicator bacteria, by comparing pre- and post-intervention data with a focus on changes in overall AMU and the relative use of (highest priority) critically important antimicrobials.

## Materials and methods

**Fig. 1:**
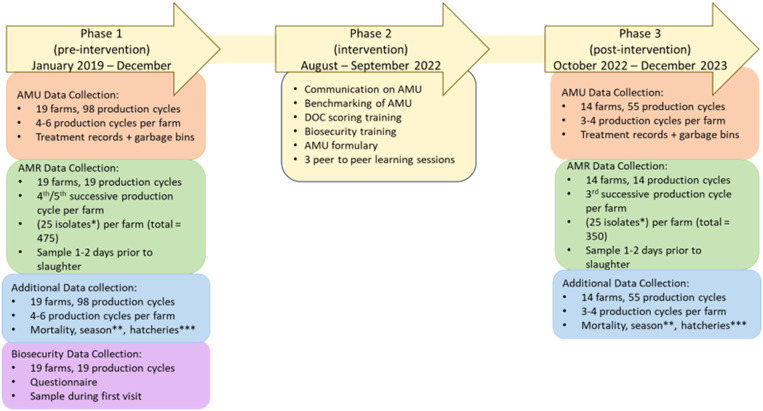
Schematic overview of the study design divided into 3 phases: Phase 1 (pre-intervention), Phase 2 (intervention) and Phase 3 (post-intervention). Data collection is categorized into antimicrobial use (AMU) data, antimicrobial resistance (AMR) data, additional data, and biosecurity data. For each data category, different quantities were used (as visible in the Figure). *commensal *E. coli* isolates were isolated and analysed **season was recorded as dry- (May – September) or rainy-season (October – April) ***hatcheries refers to the number of different hatcheries the Day-Old Chicks (DOC) that arrive on the farm originated from.

This study used a longitudinal intervention design and focused on the impact of interventions on AMU and AMR. The study was divided into a pre-intervention phase (Phase 1), an intervention phase (Phase 2) and a post-intervention phase (Phase 3) (Fig. 1) and was conducted between 2019 and 2023. Interventions that were considered to potentially reduce AMU were implemented between August and September 2022 (Phase 2).

### Farm and sample selection

Farms were selected using convenience sampling from Medion's client database, an Indonesian veterinary pharmaceutical company, with selection criteria to only include small- and medium-scale farms, either contract-based or independent, located in West Java.

Initially, 25 farms were recruited; however, during Phase 1, 6 small-scale farms withdrew due to farming cessation or changes in ownership or management. During Phase 3, an additional 5 small-scale farms withdrew for similar reasons. This resulted in a final sample of 19 (16 small-scale and 3 medium-scale) farms in Phase 1, and 14 (11 small-scale and 3 medium-scale) farms in Phase 3. Farms in this study included traditional open-housed, semi-closed housed, and close-housed farms. Open-housed systems consist of a form of stilts of wood or bamboo which are very dependent on outside temperature and humidity conditions (Setianto et al., 2021). Semi-closed systems are modified open houses that allow for limited control of temperature and/or humidity. In closed-house systems, temperature and humidity are controlled by climate control equipment (Setianto et al., 2021).

### Interventions

The interventions consisted of several complementary strategies (see Box. 1), developed based on scientific literature and discussions within the research consortium (Speksnijder et al., 2015a; Roskam et al., 2019; Phu et al., 2020). Extension workers from the Center for Indonesian Veterinary Analytical Studies (**CIVAS**) visited farms regularly to collect AMU data, assess flock health, and discuss prudent AMU practices with farmers. After the baseline phase, farms were benchmarked on AMU based on previously developed AMU monitoring tools (Anwar Sani et al., 2023). They received tailored dashboards showing performance, AMU, and resistance data, together with recommendations. Farmers took part in biosecurity and day-old chick (**DOC**) quality training, both followed by individual discussions to translate lessons into farm-specific actions. Peer-to-peer learning sessions (3 in total) combined expert input with farmer-led discussion on good broiler management, colibacillosis control, and practical ways to reduce AMU. Finally, an antimicrobial formulary was introduced as a practical guide to support more targeted, stepwise treatment decisions.

**Box 1:** Explanation of the interventions that were conducted to stimulate prudent antimicrobial use (AMU).

Intervention	Description
Communication on AMU	Extension workers from the Centre for Indonesian Veterinary Analytical Studies (CIVAS) visited farms 3 times per production cycle to assist with AMU data collection, assess flock condition, and encourage prudent AMU.
Benchmarking of AMU	After Phase 1, farms were benchmarked based on AMU, identifying high users (upper quartile). All farms received a tailored dashboard with broiler performance, AMU, and antimicrobial resistance (AMR) data, alongside recommendations for improvement.
Biosecurity Training	Farmers received practical tools to enhance biosecurity, followed by individual discussions on feasible measures for their farms.
Day-Old Chick (DOC) Scoring Training	Farmers were trained to assess DOC quality using a scoring form, allowing them to adjust management practices and promote responsible AMU.
Peer-to-Peer Learning	3 sessions were held, combining expert lectures (30-60 min) with farmer-led discussions to share experiences and insights. The topics of the sessions were:
	Good Broiler Management Practices (featuring a representative from broiler breeding company)
	Controlling Colibacillosis and what is prudent AMU (featuring 2 veterinary academics)
	Practical on-farm strategies to reduce AMU (featuring an international veterinary poultry consultant)
Antimicrobial Formulary	A practical guide was provided to support farmers in selecting targeted antimicrobials based on observed clinical signs, including first, second, and third choice options for treatment.

### Overview of data collection procedures

Data collection occurred during Phase 1 (between 2019 and 2021) and during Phase 3 (between 2022 and 2023) across multiple successive production cycles per phase (4 to 6 production cycles per farm in Phase 1, 3-4 production cycles in Phase 3) to assess AMU patterns and the susceptibility of commensal *E. coli* isolates per farm house to 14 antimicrobials (12 different antimicrobial classes) (Anwar Sani et al., 2024). The average length of a production cycle on our study farms was 30 days.

Extension workers from CIVAS visited the farms 3 times per production cycle to collect data and instructed the farmers on data collection procedures. Farmers submitted weekly photos or copies of daily records, which included the number of chicks at the start of the cycle, mortality, number of broilers sent to slaughter, and slaughter weight.

Commensal *E. coli* was used as indicator organism for susceptibility testing (Anwar Sani et al., 2024). In Phase 1, sampling was carried out 1-2 days before slaughter of the 4^th^ successive production cycle (on farm 4 and 15 this was done during the 5^th^) from which the usage data were collected (Fig. 1). In Phase 3, AMR sampling was conducted 1–2 days before slaughter during the 3^rd^ successive production cycle. The extension worker used the boot swab method for fecal droppings sample collection, which consisted of walking at least 100 steps in a zigzag pattern through the whole broiler house while wearing sterilized boot covers (Cover Shoes PP Sterile, Antonides B.V., Erp, the Netherlands), commonly used for litter sampling for bacteria in broiler flocks. Every 1/5^th^ of the house, the litter sample attached to the boot cover was collected in a plastic bag using a sterile wooden tongue depressor. This resulted in 1 pooled sample per farm, with a required minimum weight of 25 grams of feces combined with litter.

### Data collection

***Antimicrobial Use (AMU).*** AMU data were collected during multiple production cycles in Phase 1 and Phase 3. AMU data was collected from 98 production cycles during Phase 1 and 55 cycles during Phase 3, as described previously (Anwar Sani et al., 2023). In brief, AMU data were recorded through daily treatment records and by analyzing empty packages and bottles in the drug bins, documenting the date and age of the broilers at application, the (brand) name of the veterinary medical product (**VMP**), purpose of use, the amount of the products used, and the route of application.

Quality check of the AMU data was performed manually by 3 independent researchers checking the input of the used products and their content. The active substance(s) of the VMPs that had been applied by the farmers were obtained through the Index for Veterinary Medicines Indonesia and cross-checked by a veterinarian from CIVAS.

A count-based indicator was most suitable to quantify AMU in our study setting (Anwar Sani et al., 2023). Every single day of treatment with an antimicrobial product was counted as 1 treatment day. The sum of treatment days over 1 production cycle was divided by the days at risk (30-day production cycle), resulting in a Treatment Frequency (**TF_count-based_**):

$$TFcount-based=\;\frac{number\;of\;treatment\;days\;of\;specific\;antimicrobial\;agent}{number\;of\;days\;at\;risk\;}$$

If a VMP containing 2 antimicrobial classes was used, it was counted as 2 treatment days.

To assess the variety and level of sub- or overdosing on top of the primarily used count-based indicator, the average used daily dose (**UDD**) was also calculated across all production cycles during Phase 1 (n = 98) and during Phase 3 (n = 55). The UDD was calculated as:

$$UDD\;\left(mg/kg\right)=\frac{amount\;of\;active\;substance\;administered\;per\;treatment\;\left(mg\right)}{N\;treated*standardized\;bodyweight\;at\;day\;of\;treatment\;\left(kg\right)}$$

In addition to the quantity used per antimicrobial class, the purpose of use (either preventive or therapeutic treatment) was also recorded by the farmers.

***Antimicrobial Resistance (AMR).*** A detailed protocol of the AMR data collection has been described previously (Anwar Sani et al., 2024). In short, the susceptibility (Minimum Inhibitory Concentration (**MIC**)) profiles of 25 *E. coli* isolates per farm were assessed by the microbroth dilution method using Sensititre EUVSEC plates with 14 antimicrobials (Thermo Fisher Scientific, East Grinstead, United Kingdom) (Supplementary File 1 (**S1**)). The complete laboratory protocol can be found in the supplementary files (**S2)**. The prevalence of non-wild type (**NWT**) phenotype was determined using the European Committee on Antimicrobial Susceptibility Testing (**EUCAST**) epidemiological cut-off (**ECOFF**) values (European Committee on Antimicrobial Susceptibility Testing, 2020). Clinical breakpoints (**CBPs**) are available for certain combinations of pathogenic bacteria, specific hosts, and antimicrobials, but are not established for commensal bacteria. As a result, some studies conducted in Southeast Asia on poultry apply human CBPs to commensal *E. coli* to assess AMR. (Gibson et al., 2020; Inthavong et al., 2022). To facilitate comparison with these regional studies, human CBPs from the Clinical and Laboratory Standards Institute (**CLSI**) were included (Clinical Laboratory Standards Institute, 2024). For tigecycline, only the ECOFF sourced from EUCAST was used, due to unavailability of a CBP for tigecycline. Both ECOFF and CBP data were obtained from their respective sources in September 2024.

***Additional data relevant to AMU and AMR.*** Previous studies indicated associations in AMU with various internal or external risk factors on broiler farms (Dhaka et al., 2023; Mallioris et al., 2023).

Of these potential risk factors, we collected data on first week mortality (Joosten et al., 2019), biosecurity levels (Sarini et al., 2013; Mallioris et al., 2023), the number of DOC suppliers (hatcheries) per production cycle and seasonality (Umair et al., 2021).

First week mortality, i.e. the percentage of chickens culled or deceased within their first week of life, can be an indicator of DOC quality and early-stage management (Joosten et al., 2019). It is expected to be at maximum 1.0 % according to the Cobb management guide (Cobb, 2021) but mostly ranges between 0.6 and 2.1 % in European broilers (Forseth et al., 2024). Poor DOC quality has been identified as a primary reason for AMU during the first week (Hibbard et al., 2023) . Broilers in 1 house originating from multiple hatcheries can also be associated with an increase in first week mortality.

Previous studies showed associations with AMU and the level of biosecurity (Sarini et al., 2013; Mallioris et al., 2023). This was only assessed once, at the beginning of Phase 1, through a detailed questionnaire comprising 115 questions across 10 categories (Table 1). The questionnaire was developed and tailor made using multiple sources, including checklists from the ViParc project (Carrique-Mas and Rushton, 2017), FAO (Coyne et al., 2020b), expert input from the broiler sector through consortium discussions, and a framework based on the Theory of Planned Behavior (Francis et al., 2004), with full details provided in the Supplementary File 3 (S3).

**Table 1:** Overview of the included categories from the biosecurity questionnaire performed in Phase 1 on 19 broiler farms in this study with the total number of questions per category and, between brackets, the number of questions with a binary outcome.

Category	Number of questions (of which yes/no questions)^1^
External biosecurity	13 (12)
Employees and equipment	8 (8)
Movements and purchases	7 (7)
Feed and water management	14 (14)
Internal biosecurity & disease management	26 (19)
Management of veterinary drugs	6 (2)

1 The proportion (score) per category which was used for the analysis was calculated by dividing the number of questions answered with yes by the total number of binary (yes/no) questions (between brackets) per category. The full questionnaire, included discarded categories and questions, can be found in Supplementary File 3 (S3).

Biosecurity level was quantified by assigning a score to each category based on the proportion of binary (yes/no) questions answered with “yes”, with a higher score indicating best practices. Although this approach assigns equal weight to all questions, we acknowledge that in practice, their contributions to biosecurity may vary depending on the context. The categories of stocking density, feed composition, therapy failure, and disinfection were excluded, as the corresponding questions or quality of the responses made these unsuitable for including it in our data analysis. For example, although disinfection practices are relevant, the questions focused on product types rather than disinfection procedures, limiting its use for answering our research question.

Seasonality was included as an external environmental factor, as farmers reported greater temperature and humidity fluctuations during the rainy season (October to April), which they mentioned as a reason for increased AMU (Umair et al., 2021).

### Data analysis

All collected data were first entered in Microsoft Excel 365 (Microsoft Corp., Redmond, USA) and then reformatted for analysis in R version 4.3.1 (R Core Team, 2023). To evaluate the effect of our interventions on AMU and AMR, we used 2 separate statistical models. As this was the first longitudinal intervention study targeting AMU on small- and medium-scale broiler farms in Indonesia, no formal sample size calculation was conducted; instead, a pragmatic approach was taken, including farms that voluntarily agreed to participate. The complete R codes for these analyses are provided in Supplementary File 4 (S4). Details of each model’s variables and their inclusion are provided in Table 2.

**Table 2:** Overview of the characteristics and the variables included in the statistical models to analyse the effect of interventions between Phase 1 and 3 for antimicrobial use (AMU) and antimicrobial resistance (AMR).

Variable	Category	AMU model	AMR model
Model types		Hierarchical Bayesian negative-binomial regression sparse model	Bayesian Gaussian linear model for AMR
Included number production cycles		142 (Phase 1: 95, Phase 3: 47)	99 (Phase 1: 57, Phase 3: 42)
N_resistant	Dependent		Average number (out of 14) of antimicrobials to which the 25 *E. coli* isolates per farm showed an NWT phenotype based on ECOFF^4^
AB_count^1^	Dependent	Overall AMU model: Sum of treatment days of all antimicrobial classes combined	
		AMU model per specific class: Sum of treatment days of each specific antimicrobial class^1^	
Phase	Independent (categorical (cat))	Indicating whether the observations were done during Phase 1 (pre-intervention) or Phase 3 (post-intervention)	
N_broilers	Independent (continuous (cont))	Number of broilers in the study house at start of production cycle	
n_broilers	Independent (cont)		Average number of broilers present in the study house across the 3 production cycles prior to AMR sampling
Mortality	Independent (cont)	Percentage of chickens culled or deceased in the first week per production cycle	
Firstweekmort	Independent (cont)		Average percentage of first week mortality, calculated across 3 production cycles prior to AMR sampling
Factor (Season)	Independent (cat)	Value ‘0′: Production cycle in the dry season^3^ Value ‘1′: Production cycle in the rainy season^3^	Proportion of production cycles (out of 3) in the rainy season: Value ‘0.3′: ≤ 1 production cycle in the dry season Value ‘0.7′: ≥ 2 production cycles in the rainy season
Factor (Hatchery)	Independent (cat)	The hatchery from which the broilers originated per production cycle	Number of broiler suppliers used over 3 cycles (1–3).
t_AMU	Independent (cont)		Sum antimicrobial treatment days across the 3 production cycles prior to AMR data collection
Purpose	Independent (cont)	Proportion of AMU administered as ‘preventive’ measure per production cycle	Average proportion of AMU administered as ‘preventive’ measure across 3 production cycles prior to AMR data collection.
Cycle	Independent (cat)	Number of successive cycle (x/6)	
EmpEqui^2^	Independent (cont)	Biosecurity score for category Employment and Equipment (x/8)	
ExBio^2^	Independent (cont)	Biosecurity score for category External Biosecurity (x/12)	
FeedWater^2^	Independent (cont)	Biosecurity score for category Feed and Water management (x/14)	
IntBio^2^	Independent(cont)	Biosecurity score for category Internal Biosecurity (x/20)	
VetDrugs	Independent (cont)	Biosecurity score for category Veterinary Drug management (x/2)	
Move^2^	Independent (cont)	Biosecurity score for category Movements and Purchases (x/7)	

1 For the variable AB_count, AB represents a 3-letter code assigned to each specific antimicrobial class. The AMU model was run separately for 6 different classes: polymyxins (POL_count), fluoroquinolones (FLU_count), macrolides (MAC_count), penicillins (PEN_count), sulfonamides (SUL_count), and tetracyclines (TET_count).

2 The biosecurity score is calculated by dividing the number of questions that are answered with “yes” by the total number of questions within each category. The more questions per category are answered with “yes”, the higher the biosecurity score for that category is, indicating best practices.

3 The dry season runs from May to September, the rainy season runs from April to October.

4 Strains were considered and coded as “resistant” in the model, based on Epidemiological Cut-Off (ECOFF) values that define Non-Wild Phenotypes (NWT) (European Committee on Antimicrobial Susceptibility Testing, 2020).

***Antimicrobial Use.*** To assess the effect of our interventions on total AMU, we compared the sum of treatment days in Phase 1 and Phase 3 (Fig. 1) at production cycle level using a hierarchical Bayesian negative-binomial regression model. To allow for focusing on key predictors, we applied a sparse model with Laplace (Lasso) prior distributions, where the reciprocal of the tuning parameter followed a chi-square distribution with 1 degree of freedom. The AMU model was fitted using the R package *rstanarm* with 4 Markov chains, 4,000 iterations, and an adapt_delta of 0.99 to ensure stable sampling. The initial analysis focused on AMU per production cycle (**AB_count**, Table 2) without differentiating between antimicrobial classes.

Following this, we ran the hierarchical Bayesian negative-binomial regression model again for each separate antimicrobial class to explore class-specific patterns. The descriptive analysis showed that the use of the antimicrobial classes phosphonic acid, aminoglycosides, lincosamides, and aminocyclitols was minimal. Due to insufficient data, these classes were excluded from further modeling. As a result, models were fitted for the 6 remaining classes: polymyxins (**POL_count**), fluoroquinolones (**FLU_count**), macrolides (**MAC_count**), penicillins (**PEN_count**), sulfonamides (**SUL_count**), and tetracyclines (**TET_count**). This approach allowed for the assessment of whether the use of particular antimicrobial classes changed between Phase 1 and 3.

Independent variables were selected to evaluate whether they were associated with changes in AMU. The variables were included based on their expected relevance, informed by both literature and discussions within the research consortium. These included: cycle (**Cycle**), phase (**Phase**), season (**Season**), flock size (**N_broilers**), first week mortality (**Mortality**), percentage of preventive use (**Purpose**), Employment and Equipment (**EmpEqui**), External Biosecurity (**ExBio**), Feed and Water management (**FeedWater**), Internal Biosecurity (**Intbio**), Veterinary Drug management (**VetDrugs**), and Movements and Purchases (**Move**) (Table 2).

Due to the large number of different hatcheries recorded, hatchery was included as a random effect nested within farm (**Farm_n / Hatchery_n**) to account for clustering and to improve model convergence.

***Antimicrobial Resistance.*** To evaluate the effect of the interventions on AMR, we analyzed the mean number of tested antimicrobials (out of 14 tested) to which the commensal *E. coli* isolates from each farm exhibited NWT phenotypes (**N_resistant**). It is important to note that strains were considered and coded as “resistant” in the model, based on ECOFFs that define NWT phenotypes as described earlier. This outcome was analyzed using a Bayesian Gaussian linear model. Since AMR data were collected only once before (Phase 1) and once after (Phase 3) intervention, this analysis was conducted at farm level, rather than by production cycle as was done for the AMU model. To ensure temporal alignment between AMU and AMR data, only AMU data from the 3 production cycles immediately preceding each AMR sampling were included in our analysis on effects on AMR, resulting in 57 production cycles from Phase 1 and 42 production cycles from Phase 3.

To ensure a good model fit, the number of antimicrobial treatment days was standardized (**t_AMU**) by subtracting the overall mean from the total number of treatment days across 3 successive production cycles per farm and dividing this by the standard deviation. This produced a variable with a mean of 0 and a standard deviation of 1. For easier cross-farm comparisons, a standardized variable was also made for the number of broilers per farm (**n_broilers**) by subtracting the overall mean (across all farms) from the average number of broilers per study house per farm, divided by the standard deviation.

Given our limited sample size and the inclusion of many predictor variables, not all of which are expected to have a large effect, the model employed regularized horseshoe priors using the R package *rstanarm*. This type of regularization prior is particularly effective in high-dimensional settings where many predictors may have minimal or no influence on the outcome. This prior adjusts the global shrinkage parameter (**global_scale**) based on the expected ratio of non-zero to zero coefficients, scaled by the square root of the number of observations (Piironen and Vehtari, 2017).

The hierarchical shrinkage priors used here have very tall modes and heavy tails. This results in posterior distributions that are highly concentrated near zero unless a predictor has a strong influence on the outcome, in which case the prior exerts minimal influence, allowing the data to dominate the estimation.

This approach effectively balances the need for regularization with the flexibility to allow important predictors to have substantial effects and is therefore considered preferred over other approaches, for studies with low sample sizes and many predictors with a limited expected effect (Piironen and Vehtari, 2017).

In line with the previously described selection process, the following independent variables were included in the analysis: phase of the study (Phase), average flock size (n_broilers), average first week mortality (**Firstweekmort**), season (Season), the number of hatcheries supplying the farm (**Hatchery_unique_count**), total AMU expressed as treatment days (t_AMU), external biosecurity (ExBio), internal biosecurity (IntBio), feed and water management (FeedWater), veterinary drug management (VetDrugs), employment and equipment (EmpEqui), and movements and purchases (Move) (Table 2).

***Model fit.*** Complexity of the models was assessed using the effective number of parameters, which provides insight into how flexibly the models fit the data. Pareto-smoothed importance sampling (**PSIS**) diagnostics were used to evaluate the influence of individual observations on the models’ fit. Most observations typically fell within acceptable influence thresholds (k < 0.5), indicating stable model performance. A small proportion of observations occasionally exceeded recommended thresholds, but few—if any—entered the critical range where reliability might be compromised. Together, these diagnostics suggest that the models were generally well-behaved, not overly sensitive to outliers, and yielded reliable posterior estimates.

We evaluated the fit of the models and confirmed their adequacy based on key diagnostic metrics. Specifically, the Monte Carlo Standard Error (**MCSE**) for each parameter was small, indicating high precision in the posterior mean estimates. The potential scale reduction factor (**Rhat**) values were close to 1.00, suggesting that the model had likely converged effectively. Additionally, the effective sample size (**n_eff**) was sufficiently large, reflecting a high number of independent samples and providing confidence in the reliability of the parameter estimates. These diagnostics collectively indicate that the model performed well and provided robust estimates.

***Expression of model coefficients.*** The model coefficients are expressed as mean values, representing the central estimate of the effect derived from the posterior distribution of the model parameters. Due to the log link function, the coefficients need to be exponentiated to be interpreted in terms of the actual values.

## Results

### Antimicrobial Use

During Phase 1, the farms used 41 different VMPs containing antimicrobials: 25 of these contained 2 different antimicrobials, while 16 contained a single antimicrobial (Supplementary File 5, S5). During Phase 3, the farms used 25 different VMPs: 12 of these contained 2 different antimicrobials, and 13 contained a single antimicrobial (S5). The TF_count-based_ decreased with 18 % from 0.4 (an average of 11 treatment days per production cycle) to 0.3 (average of 9 treatment days per production cycle) during Phase 3. On average, 88 % of AMU was recorded as preventive during Phase 1, and 86 % during Phase 3.

**Fig. 2:**
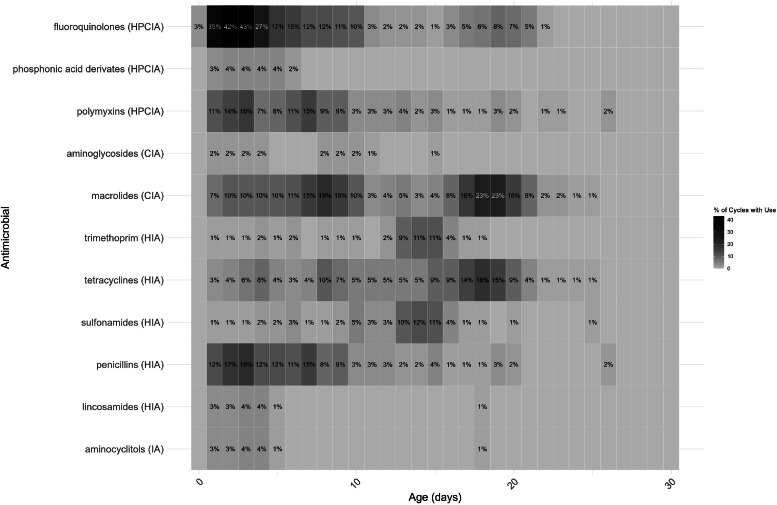
Heatmap of antimicrobial use by age during Phase 1. The use of each specific antimicrobial class per day of age was aggregated across 98 production cycles. Results are expressed as the percentage of production cycles in which this specific antimicrobial class was used per day, if the percentage exceeds 20 %, the text is white instead of black to distinguish these higher values.

**Fig. 3:**
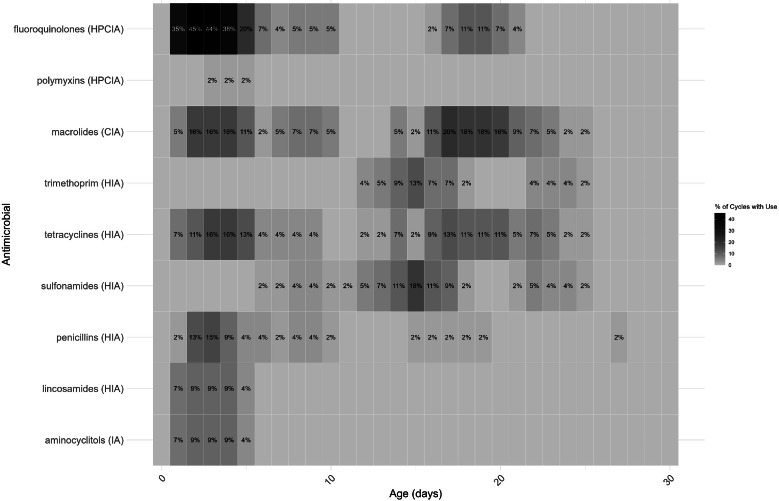
Heatmap of antimicrobial use by age during Phase 3. The use of each specific antimicrobial class per day of age was aggregated across 55 production cycles. Results are expressed as the percentage of production cycles in which this specific antimicrobial class was used per day, if the percentage exceeds 20 %, the text is white instead of black to distinguish these higher values.

A total of 11 antimicrobial classes were used in Phase 1 (Fig. 2), compared to 9 in Phase 3, with phosphonic acid and aminoglycosides only used in Phase 1 (Fig. 3). AMU varied considerably per day of age. In both Phase 1 and 3, antimicrobials were most frequently used when the broilers were 1-4 days old, and mostly fluoroquinolones were used in both phases (Figs. 2 and 3). Fig. 2, Fig. 3 also show a slight increase in AMU between days 15-20 of the production cycle in both phases.

In Phase 1, the most frequently administered antimicrobials were enrofloxacin; mean TF_count-based_ of 0.08 (2.3 treatment days per production cycle of 30 days), amoxicillin; mean TF_count-based_ of 0.05 (1.5 treatment days per cycle), and colistin; mean TF_count-based_ of 0.04 (1.3 treatment days per cycle)(S5).

In Phase 3, enrofloxacin; mean TF_count-based_ of 0.07 (2.1 treatment days per cycle), doxycycline; mean TF_count-based of_ 0.04 (1.3 treatment days per cycle), and erythromycin; mean TF_count-based_ of 0.04 (1.2 treatment days per cycle) were most frequently used. The most common antimicrobial combination in Phase 1 was colistin with amoxicillin, while in Phase 3, erythromycin in combination with doxycycline was the most frequently recorded combination (S5).

The used dosages varied considerably, both between the average dosages during Phase 1 and 3, and when compared to the (standardized) veterinary defined daily dose (**DDDvet**) as defined by the European Medicine Agency (**EMA**) (European Surveillance of Veterinary Antimicrobial Consumption ESVAC, 2016). There were also notable differences in the used dosage of specific antimicrobials between farms (Table 3). For instance, the minimum recorded dosage for erythromycin during Phase 3 was 0.06 mg/kg, while the maximum recorded dosage was 29.7 mg/kg (the range of suggested dosage on the products packaging being 75 mg/L to 400 mg/L drinking water).

**Table 3:** Used Daily Dose (UDD) calculated during Phase 1 (pre-intervention) and during Phase 3 (post-intervention).

Antimicrobial	Class	DDDvet^1^ (mg/kg)	UDD (mg/kg)		min. used dose (mg/kg)		max. used dose (mg/kg)		
			Phase 1	Phase 3	Phase 1	Phase 3		Phase 1	Phase 3
ciprofloxacin	fluoroquinolone (HPCIA)	NA	27.6	Not used	2.6	-		75.9	-
enrofloxacin	fluoroquinolone (HPCIA)	10.0	43.7	31.1	1.5	3.3		273.9	87.5
flumequine	quinolone (HPCIA)	14.0	5.2	11.4	3.7	7.5		8.8	13.7
norfloxacin	fluoroquinolone (HPCIA)	NA	Not used	6.2	-	4.6		-	7.9
fosfomycin	phosphonic acid derivate (HPCIA)	NA	21.5	Not used	11.7	-		33.4	-
colistin	polymyxin (HPCIA)	5.1	5.7	14.1	0.2	5.4		35.0	20.1
neomycin	aminoglycoside (CIA)	24.0	5.7	Not used	2.0	-		8.3	-
erythromycin	macrolide (CIA)	20.0	13.3	4.7	1.0	0.06		55.5	29.7
spiramycin	macrolide (CIA)	73.0	8.2	5.1	1.1	3.0		17.6	12.0
tylosin	macrolide (CIA)	81.0	32.9	3.2	4.7	1.2		273.9	6.5
lincomycin	lincosamide (HIA)	22.0	31.8	20.7	8.0	1.8		51.0	37.4
amoxicillin	penicillin (HIA)	16.0	39.5	48.8	0.3	8.1		203.9	167.8
sulfadiazine	sulfonamide (HIA)	34.0	26.4	25.7	6.8	0.1		41.7	39.5
sulfaquinoxaline	sulfonamide (HIA)	60.0	13.5	13.3	4.8	0.1		33.0	32.5
doxycycline	tetracycline (HIA)	15.0	8.2	5.4	0.4	0.01		37.0	22.3
oxytetracycline	tetracycline (HIA)	39.0	16.0	39.2	0.9	13.2		61.6	98.1
trimethoprim	trimethoprim (HIA)	6.4	5.3	5.4	1.4	0.7		8.3	7.9
spectinomycin	aminocyclitol (IA)	38.0	63.7	78.8	16.1	4.2		102.0	149.6

1 Defined Daily Dose (DDDvet) as defined by the European Medicine Agency (EMA) (European Surveillance of Veterinary Antimicrobial Consumption ESVAC, 2016).

### Antimicrobial resistance

Across the 14 antimicrobials tested, the isolates exhibited an average NWT phenotype for 6.2 antimicrobials during Phase 1 and 5.9 during Phase 3. The MIC distributions are summarized in Table 4. During Phase 1, the 5 antimicrobials to which isolates most frequently exhibited NWT phenotypes, in descending order, were ciprofloxacin, ampicillin, tetracycline, sulfamethoxazole, and trimethoprim. During Phase 3, these were ciprofloxacin, ampicillin, nalidixic acid, tetracycline and sulfamethoxazole. NWT phenotypes were also observed for antimicrobial classes that were not used during either Phase 1 or 3 (3rd generation cephalosporines, carbapenems, glycylcyclines, and amphenicols. For carbapenems and glycylcyclines only a few NWT phenotypes were observed.

**Table 4:** Minimum inhibitory concentration distribution for *E. coli* in fecal isolates collected during Phase 1 (pre-intervention phase) and Phase 3 (post-intervention phase).

^1^Each antimicrobial belongs to a different class, the tested antimicrobials are classified as follows: AMP is a penicillin (CIA), AZI is a macrolide (CIA), CEF and CEZ are cephalosporins (3rd, 4th generation) (HPCIA), CHL is an amphenicol (HIA), CIP is a fluoroquinolone (HPCIA), COL is a polymyxin (HPCIA), GEN is an aminoglycoside (CIA), MER is a carbapenem (HPCIA), NAL is a quinolone (HPCIA), TET is a tetracycline (HIA), TIG is a glycylcycline (CIA), TRI is a trimethoprim (HIA) and SUL is a sulfonamide (HIA).

^2^The clinical break point (CBP) according to the Clinical and Laboratory Standards institute (CLSI) (Clinical Laboratory Standards Institute (CLSI), 2024). Because no CLSI CBP is available for tigecycline only the epidemiological cut-off (ECOFF) from EUCAST was used (European Committee on Antimicrobial Susceptibility Testing, 2020).

^3^The ECOFF according to the European Committee on Antimicrobial Susceptibility Testing (EUCAST) (European Committee on Antimicrobial Susceptibility Testing, 2020).

^4^The R-CBP column indicates the percentage of resistant isolates based on the CBP. The Non-wild-type column indicates the percentage of non-wild-type isolates determined according to ECOFF. N isolates indicates the total number of isolates tested.

### Additional risk factors

Average first week mortality was similar in Phases 1 and 3, with an average of 2.1 % (ranging from 0.5 % – 9.7 %) in Phase 1 and 1.9 % (ranging from 0.4 % – 10.6 %) in Phase 3.

The mean biosecurity category score for external biosecurity determined in Phase 1 was 0.28 (ranging from 0 to 0.67), for employment and equipment 0.50 (ranging from 0.12 to 0.88), for movements and purchases 0.48 (0.14 to 1.00), for food and water management 0.69 (0.46 to 1.00), for internal biosecurity 0.58 (0.35 to 0.78), and for management of veterinary drugs 0.69 (0 – 1.00). The 4 farms (Farms 1, 2, 7, and 9), with consistently low scores across all biosecurity categories, were also among the dropped-out farms due to farming cessation or changes in management.

During Phase 1, DOCs were derived from a total of 39 unique hatcheries across the analyzed 98 production cycles. Of the production cycles during this phase, 35 took place in the dry season and 63 in the rainy season. In Phase 3, 33 unique hatcheries were recorded across 55 production cycles. Of these cycles 28 occurred in the dry season and 27 in the rainy season.

### AMU and AMR model outcomes

**Fig. 4:**
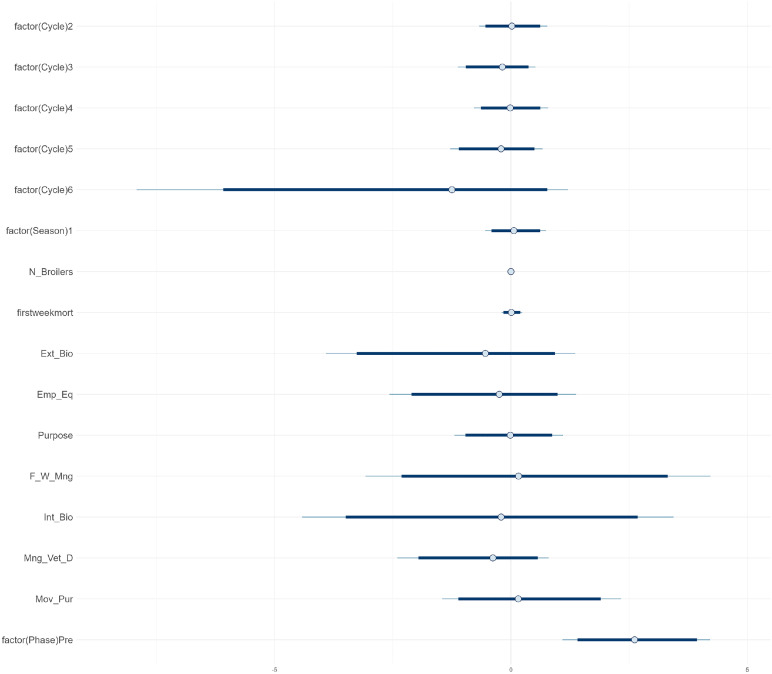
Posterior estimates from a Bayesian negative binomial mixed-effects model assessing the factors associated with the use of colistin (POL_count). The model includes random effects for farms nested within hatcheries, although hatcheries are excluded from the plot for clarity. Estimates are shown with 95 % credible intervals. The model was fitted using a lasso prior for regularization.

***AMU.*** Due to missing data on the hatchery where the DOCs originated from or missing data on first week mortality, 11 production cycles (3 from Phase 1, 8 from Phase 3) were excluded from the AMU model, resulting in a final sample of 142 production cycles included in the AMU model. The estimated intercept of the model for the overall AMU (aggregated across all antimicrobial classes) per production cycle was 2.3 (95 % credible interval (**CrI**) 1.7-2.8), representing the expected log count of AMU when all predictors are at reference levels. This corresponds to an estimated TF_count-based_ of 0.33 (i.e. 10.0 treatment days per 30-day production cycle). Most independent variables, including production cycle, season, flock size, first week mortality, and internal and external biosecurity, had posterior mean estimates close to zero on the log scale, with 95 %CrIs including both positive and negative values, indicating no consistent association with AMU. The variable “factor (Phase) Pre”, representing Phase 1, had a posterior mean of 0.0 with a 95 % CrI of 0.0 – 0.3 (Fig. 4). This suggests a possible tendency toward higher AMU before the intervention, but inclusion of 0 in the CrI indicates variability. There is thus no strong evidence for a difference in AB_count between Phase 1 and 3.

The subsequent analyses done per antimicrobial class for 6 of the most used classes in this study, i.e. polymyxins, fluoroquinolones, macrolides, penicillins, sulfonamides and tetracyclines, showed that the intervention had a modest decreasing effect on penicillin use, with a posterior mean of 0.6 and a 95 % CrI ranging from 0.0 to 1.5. While this corresponds to an estimated 82 % expected higher penicillin count in Phase 1 compared to Phase 3 (e^0.6^ ≈ 1.82) the 95 % CrI includes zero, indicating uncertainty around the direction and magnitude of the effect (S4). The most pronounced effect was observed in the polymyxins model. For polymyxins specifically, a positive association was observed between use of polymyxins and Phase 1 (pre-intervention), indicating higher usage prior to the intervention (Fig. 4). The farm-level random effects highlighted variability among farms, indicating that unobserved, farm-specific factors may contribute to differences in use of polymyxins. For polymyxins, the mean coefficient was 2.6 (95 % CrI 1.1 – 4.2), corresponding to an estimated 13.5-fold higher number of treatment days (e^2.6^) during Phase 1 compared to Phase 3.

In addition, the nested random effect of hatcheries within farms indicated minimal additional variability, suggesting that differences in use of polymyxins were largely attributable to farm-level factors rather than hatchery origin.

**Fig. 5:**
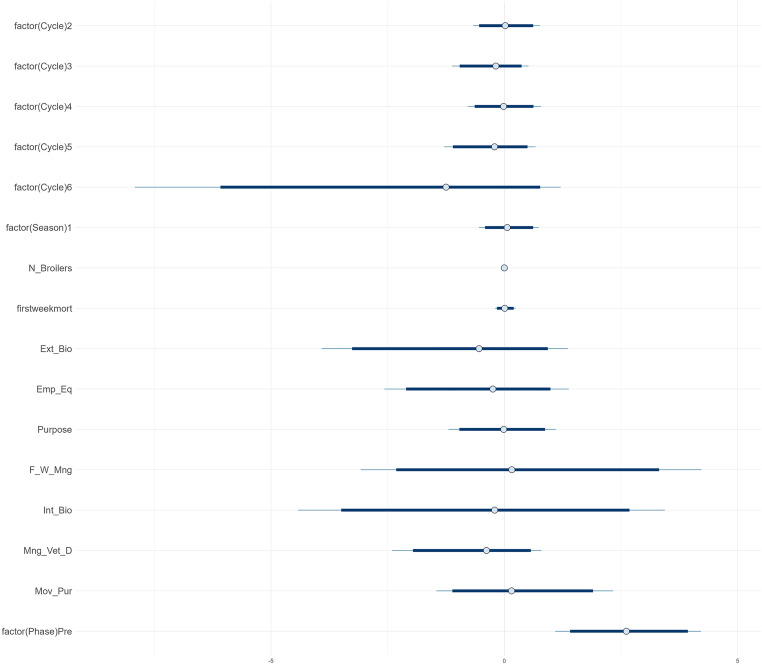
Overview of coefficient estimates^1^ for each variable included in the Bayesian Gaussian linear model with horseshoe priors assessing factors associated to AMR. The plot displays the mean estimate (filled circles), with dark blue bars indicating the 90 % credible intervals and thin light blue lines representing the 95 % credible intervals of the posterior estimates. ^1^For each predictor, the posterior mean indicates the estimated change in the number of antimicrobials to which the isolates show NWT phenotype (N_resistant) associated with a one-unit increase in the predictor, while holding other factors constant.

***AMR.*** The estimates for each predictor from the model used to evaluate the effect of the interventions on AMR are presented in Table 5 and visualized in Fig. 5. For each predictor, the posterior mean indicates the estimated change in the number of antimicrobials to which the isolates show NWT phenotype (N_resistant) associated with a one-unit increase in the predictor, while holding other factors constant. The distribution of coefficient estimates in Fig. 5 shows that many effects were small or uncertain, as indicated by the credible intervals that include zero. The intercept is the expected baseline level of resistant isolates when all predictors are at their reference levels (posterior mean 6.14, CrI 5.42-7.12).

**Table 5:** Results of the bayesian gaussian linear model with horseshoe priors assessing the factors associated to AMR.

Estimates^1^:	Mean	SD	2.5 %	97.5 %
**Intercept**	6.13	0.43	5.39	7.10
**factor (Phase)Pre (phase 1)**	0.01	0.11	−0.18	0.26
**broilers**	−0.02	0.08	−0.23	0.11
**firstweekmort**	−0.03	0.10	−0.33	0.09
**factor(Season)0.7**	0.02	0.12	−0.17	0.35
**factor(Hatchery)2**	0.02	0.12	−0.17	0.32
**factor(Hatchery)3**	0.01	0.11	−0.19	0.26
**t_AMU**	0.28	0.27	−0.02	0.81
**EmpEqui**	0.00	0.16	−0.29	0.31
**ExBio**	0.04	0.27	−0.25	0.72
**FeedWater**	−0.03	0.25	−0.59	0.28
**IntBio**	−0.04	0.32	−0.74	0.25
**factor(VetDrugs)1**	0.00	0.11	−0.22	0.25
**Move**	0.01	0.16	−0.24	0.40
**sigma**	1.09	0.16	0.82	1.44

Phase = phase of the study with Pre = Phase 1; Broilers = average number of broilers in the poultry house across 3 production cycles prior to AMR sampling; firstweekmort = average percentage of first week mortality across the 3 cycles; Season 0.7 = ≥ 2 production cycles in the rainy season; Hatchery = number of broiler suppliers used over the 3 cycles (1–3); t_AMU = sum antimicrobial treatment days across the 3 cycles; EmpEqui = biosecurity score for category Employment and Equipment; ExBio = score for External Biosecurity; FeedWater = Feed and Water management; IntBio = Internal Biosecurity; VetDrugs = Veterinary Drug management; Move = Movements and Purchases.

1 Estimates of the posterior mean, standard deviation (SD), and 95 % credible intervals (2.5 % and 97.5 %) for each predictor’s effect on the number of resistant isolates (N_resistant). The model includes both continuous and categorical predictors, with sigma representing the residual standard deviation of the model (see Table 2 for details on all predictors).

**Fig. 6:**
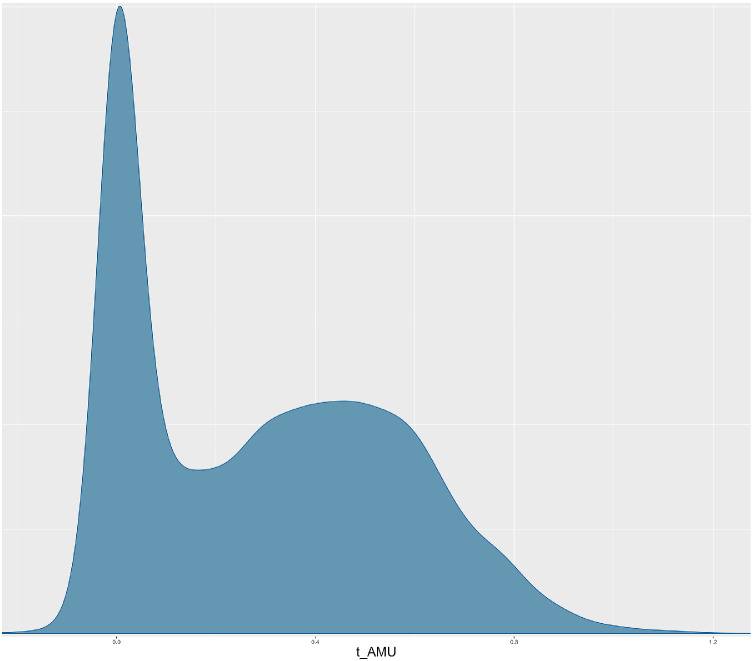
Posterior distribution of standardised total antimicrobial use (t_AMU). It shows a right-skewed tail, suggesting a potential positive association with the number of resistant isolates.

An interesting observation was the potential positive association between standardized AMU (t_AMU) and N_resistant with a posterior mean of 0.28 (95 % CrI −0.02 – 0.81) (Table 5 and Fig. 5). While this suggests that a one-unit increase in t_AMU may be associated with an increase in the number of resistant antimicrobial classes (N_resistant), the CrI includes zero, suggesting some uncertainty around this effect. The density plot of t_AMU provides a visual representation of the posterior distribution of the coefficient, including its central tendency and spread (Fig. 6). The coefficient for t_AMU shows a posterior distribution with a rightward tail, indicating that higher values fall within the 95 % CrI. This extended tail further suggests some evidence of a potential positive association between t_AMU and N_resistant, implying that an increase in antimicrobial use may be linked to a higher number of antimicrobial classes to which isolates show resistance.

## Discussion

This longitudinal intervention study focusing on stimulating prudent AMU on small- and medium-scale broiler farms in Indonesia, showed an overall 18 % decrease in observed AMU per production cycle. Average TF_count-based_ AMU decreased from 0.4 (11 treatment days per 30-day production cycle) in Phase 1 (pre-intervention) to 0.3 (9 treatment days per cycle) in Phase 3 (post-intervention). However, when assessed using a hierarchical Bayesian model, no significant overall effect of the intervention on total AMU was detected. It therefore cannot be ruled out that the observed reduction was due to chance. Among specific antimicrobial classes, a modest reduction was observed for penicillins, though the effect is uncertain (95 % CrI containing 0.0). A significant reduction was found only for colistin (polymyxins), with usage in Phase 1 approximately 13.5 times higher than in Phase 3, corresponding to a 92.6 % decrease. This reduction is most likely linked to the national ban on colistin implemented in 2020. By contrast, no significant changes were observed for other antimicrobial classes, and no other internal or external risk factors included in analyses were associated with reductions in AMU.

The AMU patterns observed in this study are in accordance with findings from neighboring countries. As reported in Vietnam and Bangladesh, fluoroquinolones, polymyxins and penicillins have been consistently identified as frequently used antimicrobial classes (Nguyen et al., 2016; Ibrahim et al., 2023b). In our study, preventive AMU remained high with 88 % in Phase 1 and 86 % in Phase 3, which aligns with previous findings across Southeast Asia where preventive AMU accounted for over 80 % of antimicrobial use (Ibrahim et al., 2023b). Even in the absence of clinical symptoms, farmers in Indonesia—like their counterparts in Vietnam and in many other countries, including previously in the Netherlands (Speksnijder et al., 2015b; Phu et al., 2020; Umair et al., 2021)—commonly consider AMU during the first week after the arrival of DOCs to be essential. This perception may be influenced by factors such as suboptimal housing conditions, distrust in DOC quality, limited access to veterinary support, and a lack of resources to implement alternative preventive measures. Evidence from Vietnam and China suggests that, due to the relatively low cost of antimicrobials, farmers may rely on antimicrobials to compensate for insufficient implementation of labor-intensive husbandry practices, such as improved biosecurity, cleaning and disinfection protocols, and the purchase of high-quality DOCs (Wu, 2019; Kompudu et al., 2020; Bao et al., 2024). In this context, antimicrobials may serve as a form of "insurance" against disease outbreaks. The perceived threat of infectious diseases is significant in the first week, which is closely linked to AMU (Ibrahim et al., 2023b).

Another notable finding was the reduction in both the diversity of VMPs used (from 41 to 25 different VMPs) and in the number of combination products containing 2 antimicrobials (from 25 to 12). The use of products containing a combination of antimicrobials is common in the poultry industry, with a study from Vietnam reporting a similarly high use (50 %) of antimicrobial products containing 2 antimicrobial compounds from different classes (Coyne et al., 2020a). It is important to note that farmers do not necessarily prefer using combination products. Farmers within our study often mentioned they did not even realize that a product contained 2 different classes. This lack of awareness is likely influenced by the over-the-counter availability of antimicrobials and the fact that advice on AMU is frequently provided by individuals without formal veterinary training (Nguyen et al., 2016; Hibbard et al., 2023). As a result, the use of antimicrobial (combination) products is not always based on specific disease symptoms or targeted treatment strategies. Although the reasons behind the observed reduction in the use of combination products remain uncertain, it is plausible that the introduction of the antimicrobial formulary, alongside repeated communication about prudent AMU and peer-to-peer learning sessions, may have contributed to more deliberate and selective antimicrobial choices by farmers. Another possible reason for this reduction could be that combination products tend to be more expensive, which may have led farmers to opt for more affordable products containing a single antimicrobial. However, given the absence of a formal control group in this study, these observations should be interpreted with caution. Further research would be valuable to better understand the drivers behind this trend.

Although the model did not detect a statistically significant effect, we assume the observed decrease in AMU reflects a trend. Overall, observed AMU decreased by 18 % in Phase 3 (post-intervention), which is a notable improvement over a relatively short period. This decrease in a relatively short time is especially meaningful when compared to countries like the Netherlands, which targeted similar reductions but also had much stronger regulatory enforcement and greater financial incentives in place (Speksnijder et al., 2015b). Between 2009 and 2011, the Netherlands implemented a national policy targeting a 20 % reduction in livestock antimicrobial use, supported by mandatory registration of all veterinary antimicrobial use, farm-level benchmarking, and sector-wide quality assurance systems (Mevius and Heederik, 2014). These measures were reinforced by the establishment of the Netherlands Veterinary Medicines Authority (SDa) to monitor usage and enforce compliance (Mevius and Heederik, 2014). We identified 1 comparable intervention study conducted in a similar country—Vietnam—which also aimed to reduce AMU in broiler production. The Vietnamese study involved smaller-scale farms (50–2,000 birds) with fewer production cycles per year, often run by less-experienced farmers and reported a 66 % decrease in AMU (Phu et al., 2020). These substantial differences in farm scale, farm layout, poultry breeds and farmer profiles make direct comparison with our study difficult. Farmers in our setting were generally more experienced and operated at larger scales with multiple production cycles annually, which may require more time and tailored support to achieve similar levels of change.

Regarding antimicrobial susceptibility testing (**AST**) for a panel of 14 antimicrobials (S1), commensal *E. coli* isolates consistently exhibited NWT phenotypes to an average of 6 antimicrobials in both phases. Notably, in Phase 3, nalidixic acid replaced trimethoprim among the top 5 antimicrobials with the highest proportion of NWT isolates. A study conducted in Bangladesh showed similarly high levels of resistance towards (fluoro)quinolones, penicillins and tetracyclines (Ibrahim et al., 2023a).

Although observed AMU decreased by 18 % in Phase 3, the average NWT phenotypes observed did not show a corresponding decline. While this may appear counterintuitive given the generally established link between AMU and AMR, such a pattern is not unexpected. As observed in other countries, like the Netherlands, changes in AMR trends often lag behind reductions in AMU (Speksnijder et al., 2015b). This highlights the importance of sustained and consistent efforts to reduce AMU, with the expectation that reductions in AMR will follow over time. Additionally, this study did not include data on AMU at the hatchery level. Previous research indicates that antimicrobial use in hatcheries can be substantial (Persoons et al., 2011) and may influence AMR outcomes, underscoring the need for a more comprehensive assessment of antimicrobial inputs across the production cycle.

An important limitation of our study was that communication between advisors and farmers was less comprehensive than intended. COVID-19 restrictions limited the ability of our extension workers to visit farms as regularly as envisioned during Phase 1, reducing opportunities to build trust and rapport. Trust is critical in influencing farmer behavior, especially when recommendations are perceived as carrying financial risks, such as a heightened risk of disease and potential productivity losses associated with reduced AMU (Phu et al., 2020).

In addition, the unplanned extension of the study (due to COVID-19) resulted in a change of extension workers delivering the AMU-related communication, further hindering the development of consistent relationships with farmers. The situation was compounded by the presence of competing advice from other actors, such as feed suppliers with longstanding relationships with farmers, making it more challenging to promote changes in established practices (Persoons et al., 2011; Phu et al., 2020).

While persuasive approaches are widely regarded as effective in achieving sustainable behavior change (Persoons et al., 2011; Phu et al., 2020), our findings suggest that regulatory interventions, such as the 2020 ban on colistin, should not be underestimated in achieving reductions in AMU.

Previous research has shown that interventions combining multiple strategies to influence behavior tend to be more effective than those relying on a single approach (Wilkinson et al., 2018; Smith et al., 2019). In line with this, our study employed several complementary strategies (Box 1). Given that AMU was concentrated primarily in the brooding phase (the first week of production), we hypothesized that improved monitoring of DOCs through structural DOC scoring would contribute to a reduction in AMU. However, evaluation revealed that none of the farmers adopted the DOC scoring tool provided. Reasons mentioned by farmers included the additional workload during the unloading process and high staff turnover on farms, which resulted in a loss of knowledge about the tool's intended use.

Similarly, the biosecurity training—widely regarded as an effective intervention for reducing AMU in other settings (Wilkinson et al., 2018; Cobb, 2021; Dhaka et al., 2023; Mallioris et al., 2023)—did not yield a measurable reduction in this study. Although the questionnaire was not repeated during Phase 3, CIVAS extension workers—who visited farms 3 times per production cycle—observed that the biosecurity tools provided during training (e.g., dedicated shoes and clothing for the broiler house, footbaths with disinfectant, and hand disinfectant) were often absent or not used.

Supporting this, an intervention study conducted by the FAO which focused on cost-effective biosecurity in Indonesia, found that the incentives to implement biosecurity measures varied greatly (Kompudu et al., 2020). For example, direct owner involvement was associated with better adherence to management practices and stronger motivation to maximize productivity and income, compared to farms primarily operated by hired employees (Indrawan et al., 2020; Kompudu et al., 2020). This is further reflected in the present study, where the 4 farms with the lowest biosecurity scores were also those that ceased farming or underwent management changes. Future interventions could consider strengthening incentives for farmers to adopt biosecurity measures, for example, through certification schemes that add market value to poultry products. One study in Vietnam suggested that some consumers are willing to pay a premium for chicken meeting specific food safety standards (Ifft et al., 2012), highlighting the potential role of market-based mechanisms in encouraging improved on-farm practices. Especially with the highly fluctuating meat prices a certificate increasing bird price could provide a good incentive (Hibbard et al., 2023; Bao et al., 2024).

To reach the NAP goal of reducing preventive AMU to below 50 %, the farms included in this study still require more guidance (Ministry of Health of the Republic of Indonesia, 2022). Although the interventions from our study show promise, further guidance is needed to support sustained changes in antimicrobial use. This includes improved access to poultry veterinarians who can provide education, not only to farmers, but also to stakeholders involved in antimicrobial distribution, particularly in cases where knowledge gaps may exist. Addressing these gaps in a collaborative and context-sensitive manner is essential for the long-term success of AMU reduction strategies.

## Conclusions

This study provides a unique insight into AMU and AMR levels on small- and medium-scale broiler farms. Despite the absence of enforcement and monitoring of regulations regarding AMU, a significant effect of the colistin ban was observed, underscoring that substantial reductions are achievable, particularly when supported by regulatory enforcement. In addition, total AMU decreased by 18 % between Phase 1 and Phase 3. These findings provide a valuable baseline for future intervention studies in similar settings, highlighting the strong potential of targeted, farm-level strategies to reduce imprudent AMU on small- and medium-scale broiler farms.

## Funding

This study was funded by 2 research grants from NWO (Dutch Research Council): “Containment of antimicrobial resistance, toward a sustainable poultry production chain in Indonesia” (grant number W 07.50.1827) and “Diagnostics for diseases in Indonesian poultry production to support antimicrobial stewardship interventions” (grant number VidW.1154.19.017).

## Ethical statement

During the recruitment process, farmers were explained that the objective of the project was to gain insights in on-farm AMU and AMR to develop recommendations to optimize AMU during an intervention trial. All farmers signed an informed consent form prior to data collection and could quit the study at any point in time. To protect participant confidentiality, all data collected on each broiler farm were anonymized, and all data handling procedures complied with General Data Protection Regulation (**GDPR**) regulations.

## CRediT authorship contribution statement

**Rianna Anwar Sani:** Writing – original draft, Visualization, Validation, Software, Project administration, Methodology, Investigation, Formal analysis, Data curation, Conceptualization. **Annisa Rachmawati:** Writing – review & editing, Resources, Project administration, Investigation, Data curation, Conceptualization. **Sunandar Sunandar:** Writing – review & editing, Validation, Resources, Project administration, Investigation, Data curation, Conceptualization. **Gian Pertela:** Writing – review & editing, Validation, Project administration, Methodology, Investigation, Data curation, Conceptualization. **Elvina J. Jahja:** Writing – review & editing, Resources, Project administration, Methodology, Funding acquisition, Conceptualization. **Imron Suandy:** Writing – review & editing, Resources, Conceptualization. **Jan van den Broek:** Writing – review & editing, Visualization, Validation, Software, Formal analysis. **Sjaak J. de Wit:** Writing – review & editing, Validation, Supervision, Methodology, Investigation, Funding acquisition, Formal analysis, Conceptualization. **Jaap A. Wagenaar:** Writing – review & editing, Visualization, Validation, Supervision, Resources, Project administration, Methodology, Investigation, Funding acquisition, Formal analysis, Conceptualization. **David C. Speksnijder:** Writing – review & editing, Visualization, Validation, Supervision, Resources, Project administration, Methodology, Investigation, Funding acquisition, Formal analysis, Data curation, Conceptualization. **Francisca C. Velkers:** Writing – review & editing, Visualization, Validation, Supervision, Resources, Methodology, Formal analysis, Conceptualization.

## Disclosures

The authors of this manuscript declare that they have no known competing financial or non-financial interests that could have influenced the work reported in this manuscript.

## Appendix Supplementary materials

Image, application 1

Image, application 2

Image, application 3

Image, application 4
