# Supplementary figures and images for: Longitudinal evaluation of interventions on antimicrobial use and antimicrobial resistance on broiler farms in West Java, Indonesia
Source: Poult Sci. 2025 Oct 27;104(12):106010. doi: 10.1016/j.psj.2025.106010 (PMC12681536; doi:10.1016/j.psj.2025.106010)

**S1: the EUVSEC plate that was used for antimicrobial susceptibility testing**
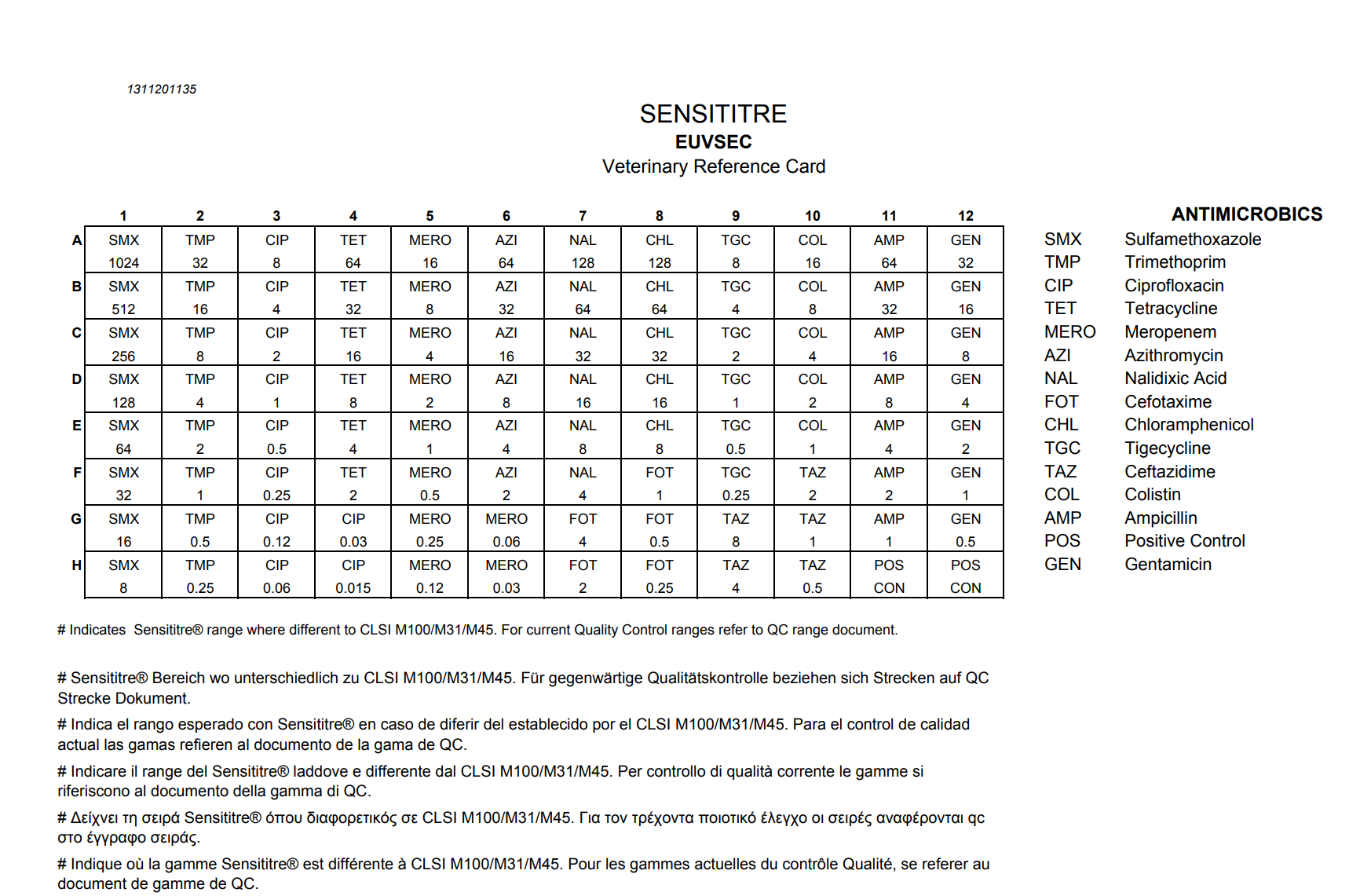

Supplement: Supplementary file 1 [file mmc1.docx]
