# Supplementary material for: Longitudinal evaluation of interventions on antimicrobial use and antimicrobial resistance on broiler farms in West Java, Indonesia
Source: Poult Sci. 2025 Oct 27;104(12):106010. doi: 10.1016/j.psj.2025.106010 (PMC12681536; doi:10.1016/j.psj.2025.106010)
Supplement: Supplementary file 2 [file mmc2.docx]

**S2: Laboratory protocol for sample processing in the lab for *E. coli***

**Introduction**

Samples will arrive in a plastic bag with 1 pair (or 2 when we need more material) of boot socks with material collected in the houses (combined feces and bedding).

The samples must be processed on the day of arrival, or the day thereafter. Make sure to keep the sample overnight at 4 °C if they are not processed the same day.

This protocol describes the processing of samples:

1. storage in 4 tubes
2. isolation of 25 *E. coli* strains per sample (this can be done immediately but can also be done from the frozen sample mentioned under ‘1’).

**Coding of the sample**

Give the sample a unique lab number and include in LIMS the sample code written on the bag.

**Transfer of sample to container**

Use gloves and work in a safety cabinet to transfer all material from the boot socks to a sterile container (maybe a stomacher bag works the best for mixing). Mix **very** well with a wooden stick (or comparable instrument) or manually when it is in a plastic bag to be sure that you store and inoculate really a pooled sample.

Weigh the sample and register this in the logbook (to know for the future if there is sufficient material).

**Storage**

The feces sample will be stored in 4 tubes (2 duplicates).

1. 2 tubes without preservation (for metagenomics in a later stage)

Label 2 tubes for metagenomics and weigh 2 times (for 2 separate tubes) 1.8 gram material and put this in a 2 mL Eppendorf tube in the -80 °C (for metagenomics).

1. 2 tubes with pepton-glycerol (the same medium you use for storage of strains) to be able to isolate bacteria in a later stage.

Label 2 tubes and weigh 2 times (for 2 separate tubes) 0.75 gram feces and mix this 0.75 gram material with 0.75 mL pepton-glycerol in 2 mL Eppendorf tubes (do this 2 times for 2 tubes). Freeze at -80 °C.

***E. coli* isolation**

We need to determine the resistance at farm-level and therefore perform AST on 25 separate *E. coli* colonies from a primary isolation plate (to compensate for loss of strains we store 30 isolates) This can be done from the frozen glycerol-sample (as described above under ‘’storage’’) but also from the fresh sample at arrival (parallel to the *Salmonella* isolation).

1. Take 1 loop (from fresh sample of frozen sample) and plate onto 5 McConkey plates. Plate well for single colonies to find 30 single colonies after incubation => the first part of the inoculation should be small, the 2^nd^ and 3^rd^ streak on a plate has more space to find the 30 single colonies.
2. Incubate at 37 °C
3. Subculture 30 separate single suspected *E. coli* colonies onto blood agar or PCA (store the McConkey plate at 4 °C for back-up in case not all 30 colonies are confirmed as *E. coli*). **Do not take the colonies from the last streak** but if possible also from the 1^st^ or 2^nd^ streak what increases the chance for diversity. Mark the colony you take on the plate.
4. Subculture onto blood agar or PCA.
5. Confirm the colonies as *E. coli* with the indol test from a single colony (mark the colony that have been subcultured)
6. If some strains are not *E. coli*, pick new strains from the stored McConkey plate (that is why you have to mark the picked colonies under ‘3’)
7. Confirm the strains as *E. coli* until you have 30 strains.
8. Subculture all strains from the indol-confirmed colony to a blood agar or PCA to be sure to freeze a pure culture.
9. Freeze all 30 strains separately with glycerol at -80 °C

**Susceptibility testing**

From the 25 *E. coli* strains **per farm** susceptibility testing has to be done using the Sensititer plates (EUSVAC plates). These plates will be provided, and a specific protocol (with the control strains) will be provided.

For *E. coli* 25 farms * 25 isolates per farm = 625 strains will be tested. In the intervention phase of the project (2022-2023) a next batch will be tested using the same procedure.

**Outcome**

The outcome of the testing is: per farm the susceptibility level (% resistance of 25 *E. coli* strains) for all antimicrobials in the EUSVAC plate. Resistance-% will be compared with antimicrobial usage patterns on the farm.
