# Supplementary material for: Longitudinal evaluation of interventions on antimicrobial use and antimicrobial resistance on broiler farms in West Java, Indonesia
Source: Poult Sci. 2025 Oct 27;104(12):106010. doi: 10.1016/j.psj.2025.106010 (PMC12681536; doi:10.1016/j.psj.2025.106010)
Supplement: Supplementary file 3 [file mmc3.docx]

**S3: The questionnaire that was used for this study, including questions on biosecurity**

| **Questions** | **Comments** |
| --- | --- |
| **Farm code** | Farm characteristic |
| **Name of farm** | Farm characteristic |
| **House ID** | Farm characteristic |
| **Name extension worker** | Farm characteristic |
| **Date of visit** | Farm characteristic |
| **Name of respondent** | Not relevant for this study |
| **Position or function of the respondent** | Farm characteristic |
| **Maps coordinate** | Farm characteristic |
| **Full-Time workers** | Farm characteristic |
| **Part-Time workers** | Farm characteristic |
| **No. of cycles and stocking density** |  |
| **Unoccupied period duration (from one cycle to the next cycle) on study house (days)** | Not included in the models |
| **The study house currently stocked with DOC?** | General characteristics not included in models of this paper (**GC not included**) |
| **Chick-in date** | GC not included |
| **DOC supplier on current period** | GC not included |
| **Breed** | GC not included |
| **Population density (number of DOC placed in the study house)** | Not always convertible to birds/m2 therefore excluded |
| **Number of cycles (batches) per year of the whole farm?** | GC not included |
| **Current population on the whole house** | GC not included |
| **Farm capacity (bird)** | GC not included |
| **Feed** |  |
| **Pre-starter feed** | GC not included |
| **Used from ... days old until ... days old** | GC not included |
| **Medicated feed** | GC not included |
| **Starter feed** | GC not included |
| **Used from ... days old until ... days old2** | GC not included |
| **Medicated feed3** | GC not included |
| **Starter feed pellet** | GC not included |
| **Used from ... days old until ... days old3** | GC not included |
| **Medicated feed2** | GC not included |
| **Feed supplement** | GC not included |
| **Feed additives** | GC not included |
| **External biosecurity** |  |
| 1. **During working days, how many workers get into the house?** | GC not included |
| 1. **Limiting access for visitors?** | Included |
| 1. **Phone number on entrance gate** | Included |
| 1. **Employees, visitors, and vehicles required to park in the designated areas at the entrance to the farm and away from all bird housing areas?** | Included |
| 1. **Visitors accompanied by worker from farm** | Included |
| 1. **Visitors’ restriction from bird housing areas and from contacting or handling your birds** | Included |
| 1. **All individuals wash hands with soap and water first before any animal contact** | Included |
| 1. **Provide clean coveralls and disposable or disinfected rubber boots for visitors** | Included |
| 1. **Provide facilities and equipment (pressure washers, brushes, hoses) for cleaning and disinfecting vehicles, boots, etc** | Included |
| 1. **The delivery vehicles and personnel required to follow the farm biosecurity guidelines (parking, driving, and animal contact)?** | Included |
| 1. **Inspection for delivery vehicles for cleanliness and restrict entry to those with visible contamination on tires, wheel wells, etc** | Included |
| 1. **Log sheet to record any visitors or vehicles that come onto your farm** | Included |
| 1. **Warning signs telling visitors to enter the farm only with permission** | Included |
| **Employees & equipment** |  |
| 1. **Talk to your employees about the disease risks associated with owning or handling birds outside of your operation** | Included |
| 1. **Employees that have contact with birds at other locations (including their own home) are required to have strict biosecurity measures while on the farm (e.g., provide them with clean boots, coveralls, headgear to wear** | Included |
| 1. **Educated yourself and trained your employees to recognize and report diseases** | Included |
| 1. **Wear clean clothes or coveralls, gloves, headgear, boots, etc. when coming in contact with birds** | Included |
| 1. **After contacting other birds, do the workers wash and disinfect boots, change gloves, headgear, and coveralls before returning to your farm** | Included |
| 1. **Clean equipment, boots, and change clothing between bird houses, especially if there any health status and age difference between one house to another house** | Included |
| 1. **Restrict the sharing of equipment between farms** | Included |
| 1. **If the equipment must be shared, is feces, feather, and base house cleaned, then equipment cleaned with warm water and soap, rinsed, disinfection, and re-rinsed before use to bird in your farm** | Included |
| **Movements & purchases** |  |
| 1. **Chickens grouped by their size during the production cycle (if yes, specify on which age and on which criteria** | Included |
| 1. **Follow all-in/all-out policy for bird houses to minimize disease introduction and allow for cleaning and disinfection** | Included |
| 1. **If the all-in/all-out policy is not possible, do you limit the frequency and number of new introductions? How?** | Included |
| 1. **Do you limit purchases to a few sources with known and trusted flock health programs?** | Included |
| 1. **Obtain new birds from a known disease-free source (known trader, commercial company, based on veterinary advice** | Included |
| 1. **Obtain a complete health history of the parent flock prior to introducing new birds** | Included |
| 1. **Request copies of vaccination and treatment records for all purchased birds** | Included |
| **Feed and water management** |  |
| 1. **Feed storage (separated storage, in house)** | Included |
| 1. **Applied FIFO (first in – first out) procedure on feeding** | Included |
| 1. **Warehouses have good walls and roofs so that the feed remains dry** | Included |
| 1. **Warehouse use pallet as feed storage base** | Included |
| 1. **Warehouse safe from rats and other pests** | Included |
| 1. **Feeder cleaned daily** | Included |
| 1. **Amount of feed given according to the age of the chicken** | Included |
| 1. **Clean and fresh feed always provide in feed area** | Included |
| 1. **Farmers use feed additives** | Included |
| 1. **All drinking places contain clean water, water is replaced twice a day** | Included |
| 1. **Water usage recorded daily** | Included |
| 1. **Do you use water storage for later use** | Included |
| 1. **Water quality testing conducted at least once a year** | Included |
| 1. **Is there any treatment to improve water quality based on the test results** | Included |
| **Internal biosecurity & disease management** |  |
| 1. **Monitor and inspect birds for signs of illness at least daily** | Included |
| 1. **Do you investigate all birds with unusual signs or those unresponsive to treatment, especially those that die suddenly** | Included |
| 1. **Immediately isolate sick birds from the flock to minimize disease spread** | Included |
| 1. **Are your isolation and quarantine facilities removed from all other bird areas and separate from one another** | Included |
| 1. **Do you use separate facilities, equipment, and staff to handle isolated birds?** | Included |
| 1. **If it is not possible to use separate facilities, equipment and staff, do you handle or visit the isolated birds LAST?** | Included |
| 1. **Clean and disinfect all equipment, clothing, boots, etc. that come into contact with ill and isolated birds** | Included |
| 1. **Prevent the sharing of ventilation, feed/water, and equipment between isolated or quarantined birds and others** | Included |
| 1. **Immediately culling the birds that show no signs of recovery** | Included |
| 1. **Immediately remove dead birds every day** | Included |
| 1. **Dead birds’ disposal** | GC not included |
| 1. **Veterinarian conduct necropsy to the birds that died from unknown causes** | Included |
| 1. **Are chickens allowed outside the house/pen?** | Included, but recoded: answering “yes” to this question is seen as not good for biosecurity, answering “no” is seen as contributing to better biosecurity |
| 1. **How do you prevent your birds from having contact with free roaming animals/birds (e.g. wildlife, cats, dogs, etc.)?** | Due to open ended nature of this question difficult to quantify the answer as a binary response, therefore Not included in the models |
| 1. **Do you maintain a rodent control program** | Included |
| 1. **How often do you see live/dead rodents in your farm** | Not included in the models |
| 1. **How often do you see wild birds in your farm** | Not included in the models |
| 1. **Do you clean and disinfect the whole house, the crates, and other poultry containers or equipment before and after use** | Included |
| 1. **Do you always clean & disinfect houses after harvest** | Included |
| 1. **Do you have a farm disinfection and cleaning protocol after harvest** | Included |
| 1. **Minimum duration of empty house between two production rounds (days)** | Not included in the models |
| 1. **Is litter quality checked every day?** | Included |
| 1. **How often do you add new litter in the house every period?** | Not included in the models |
| 1. **Do you always allow a disinfection solution contact time to “sit” and work?** | Included |
| 1. **Do you refer to the disinfectant label to determine the amount of contact time that is recommended?** | Included |
| 1. **Where were the feces disposed? And how was feces processed?** | Not included in the models |
| **Disinfection** |  |
| 1. **Name of the product** | GC not included |
| 1. **Supplier** | GC not included |
| 1. **Disinfectant and water ratio (mL disinfectant: Liter water)** | GC not included |
| 1. **When is the product used?** | GC not included |
| 1. **Name product** | GC not included |
| 1. **Supplier** | GC not included |
| 1. **Disinfectant and water ratio (mL disinfectant: Liter water)** | GC not included |
| 1. **When is the product used?** | GC not included |
| 1. **Name product** | GC not included |
| 1. **Supplier** | GC not included |
| 1. **Disinfectant and water ratio (mL disinfectant: Liter water)** | GC not included |
| 1. **When is the product used?** | GC not included |
| **Management of veterinary drugs** |  |
| 1. **How often do you read label directions for proper storage of vaccines and medications?** | GC not included |
| 1. **Do you have treatment protocols for veterinary medicines and vaccines? Can you show them?** | Included |
| 1. **Where and how do you store medicines and vaccines?** | GC not included |
| 1. **How often do you monitor your supply refrigerator to help ensure the products are adequately stored (36-46°F/2-8°C)?** | GC not included |
| 1. **How do you train employees to properly handle vaccines and medicines?** | GC not included |
| 1. **Do you restrict vaccine and medicine access to only trained personnel?** | Included |
| **Therapy failures** |  |
| 1. **Have you experienced an antibiotic treatment failure in your birds in the last 6 months (birds being treated with an antibiotic, but which did not cure the disease)?** | Subjective question and therefore excluded from the models (differs per farmer what they view as “treatment failure”) |
| 1. **If yes: Trade name or active substance** | GC not included |
| 1. **What was the main cause of the antibiotic therapy not being successful?** | GC not included |
| 1. **If yes: Product name or active substance** | GC not included |
| 1. **What was the main cause of the antibiotic therapy not being successful?** | GC not included |
| 1. **If there is treatment failure after you have treated your chickens with an antibiotic and the chickens are already at age. What do you normally do?** | GC not included |
| 1. **Have you performed sensitivity testing in the last 6 months?** | GC not included |
| 1. **When have you done sensitivity testing?** | GC not included |
| 1. **Where was sensitivity testing being conducted?** | GC not included |
