# Supplementary material for: Longitudinal evaluation of interventions on antimicrobial use and antimicrobial resistance on broiler farms in West Java, Indonesia
Source: Poult Sci. 2025 Oct 27;104(12):106010. doi: 10.1016/j.psj.2025.106010 (PMC12681536; doi:10.1016/j.psj.2025.106010)
Supplement: Supplementary file 4 [file mmc4.docx]

# **S4: Statistical analysis**

Two distinct models were used, the first to analyse the effects of the interventions on AMU, and the second to analyse these effects on AMR.

Model complexity was assessed using the effective number of parameters, which provides insight into how flexibly the model fits the data. Pareto-smoothed importance sampling (PSIS) diagnostics were used to evaluate the influence of individual observations on the model’s fit. The majority of observations typically fell within acceptable influence thresholds (k < 0.5), indicating stable model performance. A small proportion of observations occasionally exceeded recommended thresholds, but few—if any—entered the critical range where reliability might be compromised. Together, these diagnostics suggest that the models were generally well-behaved, not overly sensitive to outliers, and yielded reliable posterior estimates.

To assess the quality and reliability of the Bayesian model estimates, we evaluated standard Markov Chain Monte Carlo (MCMC) diagnostics. The Monte Carlo Standard Error (MCSE) provides an indication of the uncertainty around the estimated posterior means due to the finite number of simulations; small values suggest precise estimates. The potential scale reduction factor (Rhat) measures convergence across MCMC chains; values close to 1.00 indicate that the chains have likely converged to the target distribution. The effective sample size (n_eff) reflects how many effectively independent draws were obtained for each parameter; higher values suggest more reliable and stable estimates. These diagnostics offer important insights into the performance of the model and the validity of the inferences drawn. Detailed results for each model are presented at the end of this document.

1. ***AMU models***

For AMU, we first ran one overall model using total antimicrobial use (AB_count) as the outcome. Subsequently, we ran separate models for each antimicrobial class to explore class-specific patterns (Table 1, in the manuscript). A hierarchical Bayesian negative-binomial regression model was used, which accounts for variation between farms and hatcheries by including nested random effects for farm and hatchery. To focus on key predictors, we applied a sparse model with Laplace prior distributions, where the reciprocal of the tuning parameter followed a chi-square distribution with one degree of freedom. The model was fitted using the rstanarm package in R.

Subsequently, we ran the hierarchical Bayesian negative-binomial regression model again for each separate antimicrobial class to explore class-specific patterns. The descriptive analysis (Table 1) showed that the use of the antimicrobial classes fosfomycin (FOS), aminoglycosides (AMI), lincosamides (LIN), and aminocyclitols (AMI_IA) was minimal. Due to insufficient data, these classes were excluded from further modelling. As a result, models were fitted for the six remaining classes: polymyxin (POL), fluoroquinolones (FLU), macrolides (MAC), penicillin (PEN), sulphonamides (SUL), and tetracyclines (TET).

Table 1 Overview of how many times each antimicrobial class was used

| Count AMU | 0 |  | 1 | 2 | 3 | 4 | 5 | 6 | 7 | 8 | 9 | 10 | 11 |
| --- | --- | --- | --- | --- | --- | --- | --- | --- | --- | --- | --- | --- | --- |
| POL | 114 |  | 3 | 2 | 20 | 9 | 2 | 1 | 2 |  |  |  |  |
| FLU | 69 |  | 2 | 9 | 18 | 28 | 2 | 2 | 7 | 3 | 5 | 8 |  |
| MAC | 76 |  | 5 | 5 | 18 | 26 | 2 | 1 | 4 | 12 | 3 | 1 |  |
| FOS | 149 |  |  |  |  |  | 3 | 1 |  |  |  |  |  |
| AMI | 149 |  |  |  |  | 4 |  |  |  |  |  |  |  |
| PEN | 95 |  | 5 | 4 | 33 | 13 | 3 |  |  |  |  |  |  |
| SUL | 122 |  |  | 2 | 16 | 7 | 3 |  | 1 | 2 |  |  |  |
| LIN | 144 |  |  |  |  | 8 | 1 |  |  |  |  |  |  |
| TET | 88 |  | 7 | 6 | 11 | 29 | 3 | 4 | 2 | 2 |  |  | 1 |
| TRI | 130 |  |  |  | 14 | 6 | 2 |  | 1 |  |  |  |  |
| AMI_IA | 144 |  |  |  |  | 8 | 1 |  |  |  |  |  |  |

***1.1 Overall AMU hierarchical Bayesian negative-binomial regression model (without distinction between antimicrobial classes)***

The model used for analysing overall AMU was:

summary(fit.amu,probs=c(0.025,0.975))

Model Info:

function: stan_glmer

family: neg_binomial_2 [log]

formula: T_AMU ~ factor(Cycle) + factor(Season) + N_Broilers + firstweekmort +

Ext_Bio + Emp_Eq + Purpose + F_W_Mng + Int_Bio + Mng_Vet_D +

Mov_Pur + factor(Phase) + Purpose + (1 | Farm_n/Hatchery_n)

algorithm: sampling

sample: 8000 (posterior sample size)

priors: see help('prior_summary')

observations: 142

Model output can be found in the main text.
Model checks revealed a good fit:

> y_loo

Computed from 8000 by 142 log-likelihood matrix

Estimate SE

elpd_loo -432.8 8.8

p_loo 26.5 3.4

looic 865.6 17.6

------

Monte Carlo SE of elpd_loo is NA.

Pareto k diagnostic values:

Count Pct. Min. n_eff

(-Inf, 0.5] (good) 117 82.4% 1444

(0.5, 0.7] (ok) 23 16.2% 570

(0.7, 1] (bad) 2 1.4% 49

(1, Inf) (very bad) 0 0.0% <NA>

See help('pareto-k-diagnostic') for details.


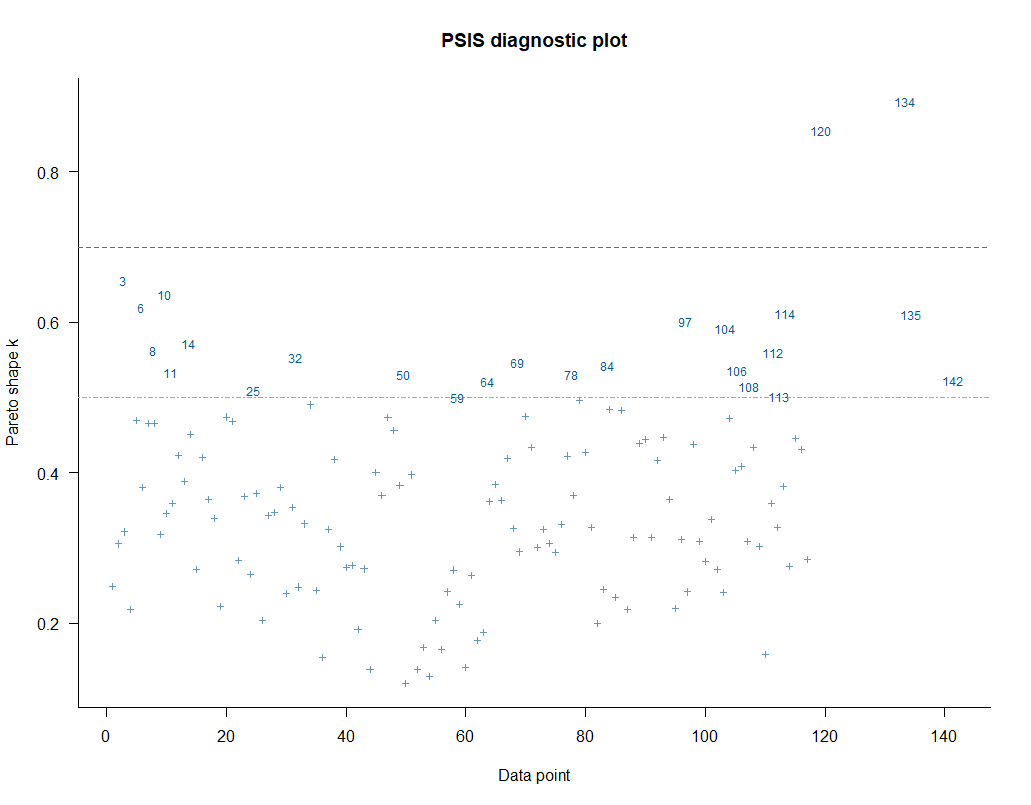


Figure 1 PSIS diagnostic plot for the total AMU hierarchical Bayesian negative-binomial regression model

***1.2 Polymyxin hierarchical Bayesian negative-binomial regression model***

The model used for analysing specific polymyxin use was:

fit.pol <- stan_glmer(

POL_count ~ factor(Cycle) + factor(Season) + N_Broilers +

firstweekmort + Ext_Bio + Emp_Eq + Purpose +

F_W_Mng + Int_Bio + Mng_Vet_D + Mov_Pur + factor(Phase) + Purpose +

(1 | Farm_n / Hatchery_n),

data = d_aggr,

family = neg_binomial_2(), # Specify the negative binomial family

chains = 4,

iter = 4000,

adapt_delta = 0.99,

prior = lasso(df = 1, location = 0, scale = NULL, autoscale = TRUE)

)

Model output can be found in the main text.
Model checks revealed a good fit:

y_loo

Computed from 8000 by 142 log-likelihood matrix

Estimate SE

elpd_loo -159.1 17.1

p_loo 18.6 3.5

looic 318.2 34.2

------

Monte Carlo SE of elpd_loo is NA.

Pareto k diagnostic values:

Count Pct. Min. n_eff

(-Inf, 0.5] (good) 126 88.7% 870

(0.5, 0.7] (ok) 12 8.5% 158

(0.7, 1] (bad) 4 2.8% 53

(1, Inf) (very bad) 0 0.0% <NA>

See help('pareto-k-diagnostic') for details.


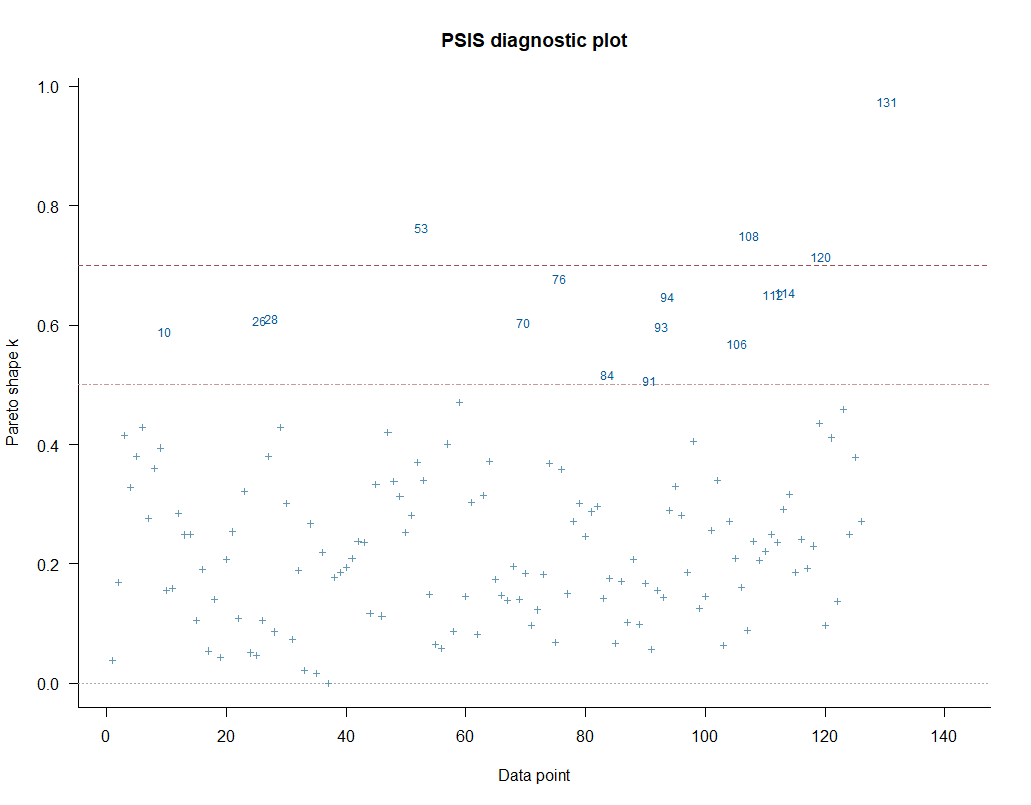


Figure 2 PSIS diagnostic plot of Polymyxin hierarchical Bayesian negative-binomial regression model

- 1. ***Fluoroquinolone hierarchical Bayesian negative-binomial regression model:***

Model output:


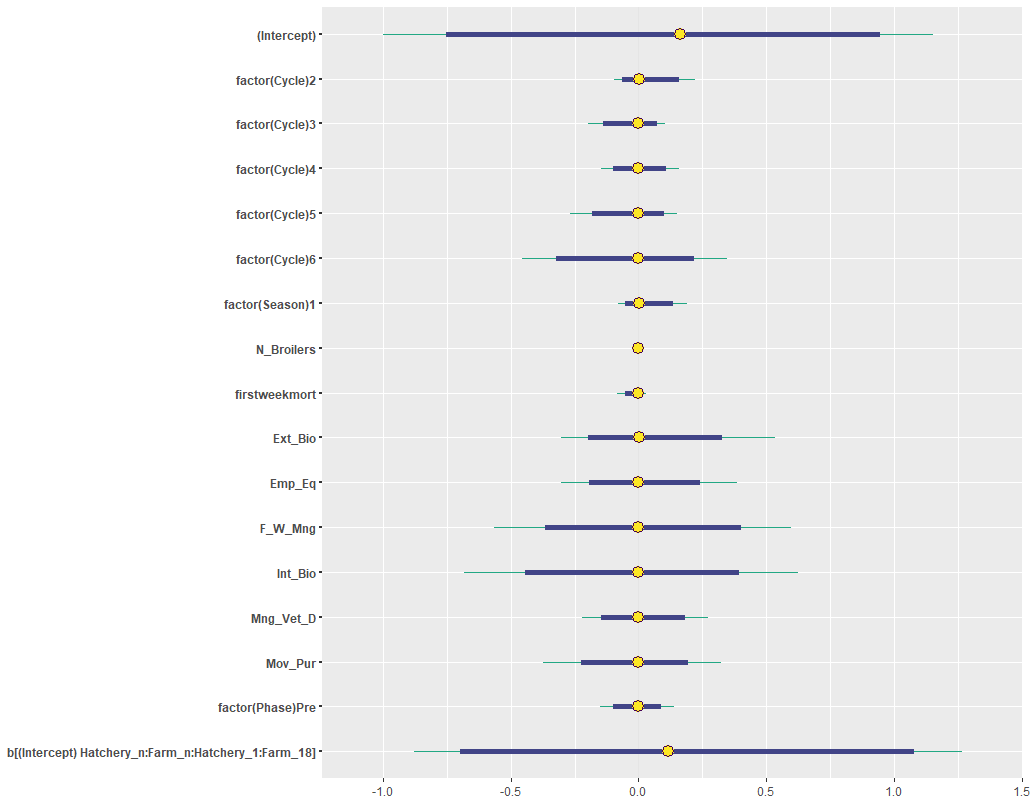


Figure 3 model output of fluoroquinolone hierarchical Bayesian negative-binomial regression model

Model checks revealed a good fit:

| y_loo  Computed from 8000 by 143 log-likelihood matrix  Estimate SE  elpd_loo -252.8 17.5  p_loo 38.6 7.8  looic 505.6 34.9  ------  Monte Carlo SE of elpd_loo is NA.  Pareto k diagnostic values:  Count Pct. Min. n_eff  (-Inf, 0.5] (good) 104 72.7% 1202  (0.5, 0.7] (ok) 30 21.0% 809  (0.7, 1] (bad) 8 5.6% 51  (1, Inf) (very bad) 1 0.7% 2  See help('pareto-k-diagnostic') for details.  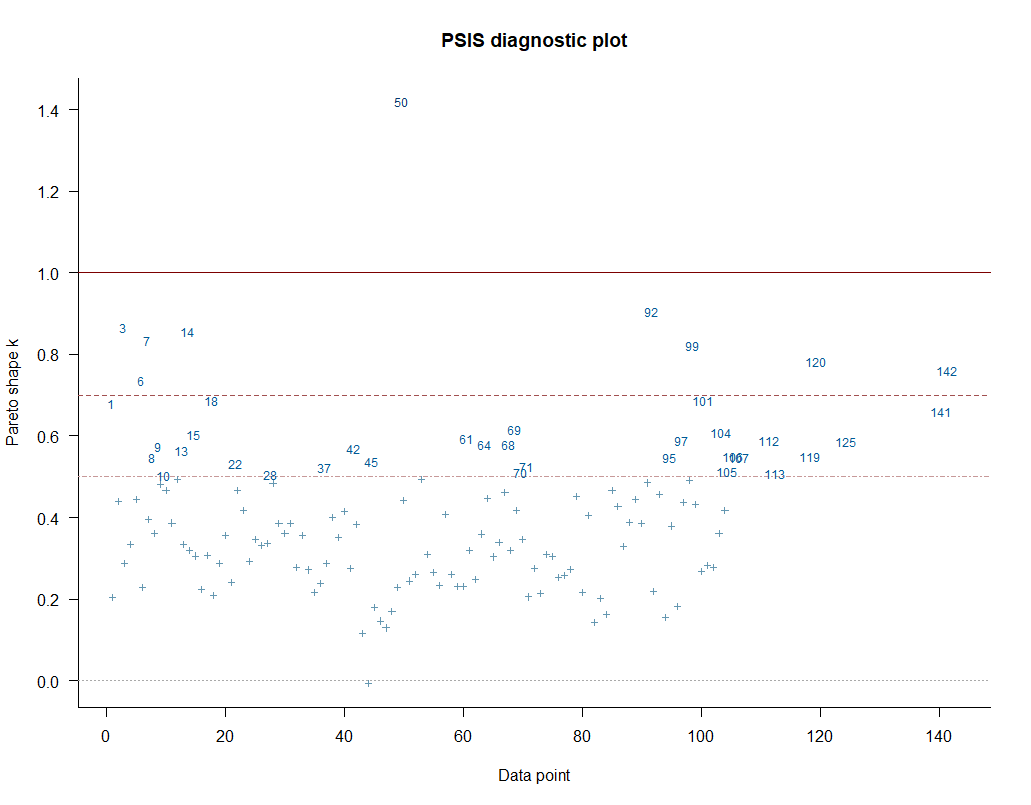  Figure 4 PSIS diagnostic plot of fluoroquinolone hierarchical Bayesian negative-binomial regression model |
| --- |
| - 1. ***Macrolide hierarchical Bayesian negative-binomial regression model:***   Output model:  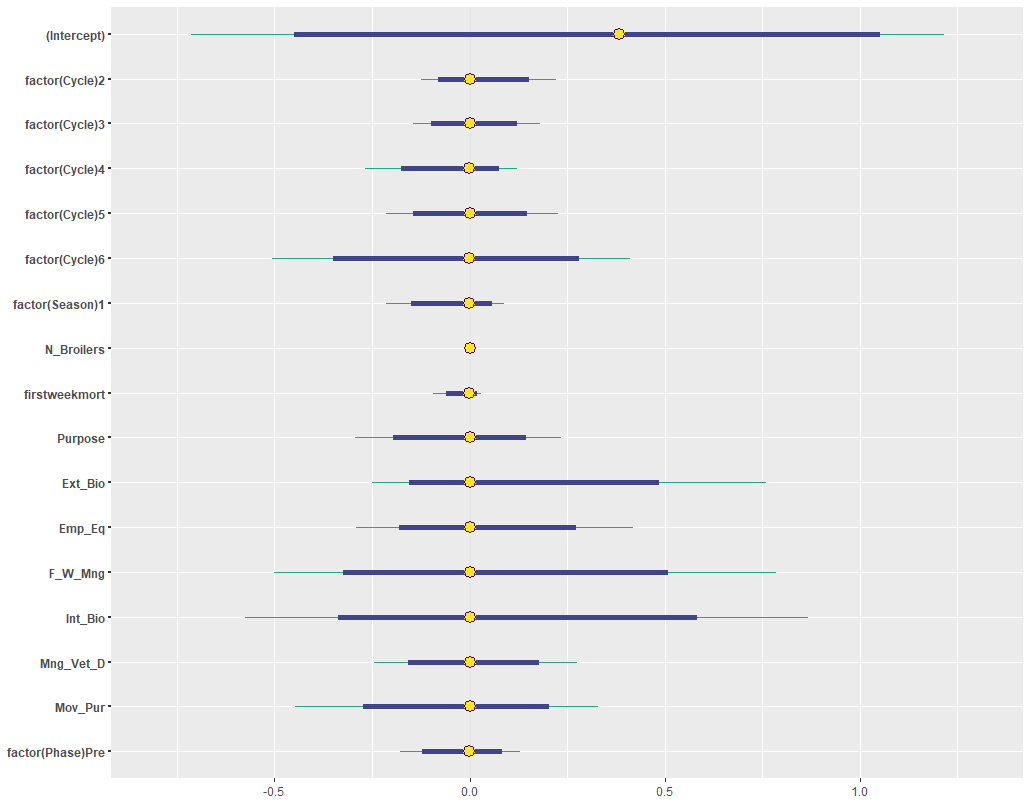  Figure 5 model output of macrolide hierarchical Bayesian negative-binomial regression model  Model checks revealed a good fit:  y_loo  Computed from 8000 by 142 log-likelihood matrix  Estimate SE  elpd_loo -265.3 13.6  p_loo 27.8 2.5  looic 530.6 27.2  ------  Monte Carlo SE of elpd_loo is NA.  Pareto k diagnostic values:  Count Pct. Min. n_eff  (-Inf, 0.5] (good) 93 65.5% 1022  (0.5, 0.7] (ok) 43 30.3% 97  (0.7, 1] (bad) 6 4.2% 145  (1, Inf) (very bad) 0 0.0% <NA>  See help('pareto-k-diagnostic') for details.  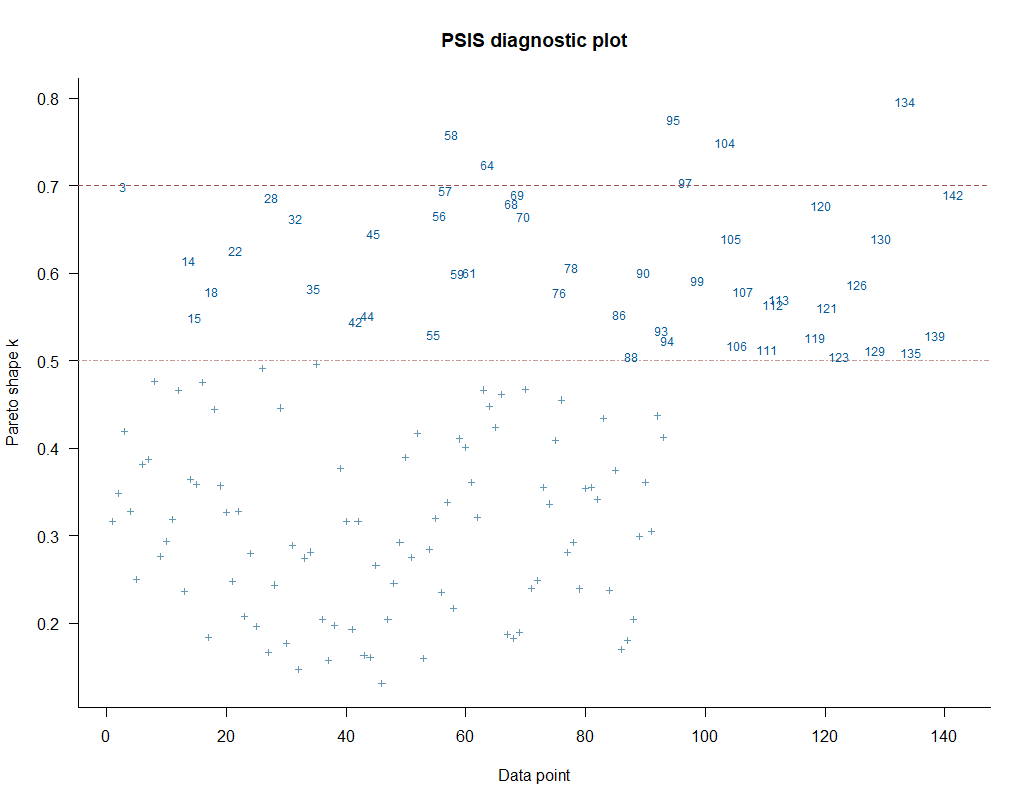  Figure 6 PSIS diagnostic plot of macrolide hierarchical Bayesian negative-binomial regression model |
| \|  \| \| --- \| |

- 1. ***Penicillin hierarchical Bayesian negative-binomial regression model:***

Model output:


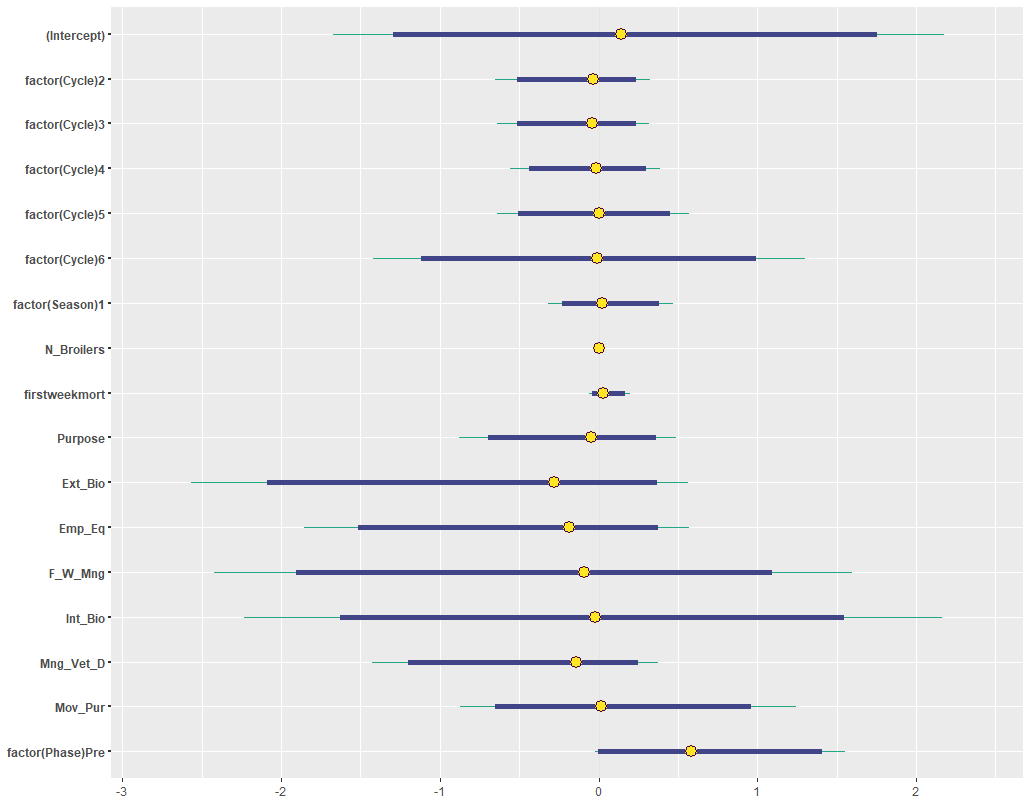


Figure 7 model output of penicillin hierarchical Bayesian negative-binomial regression model

Model checks revealed a good fit:

> y_loo

Computed from 8000 by 142 log-likelihood matrix

Estimate SE

elpd_loo -203.7 15.1

p_loo 20.2 2.8

looic 407.4 30.3

------

Monte Carlo SE of elpd_loo is NA.

Pareto k diagnostic values:

Count Pct. Min. n_eff

(-Inf, 0.5] (good) 116 81.7% 877

(0.5, 0.7] (ok) 23 16.2% 568

(0.7, 1] (bad) 3 2.1% 28

(1, Inf) (very bad) 0 0.0% <NA>

See help('pareto-k-diagnostic') for details.


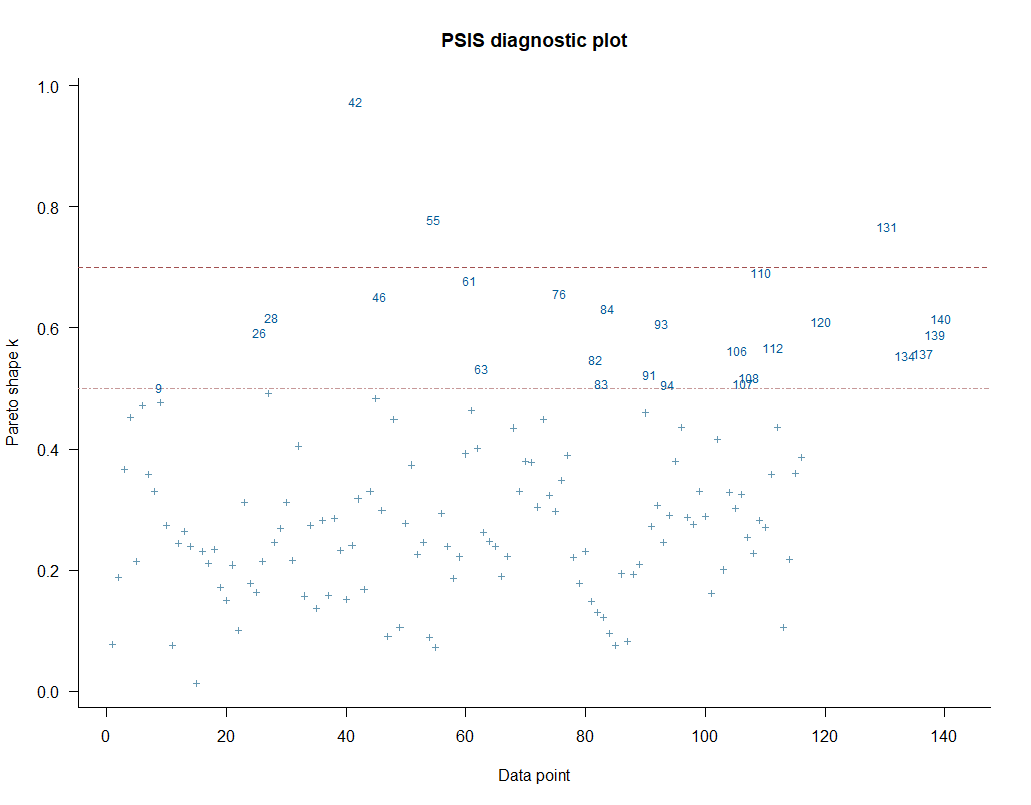


Figure 8 PSIS diagnostic plot of penicillin hierarchical Bayesian negative-binomial regression model

- 1. ***Sulphonamide hierarchical Bayesian negative-binomial regression model:***

Output model:


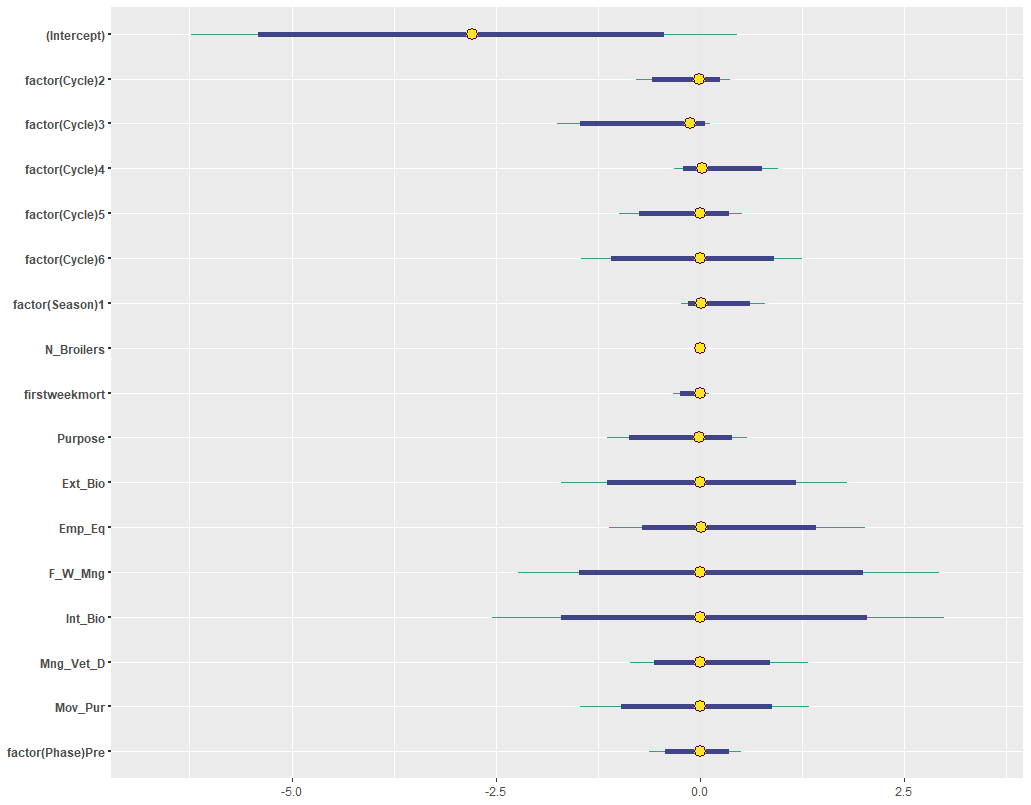


Figure 9 model output of sulphonamide hierarchical Bayesian negative-binomial regression model

Model checks revealed a good fit:

> y_loo

Computed from 8000 by 142 log-likelihood matrix

Estimate SE

elpd_loo -115.7 16.0

p_loo 27.5 5.2

looic 231.4 31.9

------

Monte Carlo SE of elpd_loo is NA.

Pareto k diagnostic values:

Count Pct. Min. n_eff

(-Inf, 0.5] (good) 69 48.6% 1499

(0.5, 0.7] (ok) 49 34.5% 197

(0.7, 1] (bad) 22 15.5% 21

(1, Inf) (very bad) 2 1.4% 8

See help('pareto-k-diagnostic') for details.


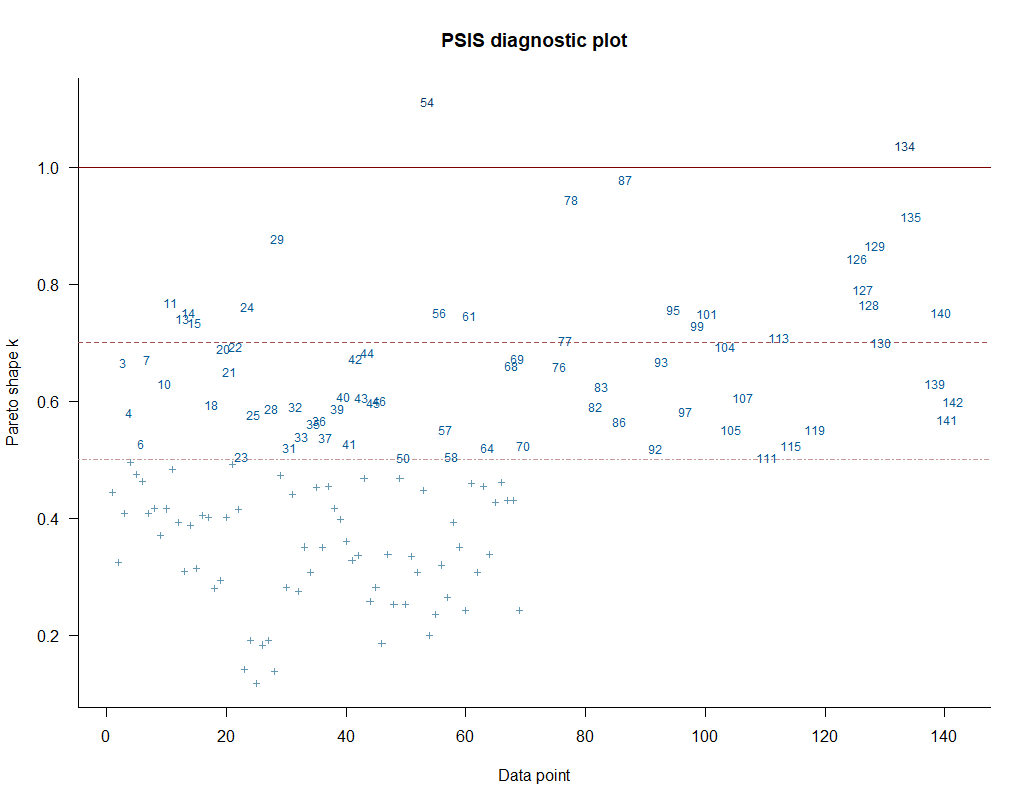


Figure 10 PSIS diagnostic plot of sulphonamide hierarchical Bayesian negative-binomial regression model

- 1. ***Tetracycline hierarchical Bayesian negative-binomial regression model:***

Output model:


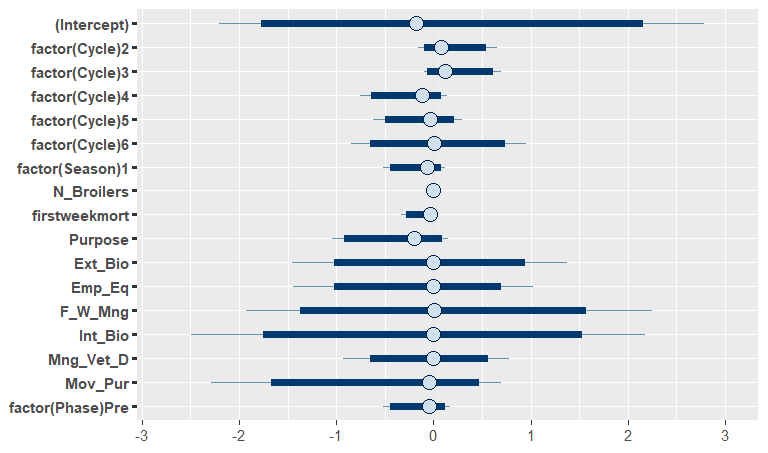


Figure 11 model output of tetracycline hierarchical Bayesian negative-binomial regression model

Model checks revealed a good fit:

> plot(y_loo, label_points = TRUE)

> y_loo

Computed from 8000 by 142 log-likelihood matrix

Estimate SE

elpd_loo -199.5 16.5

p_loo 28.9 5.8

looic 398.9 32.9

------

Monte Carlo SE of elpd_loo is NA.

Pareto k diagnostic values:

Count Pct. Min. n_eff

(-Inf, 0.5] (good) 105 73.9% 951

(0.5, 0.7] (ok) 33 23.2% 82

(0.7, 1] (bad) 3 2.1% 13

(1, Inf) (very bad) 1 0.7% 11

See help('pareto-k-diagnostic') for details.


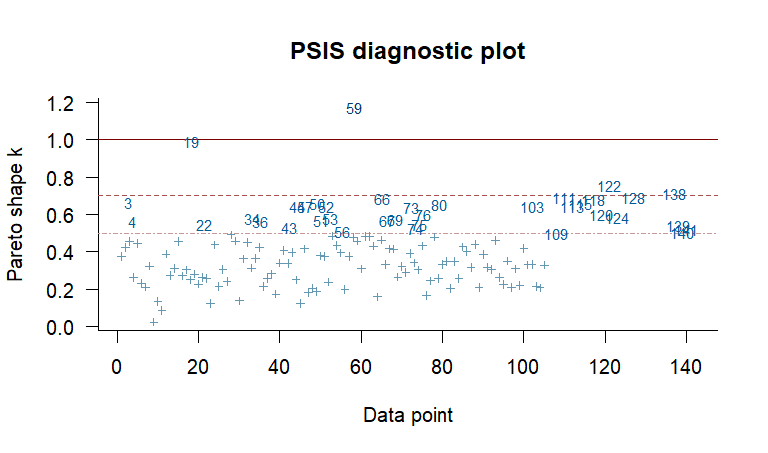


Figure 12 PSIS diagnostic plot of tetracycline hierarchical Bayesian negative-binomial regression model

To further illustrate the coefficient estimates, the distribution of the coefficient estimates from the model were plotted (Figure 5). The density plots provide a visual representation of the posterior distribution for each coefficient, including their central tendency and spread. Most variables show posterior distributions tightly centred around zero, with little evidence of a substantial effect on N_resistant.

**2.0 AMR model**

To evaluate the effect of the interventions on AMR, we analysed the mean number of tested antimicrobials (out of 14 tested) to which the commensal E. coli isolates from each farm exhibited NWT phenotypes (N_resistant). It is important to note that strains were considered and coded as “resistant” in the model, based on ECOFFs that define NWT phenotypes as described earlier. This outcome was analysed using a Bayesian Gaussian linear model. Since AMR data were collected only once before (Phase 1) and once after (Phase 3) intervention, this analysis was conducted at farm level, rather than by production cycle as was done for the AMU model. To ensure temporal alignment between AMU and AMR data, only AMU data from the three production cycles immediately preceding each AMR sampling were included, resulting in 57 production cycles from Phase 1 and 42 production cycles from Phase 3.

To ensure a good model fit, the number of antimicrobial treatment days was standardised (t_AMU). This was done by subtracting the overall mean from the total number of treatment days across three successive production cycles per farm and dividing by the standard deviation. This process produced a variable with a mean of 0 and a standard deviation of 1. The same method was applied to the number of broilers per farm (n_broilers). The overall mean (across all farms) was subtracted from the average number of broilers per study house per farm. The result was then divided by the standard deviation. This created a standardised variable with a mean of 0 and a standard deviation of 1, allowing for easier cross-farm comparisons.

Given our limited sample size and the inclusion of many predictor variables, not all of which are expected to have a large effect, the model employed regularized horseshoe priors using the R package rstanarm. This type of regularization prior is particularly effective in high-dimensional settings where many predictors may have minimal or no influence on the outcome. This prior adjusts the global shrinkage parameter (global_scale) based on the expected ratio of non-zero to zero coefficients, scaled by the square root of the number of observations [1].

The hierarchical shrinkage priors used in this model are characterized by very tall modes and heavy tails. This results in posterior distributions that are highly concentrated near zero unless a predictor has a strong influence on the outcome, in which case the prior exerts minimal influence, allowing the data to dominate the estimation. This approach effectively balances the need for regularization with the flexibility to allow important predictors to have substantial effects and is therefore considered preferred over other approaches, for studies with low sample sizes and many predictors with a limited expected effect [1].

The model output is provided in the manuscript. The distribution plots of all coefficient estimates are visible in the figure below:


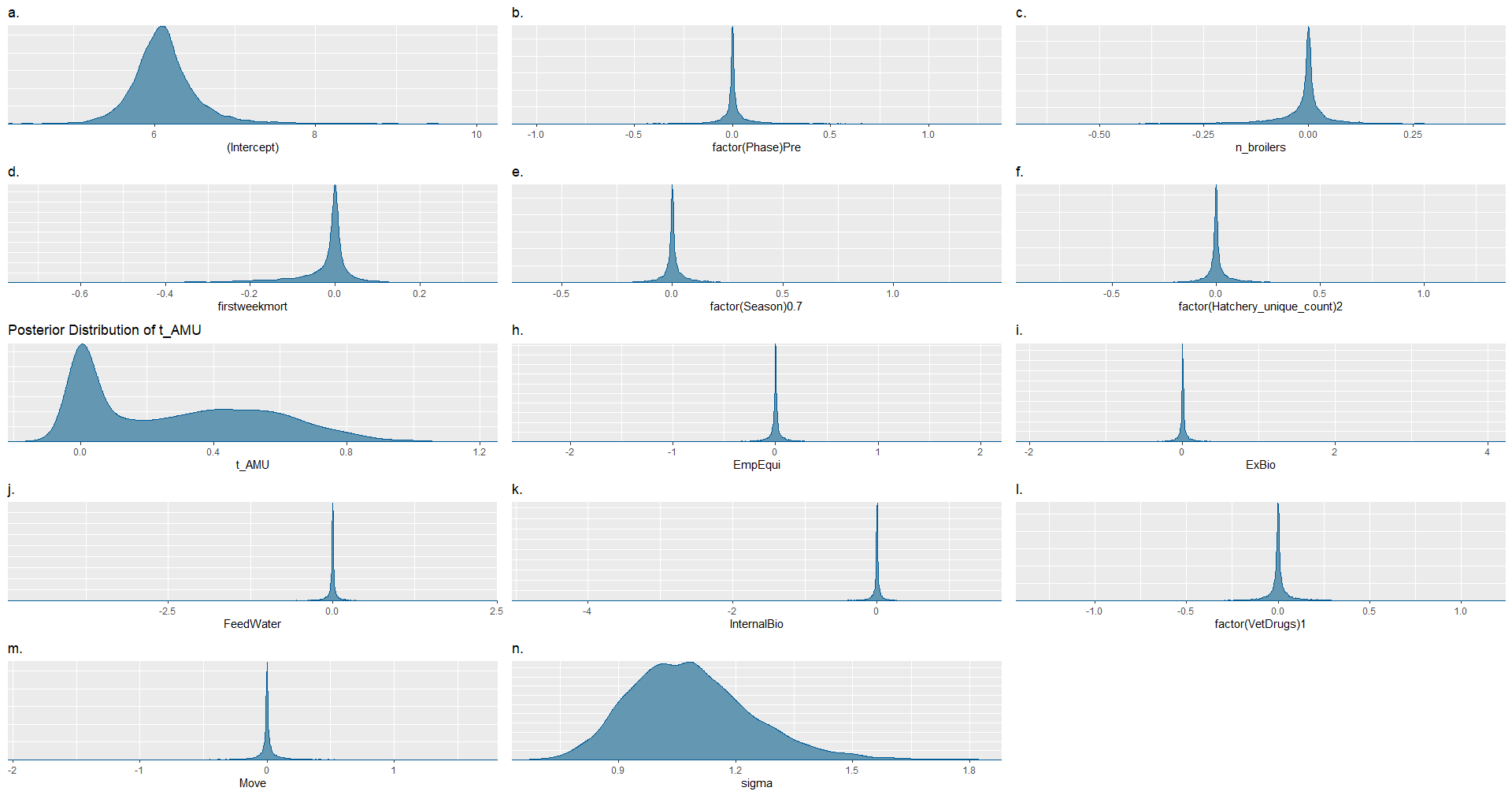


Figure 13 Distribution plots of the coefficient estimates of the independent variables included in the model

However, the parameter t_AMU (Figure 5, plot g) shows a posterior distribution with a rightward tail, indicating that higher values fall within the 95% credible interval. This extended tail suggests some evidence of a potential positive association between t_AMU and N_resistant, implying that an increase in the number of antimicrobial treatment days may be associated with an increase in the number of NWT phenotypes. Nonetheless, caution is warranted, as the credible interval includes zero.

The model fit was evaluated using three diagnostic methods.

First, **posterior predictive checks** were performed to assess the Bayesian Gaussian linear model's ability to capture the observed data patterns. Figure 6 shows the close alignment between the observed data and the range of posterior predictions, indicating that the model adequately captures the overall pattern and variability of N_resistant. This supports the validity of the model's predictive capacity.


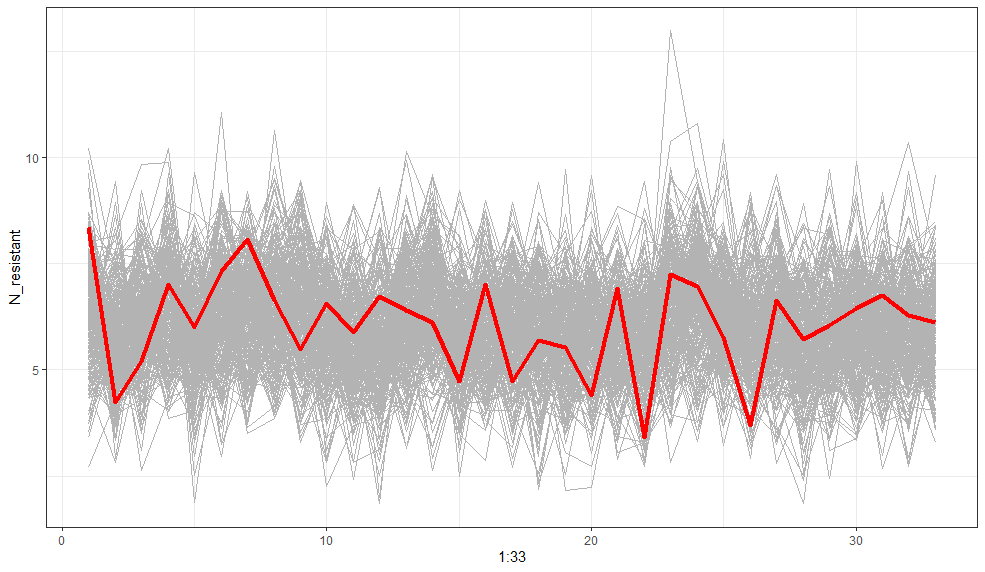


Figure 14 The grey lines represent the posterior predictive samples, while the red line indicates the observed data across 33 time points.

Next, **leave-one-out cross-validation (LOO)** with Pareto-smoothed importance sampling (PSIS) was conducted to assess predictive performance. The PSIS diagnostic plot (Figure 7) shows that 97% of data points had Pareto kkk values within the stable range (−∞, 0.5), and only one data point (3%) had a kkk value between 0.5 and 0.7, which is acceptable but warrants some caution. No data points exceeded the critical threshold of k>0.7k > 0.7k>0.7, confirming stable and reliable model fit. The model yielded an effective number of parameters (ploo) of 4.0 (SE = 1.0), an expected log pointwise predictive density (elpd_loo) of -52.3 (SE = 4.2), and a LOO information criterion (LOOIC) of 104.6 (SE = 8.5). The low Monte Carlo standard error for elpd_loo (0.0) further demonstrates high precision in the estimate.


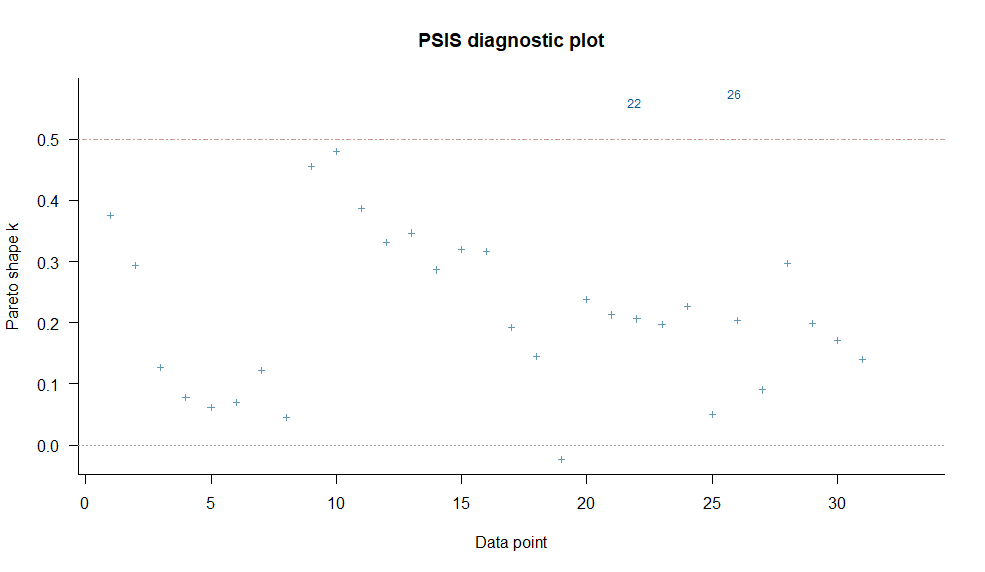


Figure 15 PSIS Diagnostic Plot for Model Fit Evaluation. This PSIS (Pareto Smoothed Importance Sampling) diagnostic plot displays the Pareto kk values for each data point in the model, used to assess the stability of the loo (leave-one-out cross-validation) estimates. Each point represents a data observation, with its corresponding Pareto kk value on the y-axis. Values below 0.5 generally indicate reliable estimates, while values above this threshold may suggest that certain observations exert a strong influence on the model’s predictive accuracy and could benefit from further investigation or model refinement. In this plot, all but two data points have kk values below 0.7, indicating an overall stable and reliable model fit.

Finally, a **second posterior predictive check** (Figure 8) compared the observed data distribution with the posterior predictive distribution. The observed density falls well within the predictive distribution, confirming that the model captures the general shape and spread of the observed data. Minor discrepancies suggest potential areas for further refinement if needed.


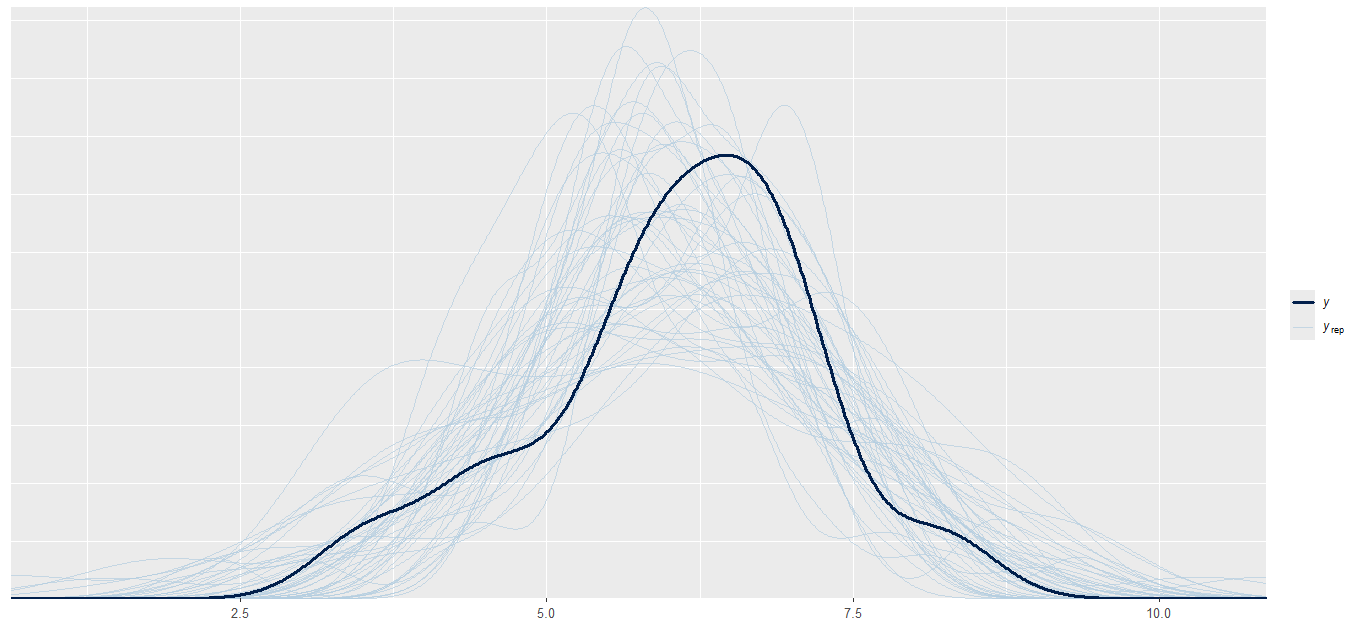


Figure 16 Posterior Predictive Check for the Bayesian Gaussian Linear Model

In summary, these diagnostics confirm that the model's assumptions are appropriate, and its predictions align well with the observed data, indicating reliable predictive performance.

**COMPLETE R SCRIPT**

#AMU model dataset

d_aggr<-read.csv("d_aggrtotal21012025.csv",header=T)

write_xlsx(d_aggr, "AMUmodel16425_data.xlsx")

#AMR model dataset

write.csv(D3,"d_perfarm30012025CORRECT.csv",row.names=F)

D3<-read.csv("d_perfarm30012025CORRECT.csv",header=T)

#libraries used

library(dplyr)

library(tidyr)

library(ggplot2)

library(bayesplot)

library(readxl)

library(rstanarm)

#AMU data analysis

#analysis AMU data

colnames(d_aggr)

table(d_aggr$POL_count,useNA="ifany")

table(d_aggr$FLU_count,useNA="ifany")

table(d_aggr$MAC_count,useNA="ifany")

table(d_aggr$FOS_count,useNA="ifany")

table(d_aggr$AMI_count,useNA="ifany")

table(d_aggr$PEN_count,useNA="ifany")

table(d_aggr$SUL_count,useNA="ifany")

table(d_aggr$LIN_count,useNA="ifany")

table(d_aggr$TET_count,useNA="ifany")

table(d_aggr$TRI_count,useNA="ifany")

table(d_aggr$AMI_IA_count,useNA="ifany")

#models per antibiotic except for FOS, AMI, LIN, AMI, TRI, use too limited

#T_AMU

#total AMU model

fit.amu <- stan_glmer(

T_AMU ~ factor(Cycle) + factor(Season) + N_Broilers +

firstweekmort + Ext_Bio + Emp_Eq + Purpose +

F_W_Mng + Int_Bio + Mng_Vet_D + Mov_Pur + factor(Phase) + Purpose +

(1 | Farm_n/Hatchery_n)

data = d_aggr,

family = neg_binomial_2(),

chains = 4,

iter = 4000,

adapt_delta = 0.99,

prior = lasso(df = 1, location = 0, scale = NULL, autoscale = TRUE)

)

# Plot only the specified parameters

param <- names(fit.amu$coefficients[1:17])

plot(fit.amu, pars = param, prob = 0.9, prob_outer = 0.95)+theme_minimal()

plot(fit.amu,prob=.9,prob_outer=.95)

summary(fit.amu,probs=c(0.025,0.975))

pp_check(fit.amu)

y_loo <- loo(fit.amu)

plot(y_loo, label_points = TRUE)

mcmc_dens(fit.amu, pars = "factor(Phase)Pre") + ggtitle("Total AMU")

#Polymyxin

fit.pol <- stan_glmer(

POL_count ~ factor(Cycle) + factor(Season) + N_Broilers +

firstweekmort + Ext_Bio + Emp_Eq + Purpose +

F_W_Mng + Int_Bio + Mng_Vet_D + Mov_Pur + factor(Phase) + Purpose +

(1 | Farm_n / Hatchery_n),

data = d_aggr,

family = neg_binomial_2(), # Specify the negative binomial family

chains = 4,

iter = 4000,

adapt_delta = 0.99,

prior = lasso(df = 1, location = 0, scale = NULL, autoscale = TRUE)

)

# Plot only the specified parameters

param <- names(fit.pol$coefficients[1:17])

plot(fit.pol, pars = param, prob = 0.9, prob_outer = 0.95)+theme_minimal()

summary(fit.pol,probs=c(0.025,0.975))

pp_check(fit.pol)

y_loo <- loo(fit.pol)

plot(y_loo, label_points = TRUE)

mcmc_dens(fit.pen, pars = "factor(Phase)Pre") + ggtitle("Total AMU Penicillin")

#Fluoroquinolone

fit.flu <- stan_glmer(

FLU_count ~ factor(Cycle) + factor(Season) + N_Broilers +

firstweekmort + Ext_Bio + Emp_Eq +

F_W_Mng + Int_Bio + Mng_Vet_D + Mov_Pur + factor(Phase) +

(1 | Farm_n / Hatchery_n),

data = d_aggr,

family = neg_binomial_2(), # Specify the negative binomial family

chains = 4,

iter = 4000,

adapt_delta = 0.99,

prior = lasso(df = 1, location = 0, scale = NULL, autoscale = TRUE)

)

param <- names(fit.flu$coefficients[1:17])

plot(fit.flu,pars=param,prob=.9,prob_outer=.95) #pars is kan je aangeven welke je wilt laten zien (even checken)

summary(fit.flu,probs=c(0.025,0.975))

pp_check(fit.flu)

y_loo <- loo(fit.flu)

plot(y_loo, label_points = TRUE)

#Macrolide

fit.mac <- stan_glmer(

MAC_count ~ factor(Cycle) + factor(Season) + N_Broilers +

firstweekmort + Purpose + Ext_Bio + Emp_Eq +

F_W_Mng + Int_Bio + Mng_Vet_D + Mov_Pur + factor(Phase) +

(1 | Farm_n/Hatchery_n),

data = d_aggr,

family = neg_binomial_2(), # Specify the negative binomial family

chains = 4,

iter = 4000,

adapt_delta = 0.99,

prior = lasso(df = 1, location = 0, scale = NULL, autoscale = TRUE)

)

param <- names(fit.mac$coefficients[1:17])

plot(fit.mac,pars=param,prob=.9,prob_outer=.95)

summary(fit.mac,probs=c(0.025,0.975))

pp_check(fit.mac)

y_loo <- loo(fit.mac)

plot(y_loo, label_points = TRUE)

#Penicillin

fit.pen <- stan_glmer(

PEN_count ~ factor(Cycle) + factor(Season) + N_Broilers +

firstweekmort + Purpose + Ext_Bio + Emp_Eq +

F_W_Mng + Int_Bio + Mng_Vet_D + Mov_Pur + factor(Phase) +

(1 | Farm_n/Hatchery_n),

data = d_aggr,

family = neg_binomial_2(), # Specify the negative binomial family

chains = 4,

iter = 4000,

adapt_delta = 0.99,

prior = lasso(df = 1, location = 0, scale = NULL, autoscale = TRUE)

)

param <- names(fit.pen$coefficients[1:17])

plot(fit.pen,pars=param,prob=.9,prob_outer=.95)

summary(fit.pen,probs=c(0.025,0.975))

pp_check(fit.pen)

y_loo <- loo(fit.pen)

plot(y_loo, label_points = TRUE)

#Sulfonamide

fit.sul <- stan_glmer(

SUL_count ~ factor(Cycle) + factor(Season) + N_Broilers +

firstweekmort + Purpose + Ext_Bio + Emp_Eq +

F_W_Mng + Int_Bio + Mng_Vet_D + Mov_Pur + factor(Phase) +

(1 | Farm_n/Hatchery_n),

data = d_aggr,

family = neg_binomial_2(), # Specify the negative binomial family

chains = 4,

iter = 4000,

adapt_delta = 0.99,

prior = lasso(df = 1, location = 0, scale = NULL, autoscale = TRUE)

)

param <- names(fit.sul$coefficients[1:17])

plot(fit.sul,pars=param,prob=.9,prob_outer=.95)

summary(fit.sul,probs=c(0.025,0.975))

pp_check(fit.sul)

y_loo <- loo(fit.sul)

plot(y_loo, label_points = TRUE)

#Tetracycline

unique(d_aggr$Farm_ID)

fit.tet <- stan_glmer(

TET_count ~ factor(Cycle) + factor(Season) + N_Broilers +

firstweekmort + Purpose + Ext_Bio + Emp_Eq +

F_W_Mng + Int_Bio + Mng_Vet_D + Mov_Pur + factor(Phase) +

(1 | Farm_n/Hatchery_n),

data = d_aggr,

family = neg_binomial_2(), # Specify the negative binomial family

chains = 4,

iter = 4000,

adapt_delta = 0.99,

prior = lasso(df = 1, location = 0, scale = NULL, autoscale = TRUE)

)

plot(fit.tet,prob=.9,prob_outer=.95)

summary(fit.tet,probs=c(0.025,0.975))

#-----------------------------------

#AMR modeling

D3$Season <- round(D3$Season,digits = 1)

D3$Season[D3$Season==0] <- 0.3

D3$Season[D3$Season==1] <- 0.7

D3$Brand[D3$Brand==0] <- 1

count_columns <- grep("count", colnames(D3), value = TRUE)

F <- stan_glm(N_resistant~ factor(Phase)+n_broilers+

firstweekmort+

factor(Season)+factor(Hatchery_unique_count)+t_AMU+

EmpEqui+ExBio+FeedWater+InternalBio+

VetDrugs+Move,

family=gaussian,

prior=hs(df = 1, global_df = 1, global_scale = 0.025,

slab_df = 4, slab_scale = 2.5),

#prior=laplace(location = 0, scale = NULL,

# autoscale = TRUE),

data = D3,chains=4,iter=4000,adapt_delta=0.9999)

##

y_postl <- as.data.frame(t(posterior_predict(F)))

p <- ggplot(D3,aes(x=1:33,y=N_resistant))+theme_bw()

for (i in 1:300){

p <- p+geom_line(data=y_postl,aes_string(x=1:33,y=y_postl[,i]),

color="gray70")

}

p <- p+geom_line(data=D3,aes(x=1:33,y=N_resistant),

color="red",lwd=1.5)

p

##

##

y_loo <- loo(F)

plot(y_loo, label_points = TRUE)

y_loo

##

pp_check(F)

##

##

##==================== 90 en 50 % credibility intervals en checks

##

summary(F,probs=c(.025,.975),digits=2)

##

plot(F,prob=.9,prob_outer=.95)

##

# Load required libraries

library(gridExtra)

library(cowplot) # For adding labels to grid

# Create density plots for each variable

p1 <- mcmc_dens(F, pars = "(Intercept)") + ggtitle("a.")

p2 <- mcmc_dens(F, pars = "factor(Phase)Pre") + ggtitle("b.")

p3 <- mcmc_dens(F, pars = "n_broilers") + ggtitle("c.")

p4 <- mcmc_dens(F, pars = "firstweekmort") + ggtitle("d.")

p5 <- mcmc_dens(F, pars = "factor(Season)0.7") + ggtitle("e.")

p6 <- mcmc_dens(F, pars = "factor(Hatchery_unique_count)2") + ggtitle("f.")

p7 <- mcmc_dens(F, pars = "t_AMU") + ggtitle("Posterior Distribution of t_AMU")

p8 <- mcmc_dens(F, pars = "EmpEqui") + ggtitle("h.")

p9 <- mcmc_dens(F, pars = "ExBio") + ggtitle("i.")

p10 <- mcmc_dens(F, pars = "FeedWater") + ggtitle("j.")

p11 <- mcmc_dens(F, pars = "InternalBio") + ggtitle("k.")

p12 <- mcmc_dens(F, pars = "factor(VetDrugs)1") + ggtitle("l.")

p13 <- mcmc_dens(F, pars = "Move") + ggtitle("m.")

p14 <- mcmc_dens(F, pars = "sigma") + ggtitle("n.")

# Arrange all plots in a grid layout with labels

plot_grid(p1, p2, p3, p4, p5, p6, p7, p8, p9, p10, p11, p12, p13, p14, ncol = 3)

SUMMARY OF ALL MODELS

# ***Total AMU hierarchical Bayesian negative-binomial regression model***

Relevant for model fit: the Monte Carlo Standard Error (MCSE) for each parameter was small, indicating high precision in the posterior mean estimates. The potential scale reduction factor (Rhat) values were close to 1.00, suggesting that the model had likely converged effectively. Additionally, the effective sample size (n_eff) was sufficiently large, reflecting a high number of independent samples and providing confidence in the reliability of the parameter estimates.

Model Info:

function: stan_glmer

family: neg_binomial_2 [log]

formula: T_AMU ~ factor(Cycle) + factor(Season) + N_Broilers + firstweekmort +

Ext_Bio + Emp_Eq + Purpose + F_W_Mng + Int_Bio + Mng_Vet_D +

Mov_Pur + factor(Phase) + Purpose + (1 | Farm_n/Hatchery_n)

algorithm: sampling

sample: 8000 (posterior sample size)

priors: see help('prior_summary')

observations: 142

groups: Hatchery_n:Farm_n (95), Farm_n (19)

Estimates:

mean sd 2.5% 97.5%

(Intercept) 2.3 0.2 1.7 2.8

factor(Cycle)2 0.0 0.0 -0.1 0.1

factor(Cycle)3 0.0 0.0 -0.1 0.1

factor(Cycle)4 0.0 0.1 -0.1 0.1

factor(Cycle)5 0.0 0.1 -0.2 0.1

factor(Cycle)6 0.0 0.1 -0.3 0.3

factor(Season)1 0.0 0.0 -0.1 0.1

N_Broilers 0.0 0.0 0.0 0.0

firstweekmort 0.0 0.0 -0.1 0.0

Ext_Bio 0.1 0.3 -0.1 1.1

Emp_Eq 0.0 0.1 -0.3 0.2

Purpose 0.0 0.1 -0.3 0.1

F_W_Mng 0.0 0.2 -0.3 0.6

Int_Bio 0.0 0.2 -0.5 0.4

Mng_Vet_D 0.0 0.1 -0.1 0.3

Mov_Pur 0.0 0.1 -0.4 0.2

factor(Phase)Pre 0.0 0.1 0.0 0.3

b[(Intercept) Hatchery_n:Farm_n:Hatchery_1:Farm_18] 0.0 0.2 -0.3 0.4

b[(Intercept) Hatchery_n:Farm_n:Hatchery_1:Farm_19] 0.0 0.2 -0.4 0.4

b[(Intercept) Hatchery_n:Farm_n:Hatchery_10:Farm_1] -0.1 0.2 -0.5 0.3

b[(Intercept) Hatchery_n:Farm_n:Hatchery_10:Farm_2] -0.1 0.2 -0.6 0.3

b[(Intercept) Hatchery_n:Farm_n:Hatchery_11:Farm_3] 0.0 0.2 -0.4 0.5

b[(Intercept) Hatchery_n:Farm_n:Hatchery_12:Farm_10] 0.0 0.2 -0.4 0.4

b[(Intercept) Hatchery_n:Farm_n:Hatchery_13:Farm_13] 0.0 0.2 -0.3 0.5

b[(Intercept) Hatchery_n:Farm_n:Hatchery_14:Farm_11] 0.0 0.2 -0.4 0.4

b[(Intercept) Hatchery_n:Farm_n:Hatchery_15:Farm_13] 0.0 0.2 -0.4 0.4

b[(Intercept) Hatchery_n:Farm_n:Hatchery_16:Farm_14] 0.0 0.2 -0.3 0.5

b[(Intercept) Hatchery_n:Farm_n:Hatchery_17:Farm_15] 0.0 0.2 -0.3 0.4

b[(Intercept) Hatchery_n:Farm_n:Hatchery_18:Farm_3] 0.0 0.2 -0.3 0.5

b[(Intercept) Hatchery_n:Farm_n:Hatchery_18:Farm_4] 0.1 0.2 -0.2 0.5

b[(Intercept) Hatchery_n:Farm_n:Hatchery_19:Farm_3] 0.1 0.2 -0.3 0.5

b[(Intercept) Hatchery_n:Farm_n:Hatchery_2:Farm_15] 0.1 0.2 -0.3 0.5

b[(Intercept) Hatchery_n:Farm_n:Hatchery_2:Farm_18] -0.1 0.2 -0.5 0.4

b[(Intercept) Hatchery_n:Farm_n:Hatchery_2:Farm_7] -0.1 0.2 -0.6 0.3

b[(Intercept) Hatchery_n:Farm_n:Hatchery_2:Farm_8] 0.1 0.2 -0.3 0.5

b[(Intercept) Hatchery_n:Farm_n:Hatchery_2:Farm_9] 0.0 0.2 -0.4 0.5

b[(Intercept) Hatchery_n:Farm_n:Hatchery_20:Farm_12] 0.0 0.2 -0.5 0.4

b[(Intercept) Hatchery_n:Farm_n:Hatchery_21:Farm_4] -0.1 0.2 -0.6 0.3

b[(Intercept) Hatchery_n:Farm_n:Hatchery_22:Farm_14] 0.1 0.2 -0.2 0.5

b[(Intercept) Hatchery_n:Farm_n:Hatchery_22:Farm_15] 0.0 0.2 -0.4 0.4

b[(Intercept) Hatchery_n:Farm_n:Hatchery_22:Farm_2] 0.1 0.2 -0.3 0.6

b[(Intercept) Hatchery_n:Farm_n:Hatchery_22:Farm_5] 0.0 0.2 -0.4 0.4

b[(Intercept) Hatchery_n:Farm_n:Hatchery_22:Farm_6] -0.1 0.2 -0.5 0.3

b[(Intercept) Hatchery_n:Farm_n:Hatchery_23:Farm_5] 0.1 0.2 -0.3 0.5

b[(Intercept) Hatchery_n:Farm_n:Hatchery_24:Farm_6] -0.1 0.2 -0.7 0.3

b[(Intercept) Hatchery_n:Farm_n:Hatchery_25:Farm_6] 0.3 0.2 -0.1 0.8

b[(Intercept) Hatchery_n:Farm_n:Hatchery_26:Farm_5] 0.1 0.2 -0.3 0.5

b[(Intercept) Hatchery_n:Farm_n:Hatchery_27:Farm_5] 0.0 0.2 -0.3 0.5

b[(Intercept) Hatchery_n:Farm_n:Hatchery_28:Farm_13] 0.0 0.2 -0.4 0.4

b[(Intercept) Hatchery_n:Farm_n:Hatchery_29:Farm_12] -0.1 0.2 -0.5 0.3

b[(Intercept) Hatchery_n:Farm_n:Hatchery_29:Farm_13] 0.0 0.2 -0.4 0.4

b[(Intercept) Hatchery_n:Farm_n:Hatchery_29:Farm_6] 0.0 0.2 -0.4 0.4

b[(Intercept) Hatchery_n:Farm_n:Hatchery_3:Farm_8] -0.1 0.2 -0.6 0.2

b[(Intercept) Hatchery_n:Farm_n:Hatchery_30:Farm_13] -0.1 0.2 -0.6 0.3

b[(Intercept) Hatchery_n:Farm_n:Hatchery_31:Farm_10] 0.0 0.2 -0.5 0.4

b[(Intercept) Hatchery_n:Farm_n:Hatchery_31:Farm_11] 0.0 0.2 -0.5 0.4

b[(Intercept) Hatchery_n:Farm_n:Hatchery_31:Farm_15] 0.0 0.2 -0.4 0.4

b[(Intercept) Hatchery_n:Farm_n:Hatchery_32:Farm_8] -0.2 0.2 -0.8 0.2

b[(Intercept) Hatchery_n:Farm_n:Hatchery_33:Farm_13] 0.0 0.2 -0.5 0.4

b[(Intercept) Hatchery_n:Farm_n:Hatchery_34:Farm_7] 0.0 0.2 -0.4 0.4

b[(Intercept) Hatchery_n:Farm_n:Hatchery_34:Farm_8] 0.1 0.2 -0.2 0.7

b[(Intercept) Hatchery_n:Farm_n:Hatchery_35:Farm_9] 0.0 0.2 -0.4 0.5

b[(Intercept) Hatchery_n:Farm_n:Hatchery_36:Farm_12] 0.0 0.2 -0.5 0.4

b[(Intercept) Hatchery_n:Farm_n:Hatchery_37:Farm_19] 0.0 0.2 -0.5 0.4

b[(Intercept) Hatchery_n:Farm_n:Hatchery_38:Farm_14] 0.1 0.2 -0.3 0.5

b[(Intercept) Hatchery_n:Farm_n:Hatchery_39:Farm_2] 0.0 0.2 -0.4 0.5

b[(Intercept) Hatchery_n:Farm_n:Hatchery_40:Farm_1] 0.0 0.2 -0.3 0.5

b[(Intercept) Hatchery_n:Farm_n:Hatchery_40:Farm_10] -0.1 0.2 -0.4 0.3

b[(Intercept) Hatchery_n:Farm_n:Hatchery_40:Farm_11] 0.0 0.2 -0.4 0.3

b[(Intercept) Hatchery_n:Farm_n:Hatchery_40:Farm_16] 0.0 0.2 -0.4 0.3

b[(Intercept) Hatchery_n:Farm_n:Hatchery_40:Farm_17] -0.1 0.2 -0.6 0.2

b[(Intercept) Hatchery_n:Farm_n:Hatchery_40:Farm_9] 0.0 0.2 -0.4 0.5

b[(Intercept) Hatchery_n:Farm_n:Hatchery_41:Farm_16] -0.1 0.2 -0.6 0.3

b[(Intercept) Hatchery_n:Farm_n:Hatchery_42:Farm_11] 0.0 0.2 -0.4 0.4

b[(Intercept) Hatchery_n:Farm_n:Hatchery_43:Farm_11] 0.0 0.2 -0.4 0.4

b[(Intercept) Hatchery_n:Farm_n:Hatchery_45:Farm_2] -0.1 0.2 -0.7 0.2

b[(Intercept) Hatchery_n:Farm_n:Hatchery_46:Farm_19] 0.2 0.2 -0.1 0.8

b[(Intercept) Hatchery_n:Farm_n:Hatchery_47:Farm_18] 0.0 0.2 -0.5 0.4

b[(Intercept) Hatchery_n:Farm_n:Hatchery_48:Farm_18] 0.0 0.2 -0.4 0.4

b[(Intercept) Hatchery_n:Farm_n:Hatchery_49:Farm_16] -0.1 0.2 -0.7 0.3

b[(Intercept) Hatchery_n:Farm_n:Hatchery_5:Farm_18] 0.0 0.2 -0.4 0.4

b[(Intercept) Hatchery_n:Farm_n:Hatchery_5:Farm_19] 0.0 0.2 -0.4 0.4

b[(Intercept) Hatchery_n:Farm_n:Hatchery_50:Farm_4] 0.1 0.2 -0.2 0.6

b[(Intercept) Hatchery_n:Farm_n:Hatchery_51:Farm_14] 0.1 0.2 -0.3 0.5

b[(Intercept) Hatchery_n:Farm_n:Hatchery_51:Farm_18] 0.1 0.2 -0.3 0.6

b[(Intercept) Hatchery_n:Farm_n:Hatchery_52:Farm_17] -0.1 0.2 -0.5 0.3

b[(Intercept) Hatchery_n:Farm_n:Hatchery_53:Farm_19] -0.2 0.3 -0.8 0.1

b[(Intercept) Hatchery_n:Farm_n:Hatchery_54:Farm_14] -0.1 0.2 -0.7 0.3

b[(Intercept) Hatchery_n:Farm_n:Hatchery_55:Farm_16] 0.1 0.2 -0.3 0.5

b[(Intercept) Hatchery_n:Farm_n:Hatchery_56:Farm_10] 0.0 0.2 -0.4 0.4

b[(Intercept) Hatchery_n:Farm_n:Hatchery_57:Farm_19] -0.1 0.2 -0.6 0.3

b[(Intercept) Hatchery_n:Farm_n:Hatchery_58:Farm_3] 0.0 0.2 -0.4 0.4

b[(Intercept) Hatchery_n:Farm_n:Hatchery_59:Farm_16] 0.1 0.2 -0.3 0.6

b[(Intercept) Hatchery_n:Farm_n:Hatchery_59:Farm_17] 0.0 0.2 -0.4 0.4

b[(Intercept) Hatchery_n:Farm_n:Hatchery_6:Farm_4] -0.1 0.2 -0.6 0.3

b[(Intercept) Hatchery_n:Farm_n:Hatchery_60:Farm_17] -0.1 0.2 -0.6 0.3

b[(Intercept) Hatchery_n:Farm_n:Hatchery_60:Farm_9] 0.0 0.2 -0.5 0.4

b[(Intercept) Hatchery_n:Farm_n:Hatchery_61:Farm_5] 0.0 0.2 -0.3 0.5

b[(Intercept) Hatchery_n:Farm_n:Hatchery_62:Farm_17] 0.2 0.2 -0.1 0.8

b[(Intercept) Hatchery_n:Farm_n:Hatchery_63:Farm_1] 0.1 0.2 -0.2 0.7

b[(Intercept) Hatchery_n:Farm_n:Hatchery_63:Farm_4] 0.1 0.2 -0.3 0.5

b[(Intercept) Hatchery_n:Farm_n:Hatchery_64:Farm_5] -0.1 0.2 -0.6 0.3

b[(Intercept) Hatchery_n:Farm_n:Hatchery_64:Farm_6] 0.0 0.2 -0.4 0.4

b[(Intercept) Hatchery_n:Farm_n:Hatchery_65:Farm_14] 0.0 0.2 -0.3 0.5

b[(Intercept) Hatchery_n:Farm_n:Hatchery_66:Farm_2] 0.0 0.2 -0.4 0.5

b[(Intercept) Hatchery_n:Farm_n:Hatchery_67:Farm_12] 0.1 0.2 -0.2 0.6

b[(Intercept) Hatchery_n:Farm_n:Hatchery_67:Farm_9] 0.1 0.2 -0.3 0.5

b[(Intercept) Hatchery_n:Farm_n:Hatchery_68:Farm_12] -0.1 0.2 -0.6 0.2

b[(Intercept) Hatchery_n:Farm_n:Hatchery_69:Farm_12] -0.1 0.2 -0.6 0.4

b[(Intercept) Hatchery_n:Farm_n:Hatchery_7:Farm_13] 0.0 0.2 -0.5 0.4

b[(Intercept) Hatchery_n:Farm_n:Hatchery_8:Farm_7] -0.1 0.2 -0.6 0.3

b[(Intercept) Hatchery_n:Farm_n:Hatchery_9:Farm_19] 0.1 0.2 -0.3 0.5

b[(Intercept) Farm_n:Farm_1] 0.4 0.2 -0.1 0.8

b[(Intercept) Farm_n:Farm_10] -0.2 0.2 -0.7 0.2

b[(Intercept) Farm_n:Farm_11] -0.2 0.2 -0.6 0.2

b[(Intercept) Farm_n:Farm_12] -0.5 0.2 -0.9 0.0

b[(Intercept) Farm_n:Farm_13] -0.3 0.2 -0.7 0.1

b[(Intercept) Farm_n:Farm_14] 0.5 0.2 0.0 0.9

b[(Intercept) Farm_n:Farm_15] 0.2 0.2 -0.2 0.6

b[(Intercept) Farm_n:Farm_16] -0.1 0.2 -0.5 0.3

b[(Intercept) Farm_n:Farm_17] -0.1 0.2 -0.5 0.3

b[(Intercept) Farm_n:Farm_18] 0.1 0.2 -0.3 0.5

b[(Intercept) Farm_n:Farm_19] -0.2 0.2 -0.6 0.2

b[(Intercept) Farm_n:Farm_2] -0.2 0.2 -0.7 0.2

b[(Intercept) Farm_n:Farm_3] 0.4 0.2 -0.1 0.8

b[(Intercept) Farm_n:Farm_4] 0.1 0.2 -0.3 0.5

b[(Intercept) Farm_n:Farm_5] 0.3 0.2 -0.1 0.7

b[(Intercept) Farm_n:Farm_6] 0.2 0.2 -0.2 0.7

b[(Intercept) Farm_n:Farm_7] -0.4 0.3 -1.0 0.1

b[(Intercept) Farm_n:Farm_8] -0.2 0.2 -0.7 0.2

b[(Intercept) Farm_n:Farm_9] 0.3 0.2 -0.1 0.8

reciprocal_dispersion 6.6 1.6 4.0 10.3

Sigma[Hatchery_n:Farm_n:(Intercept),(Intercept)] 0.1 0.0 0.0 0.2

Sigma[Farm_n:(Intercept),(Intercept)] 0.2 0.1 0.0 0.4

Fit Diagnostics:

mean sd 2.5% 97.5%

mean_PPD 11.0 0.7 9.6 12.4

The mean_ppd is the sample average posterior predictive distribution of the outcome variable (for details see help('summary.stanreg')).

MCMC diagnostics

mcse Rhat n_eff

(Intercept) 0.0 1.0 5664

factor(Cycle)2 0.0 1.0 9166

factor(Cycle)3 0.0 1.0 6919

factor(Cycle)4 0.0 1.0 8070

factor(Cycle)5 0.0 1.0 3592

factor(Cycle)6 0.0 1.0 8308

factor(Season)1 0.0 1.0 8650

N_Broilers 0.0 1.0 6242

firstweekmort 0.0 1.0 2731

Ext_Bio 0.0 1.0 1575

Emp_Eq 0.0 1.0 6988

Purpose 0.0 1.0 3663

F_W_Mng 0.0 1.0 5009

Int_Bio 0.0 1.0 7963

Mng_Vet_D 0.0 1.0 5715

Mov_Pur 0.0 1.0 4151

factor(Phase)Pre 0.0 1.0 2140

b[(Intercept) Hatchery_n:Farm_n:Hatchery_1:Farm_18] 0.0 1.0 8418

b[(Intercept) Hatchery_n:Farm_n:Hatchery_1:Farm_19] 0.0 1.0 11744

b[(Intercept) Hatchery_n:Farm_n:Hatchery_10:Farm_1] 0.0 1.0 9561

b[(Intercept) Hatchery_n:Farm_n:Hatchery_10:Farm_2] 0.0 1.0 7094

b[(Intercept) Hatchery_n:Farm_n:Hatchery_11:Farm_3] 0.0 1.0 9347

b[(Intercept) Hatchery_n:Farm_n:Hatchery_12:Farm_10] 0.0 1.0 10731

b[(Intercept) Hatchery_n:Farm_n:Hatchery_13:Farm_13] 0.0 1.0 10850

b[(Intercept) Hatchery_n:Farm_n:Hatchery_14:Farm_11] 0.0 1.0 10887

b[(Intercept) Hatchery_n:Farm_n:Hatchery_15:Farm_13] 0.0 1.0 9995

b[(Intercept) Hatchery_n:Farm_n:Hatchery_16:Farm_14] 0.0 1.0 8673

b[(Intercept) Hatchery_n:Farm_n:Hatchery_17:Farm_15] 0.0 1.0 9387

b[(Intercept) Hatchery_n:Farm_n:Hatchery_18:Farm_3] 0.0 1.0 8572

b[(Intercept) Hatchery_n:Farm_n:Hatchery_18:Farm_4] 0.0 1.0 7121

b[(Intercept) Hatchery_n:Farm_n:Hatchery_19:Farm_3] 0.0 1.0 6948

b[(Intercept) Hatchery_n:Farm_n:Hatchery_2:Farm_15] 0.0 1.0 8202

b[(Intercept) Hatchery_n:Farm_n:Hatchery_2:Farm_18] 0.0 1.0 8731

b[(Intercept) Hatchery_n:Farm_n:Hatchery_2:Farm_7] 0.0 1.0 6007

b[(Intercept) Hatchery_n:Farm_n:Hatchery_2:Farm_8] 0.0 1.0 6655

b[(Intercept) Hatchery_n:Farm_n:Hatchery_2:Farm_9] 0.0 1.0 10296

b[(Intercept) Hatchery_n:Farm_n:Hatchery_20:Farm_12] 0.0 1.0 9692

b[(Intercept) Hatchery_n:Farm_n:Hatchery_21:Farm_4] 0.0 1.0 5889

b[(Intercept) Hatchery_n:Farm_n:Hatchery_22:Farm_14] 0.0 1.0 4485

b[(Intercept) Hatchery_n:Farm_n:Hatchery_22:Farm_15] 0.0 1.0 9865

b[(Intercept) Hatchery_n:Farm_n:Hatchery_22:Farm_2] 0.0 1.0 5705

b[(Intercept) Hatchery_n:Farm_n:Hatchery_22:Farm_5] 0.0 1.0 11723

b[(Intercept) Hatchery_n:Farm_n:Hatchery_22:Farm_6] 0.0 1.0 10780

b[(Intercept) Hatchery_n:Farm_n:Hatchery_23:Farm_5] 0.0 1.0 6675

b[(Intercept) Hatchery_n:Farm_n:Hatchery_24:Farm_6] 0.0 1.0 5184

b[(Intercept) Hatchery_n:Farm_n:Hatchery_25:Farm_6] 0.0 1.0 2077

b[(Intercept) Hatchery_n:Farm_n:Hatchery_26:Farm_5] 0.0 1.0 6164

b[(Intercept) Hatchery_n:Farm_n:Hatchery_27:Farm_5] 0.0 1.0 8366

b[(Intercept) Hatchery_n:Farm_n:Hatchery_28:Farm_13] 0.0 1.0 10449

b[(Intercept) Hatchery_n:Farm_n:Hatchery_29:Farm_12] 0.0 1.0 7171

b[(Intercept) Hatchery_n:Farm_n:Hatchery_29:Farm_13] 0.0 1.0 10820

b[(Intercept) Hatchery_n:Farm_n:Hatchery_29:Farm_6] 0.0 1.0 10147

b[(Intercept) Hatchery_n:Farm_n:Hatchery_3:Farm_8] 0.0 1.0 5351

b[(Intercept) Hatchery_n:Farm_n:Hatchery_30:Farm_13] 0.0 1.0 5696

b[(Intercept) Hatchery_n:Farm_n:Hatchery_31:Farm_10] 0.0 1.0 9807

b[(Intercept) Hatchery_n:Farm_n:Hatchery_31:Farm_11] 0.0 1.0 9473

b[(Intercept) Hatchery_n:Farm_n:Hatchery_31:Farm_15] 0.0 1.0 10708

b[(Intercept) Hatchery_n:Farm_n:Hatchery_32:Farm_8] 0.0 1.0 3106

b[(Intercept) Hatchery_n:Farm_n:Hatchery_33:Farm_13] 0.0 1.0 8767

b[(Intercept) Hatchery_n:Farm_n:Hatchery_34:Farm_7] 0.0 1.0 10414

b[(Intercept) Hatchery_n:Farm_n:Hatchery_34:Farm_8] 0.0 1.0 4479

b[(Intercept) Hatchery_n:Farm_n:Hatchery_35:Farm_9] 0.0 1.0 9786

b[(Intercept) Hatchery_n:Farm_n:Hatchery_36:Farm_12] 0.0 1.0 8694

b[(Intercept) Hatchery_n:Farm_n:Hatchery_37:Farm_19] 0.0 1.0 9244

b[(Intercept) Hatchery_n:Farm_n:Hatchery_38:Farm_14] 0.0 1.0 7093

b[(Intercept) Hatchery_n:Farm_n:Hatchery_39:Farm_2] 0.0 1.0 9985

b[(Intercept) Hatchery_n:Farm_n:Hatchery_40:Farm_1] 0.0 1.0 6919

b[(Intercept) Hatchery_n:Farm_n:Hatchery_40:Farm_10] 0.0 1.0 7326

b[(Intercept) Hatchery_n:Farm_n:Hatchery_40:Farm_11] 0.0 1.0 9429

b[(Intercept) Hatchery_n:Farm_n:Hatchery_40:Farm_16] 0.0 1.0 8262

b[(Intercept) Hatchery_n:Farm_n:Hatchery_40:Farm_17] 0.0 1.0 5913

b[(Intercept) Hatchery_n:Farm_n:Hatchery_40:Farm_9] 0.0 1.0 9464

b[(Intercept) Hatchery_n:Farm_n:Hatchery_41:Farm_16] 0.0 1.0 5831

b[(Intercept) Hatchery_n:Farm_n:Hatchery_42:Farm_11] 0.0 1.0 10163

b[(Intercept) Hatchery_n:Farm_n:Hatchery_43:Farm_11] 0.0 1.0 11017

b[(Intercept) Hatchery_n:Farm_n:Hatchery_45:Farm_2] 0.0 1.0 4506

b[(Intercept) Hatchery_n:Farm_n:Hatchery_46:Farm_19] 0.0 1.0 2796

b[(Intercept) Hatchery_n:Farm_n:Hatchery_47:Farm_18] 0.0 1.0 11623

b[(Intercept) Hatchery_n:Farm_n:Hatchery_48:Farm_18] 0.0 1.0 9842

b[(Intercept) Hatchery_n:Farm_n:Hatchery_49:Farm_16] 0.0 1.0 4866

b[(Intercept) Hatchery_n:Farm_n:Hatchery_5:Farm_18] 0.0 1.0 11412

b[(Intercept) Hatchery_n:Farm_n:Hatchery_5:Farm_19] 0.0 1.0 11032

b[(Intercept) Hatchery_n:Farm_n:Hatchery_50:Farm_4] 0.0 1.0 4356

b[(Intercept) Hatchery_n:Farm_n:Hatchery_51:Farm_14] 0.0 1.0 6763

b[(Intercept) Hatchery_n:Farm_n:Hatchery_51:Farm_18] 0.0 1.0 6072

b[(Intercept) Hatchery_n:Farm_n:Hatchery_52:Farm_17] 0.0 1.0 8961

b[(Intercept) Hatchery_n:Farm_n:Hatchery_53:Farm_19] 0.0 1.0 2912

b[(Intercept) Hatchery_n:Farm_n:Hatchery_54:Farm_14] 0.0 1.0 4765

b[(Intercept) Hatchery_n:Farm_n:Hatchery_55:Farm_16] 0.0 1.0 7760

b[(Intercept) Hatchery_n:Farm_n:Hatchery_56:Farm_10] 0.0 1.0 10114

b[(Intercept) Hatchery_n:Farm_n:Hatchery_57:Farm_19] 0.0 1.0 6042

b[(Intercept) Hatchery_n:Farm_n:Hatchery_58:Farm_3] 0.0 1.0 9997

b[(Intercept) Hatchery_n:Farm_n:Hatchery_59:Farm_16] 0.0 1.0 6123

b[(Intercept) Hatchery_n:Farm_n:Hatchery_59:Farm_17] 0.0 1.0 10990

b[(Intercept) Hatchery_n:Farm_n:Hatchery_6:Farm_4] 0.0 1.0 4988

b[(Intercept) Hatchery_n:Farm_n:Hatchery_60:Farm_17] 0.0 1.0 8368

b[(Intercept) Hatchery_n:Farm_n:Hatchery_60:Farm_9] 0.0 1.0 10175

b[(Intercept) Hatchery_n:Farm_n:Hatchery_61:Farm_5] 0.0 1.0 9806

b[(Intercept) Hatchery_n:Farm_n:Hatchery_62:Farm_17] 0.0 1.0 2770

b[(Intercept) Hatchery_n:Farm_n:Hatchery_63:Farm_1] 0.0 1.0 3719

b[(Intercept) Hatchery_n:Farm_n:Hatchery_63:Farm_4] 0.0 1.0 6904

b[(Intercept) Hatchery_n:Farm_n:Hatchery_64:Farm_5] 0.0 1.0 8196

b[(Intercept) Hatchery_n:Farm_n:Hatchery_64:Farm_6] 0.0 1.0 9880

b[(Intercept) Hatchery_n:Farm_n:Hatchery_65:Farm_14] 0.0 1.0 7606

b[(Intercept) Hatchery_n:Farm_n:Hatchery_66:Farm_2] 0.0 1.0 10617

b[(Intercept) Hatchery_n:Farm_n:Hatchery_67:Farm_12] 0.0 1.0 4626

b[(Intercept) Hatchery_n:Farm_n:Hatchery_67:Farm_9] 0.0 1.0 8419

b[(Intercept) Hatchery_n:Farm_n:Hatchery_68:Farm_12] 0.0 1.0 5202

b[(Intercept) Hatchery_n:Farm_n:Hatchery_69:Farm_12] 0.0 1.0 8600

b[(Intercept) Hatchery_n:Farm_n:Hatchery_7:Farm_13] 0.0 1.0 8920

b[(Intercept) Hatchery_n:Farm_n:Hatchery_8:Farm_7] 0.0 1.0 6661

b[(Intercept) Hatchery_n:Farm_n:Hatchery_9:Farm_19] 0.0 1.0 8394

b[(Intercept) Farm_n:Farm_1] 0.0 1.0 5446

b[(Intercept) Farm_n:Farm_10] 0.0 1.0 6133

b[(Intercept) Farm_n:Farm_11] 0.0 1.0 5953

b[(Intercept) Farm_n:Farm_12] 0.0 1.0 3169

b[(Intercept) Farm_n:Farm_13] 0.0 1.0 4776

b[(Intercept) Farm_n:Farm_14] 0.0 1.0 2780

b[(Intercept) Farm_n:Farm_15] 0.0 1.0 6526

b[(Intercept) Farm_n:Farm_16] 0.0 1.0 6414

b[(Intercept) Farm_n:Farm_17] 0.0 1.0 6301

b[(Intercept) Farm_n:Farm_18] 0.0 1.0 8178

b[(Intercept) Farm_n:Farm_19] 0.0 1.0 6339

b[(Intercept) Farm_n:Farm_2] 0.0 1.0 6019

b[(Intercept) Farm_n:Farm_3] 0.0 1.0 3879

b[(Intercept) Farm_n:Farm_4] 0.0 1.0 5578

b[(Intercept) Farm_n:Farm_5] 0.0 1.0 4968

b[(Intercept) Farm_n:Farm_6] 0.0 1.0 3745

b[(Intercept) Farm_n:Farm_7] 0.0 1.0 4205

b[(Intercept) Farm_n:Farm_8] 0.0 1.0 6740

b[(Intercept) Farm_n:Farm_9] 0.0 1.0 3989

reciprocal_dispersion 0.0 1.0 2657

Sigma[Hatchery_n:Farm_n:(Intercept),(Intercept)] 0.0 1.0 1548

Sigma[Farm_n:(Intercept),(Intercept)] 0.0 1.0 2397

mean_PPD 0.0 1.0 8231

log-posterior 0.3 1.0 1485

For each parameter, mcse is Monte Carlo standard error, n_eff is a crude measure of effective sample size, and Rhat is the potential scale reduction factor on split chains (at convergence Rhat=1).

# ***Polymyxin hierarchical Bayesian negative-binomial regression model***

Relevant for model fit: the Monte Carlo Standard Error (MCSE) for each parameter was small, indicating high precision in the posterior mean estimates. The potential scale reduction factor (Rhat) values were close to 1.00, suggesting that the model had likely converged effectively. Additionally, the effective sample size (n_eff) was sufficiently large, reflecting a high number of independent samples and providing confidence in the reliability of the parameter estimates.

> summary(fit.pol,probs=c(0.025,0.975))

Model Info:

function: stan_glmer

family: neg_binomial_2 [log]

formula: POL_count ~ factor(Cycle) + factor(Season) + N_Broilers + firstweekmort +

Ext_Bio + Emp_Eq + Purpose + F_W_Mng + Int_Bio + Mng_Vet_D +

Mov_Pur + factor(Phase) + Purpose + (1 | Farm_n/Hatchery_n)

algorithm: sampling

sample: 8000 (posterior sample size)

priors: see help('prior_summary')

observations: 142

groups: Hatchery_n:Farm_n (95), Farm_n (19)

Estimates:

mean sd 2.5% 97.5%

(Intercept) -2.0 1.7 -5.6 1.1

factor(Cycle)2 0.0 0.4 -0.7 0.8

factor(Cycle)3 -0.2 0.4 -1.1 0.5

factor(Cycle)4 0.0 0.4 -0.8 0.8

factor(Cycle)5 -0.3 0.5 -1.3 0.7

factor(Cycle)6 -1.8 2.4 -7.8 1.3

factor(Season)1 0.1 0.3 -0.5 0.8

N_Broilers 0.0 0.0 0.0 0.0

firstweekmort 0.0 0.1 -0.2 0.3

Ext_Bio -0.8 1.3 -4.0 1.4

Emp_Eq -0.4 1.0 -2.7 1.3

Purpose 0.0 0.6 -1.2 1.1

F_W_Mng 0.3 1.8 -3.0 4.5

Int_Bio -0.3 1.9 -4.4 3.6

Mng_Vet_D -0.5 0.8 -2.4 0.9

Mov_Pur 0.2 0.9 -1.5 2.4

factor(Phase)Pre 2.6 0.8 1.1 4.2

b[(Intercept) Hatchery_n:Farm_n:Hatchery_1:Farm_18] 0.1 0.4 -0.5 1.1

b[(Intercept) Hatchery_n:Farm_n:Hatchery_1:Farm_19] 0.1 0.4 -0.5 1.0

b[(Intercept) Hatchery_n:Farm_n:Hatchery_10:Farm_1] 0.0 0.3 -0.7 0.8

b[(Intercept) Hatchery_n:Farm_n:Hatchery_10:Farm_2] 0.0 0.4 -0.8 0.9

b[(Intercept) Hatchery_n:Farm_n:Hatchery_11:Farm_3] 0.0 0.4 -0.9 0.7

b[(Intercept) Hatchery_n:Farm_n:Hatchery_12:Farm_10] 0.0 0.4 -0.9 0.7

b[(Intercept) Hatchery_n:Farm_n:Hatchery_13:Farm_13] 0.1 0.4 -0.5 1.1

b[(Intercept) Hatchery_n:Farm_n:Hatchery_14:Farm_11] 0.1 0.4 -0.4 1.3

b[(Intercept) Hatchery_n:Farm_n:Hatchery_15:Farm_13] 0.0 0.4 -0.9 0.8

b[(Intercept) Hatchery_n:Farm_n:Hatchery_16:Farm_14] 0.0 0.4 -0.8 0.8

b[(Intercept) Hatchery_n:Farm_n:Hatchery_17:Farm_15] 0.0 0.3 -0.6 0.9

b[(Intercept) Hatchery_n:Farm_n:Hatchery_18:Farm_3] 0.0 0.4 -1.0 0.7

b[(Intercept) Hatchery_n:Farm_n:Hatchery_18:Farm_4] 0.0 0.4 -0.9 0.7

b[(Intercept) Hatchery_n:Farm_n:Hatchery_19:Farm_3] 0.0 0.4 -0.9 0.8

b[(Intercept) Hatchery_n:Farm_n:Hatchery_2:Farm_15] 0.1 0.4 -0.6 1.0

b[(Intercept) Hatchery_n:Farm_n:Hatchery_2:Farm_18] 0.0 0.4 -1.0 0.7

b[(Intercept) Hatchery_n:Farm_n:Hatchery_2:Farm_7] -0.1 0.4 -1.1 0.6

b[(Intercept) Hatchery_n:Farm_n:Hatchery_2:Farm_8] 0.0 0.3 -0.8 0.7

b[(Intercept) Hatchery_n:Farm_n:Hatchery_2:Farm_9] 0.0 0.4 -0.9 0.8

b[(Intercept) Hatchery_n:Farm_n:Hatchery_20:Farm_12] 0.0 0.3 -0.6 0.9

b[(Intercept) Hatchery_n:Farm_n:Hatchery_21:Farm_4] 0.0 0.4 -0.8 0.8

b[(Intercept) Hatchery_n:Farm_n:Hatchery_22:Farm_14] -0.1 0.4 -1.1 0.6

b[(Intercept) Hatchery_n:Farm_n:Hatchery_22:Farm_15] -0.1 0.4 -1.0 0.7

b[(Intercept) Hatchery_n:Farm_n:Hatchery_22:Farm_2] 0.0 0.4 -0.6 0.9

b[(Intercept) Hatchery_n:Farm_n:Hatchery_22:Farm_5] 0.1 0.3 -0.5 1.0

b[(Intercept) Hatchery_n:Farm_n:Hatchery_22:Farm_6] 0.0 0.4 -0.9 0.7

b[(Intercept) Hatchery_n:Farm_n:Hatchery_23:Farm_5] -0.1 0.4 -1.0 0.7

b[(Intercept) Hatchery_n:Farm_n:Hatchery_24:Farm_6] 0.0 0.4 -0.9 0.7

b[(Intercept) Hatchery_n:Farm_n:Hatchery_25:Farm_6] 0.0 0.4 -0.9 0.7

b[(Intercept) Hatchery_n:Farm_n:Hatchery_26:Farm_5] 0.0 0.4 -0.9 0.8

b[(Intercept) Hatchery_n:Farm_n:Hatchery_27:Farm_5] 0.0 0.4 -0.8 0.7

b[(Intercept) Hatchery_n:Farm_n:Hatchery_28:Farm_13] 0.0 0.4 -0.9 0.8

b[(Intercept) Hatchery_n:Farm_n:Hatchery_29:Farm_12] 0.0 0.3 -0.7 0.9

b[(Intercept) Hatchery_n:Farm_n:Hatchery_29:Farm_13] 0.1 0.4 -0.5 1.1

b[(Intercept) Hatchery_n:Farm_n:Hatchery_29:Farm_6] 0.0 0.4 -0.8 0.8

b[(Intercept) Hatchery_n:Farm_n:Hatchery_3:Farm_8] 0.2 0.5 -0.3 1.6

b[(Intercept) Hatchery_n:Farm_n:Hatchery_30:Farm_13] 0.0 0.4 -0.7 0.9

b[(Intercept) Hatchery_n:Farm_n:Hatchery_31:Farm_10] 0.1 0.4 -0.6 1.0

b[(Intercept) Hatchery_n:Farm_n:Hatchery_31:Farm_11] 0.1 0.4 -0.6 1.0

b[(Intercept) Hatchery_n:Farm_n:Hatchery_31:Farm_15] 0.0 0.4 -0.9 0.7

b[(Intercept) Hatchery_n:Farm_n:Hatchery_32:Farm_8] 0.0 0.4 -0.9 0.7

b[(Intercept) Hatchery_n:Farm_n:Hatchery_33:Farm_13] 0.0 0.4 -0.9 0.8

b[(Intercept) Hatchery_n:Farm_n:Hatchery_34:Farm_7] 0.0 0.4 -0.9 0.7

b[(Intercept) Hatchery_n:Farm_n:Hatchery_34:Farm_8] 0.0 0.4 -0.7 0.8

b[(Intercept) Hatchery_n:Farm_n:Hatchery_35:Farm_9] 0.0 0.4 -0.9 0.7

b[(Intercept) Hatchery_n:Farm_n:Hatchery_36:Farm_12] 0.0 0.4 -0.9 0.7

b[(Intercept) Hatchery_n:Farm_n:Hatchery_37:Farm_19] 0.0 0.4 -1.0 0.7

b[(Intercept) Hatchery_n:Farm_n:Hatchery_38:Farm_14] 0.0 0.4 -0.9 0.8

b[(Intercept) Hatchery_n:Farm_n:Hatchery_39:Farm_2] 0.1 0.4 -0.5 1.1

b[(Intercept) Hatchery_n:Farm_n:Hatchery_40:Farm_1] 0.0 0.3 -0.6 0.9

b[(Intercept) Hatchery_n:Farm_n:Hatchery_40:Farm_10] 0.0 0.3 -0.8 0.7

b[(Intercept) Hatchery_n:Farm_n:Hatchery_40:Farm_11] -0.1 0.4 -1.3 0.5

b[(Intercept) Hatchery_n:Farm_n:Hatchery_40:Farm_16] 0.1 0.4 -0.5 1.0

b[(Intercept) Hatchery_n:Farm_n:Hatchery_40:Farm_17] -0.1 0.4 -1.1 0.6

b[(Intercept) Hatchery_n:Farm_n:Hatchery_40:Farm_9] 0.0 0.4 -0.9 0.7

b[(Intercept) Hatchery_n:Farm_n:Hatchery_41:Farm_16] 0.0 0.4 -0.9 0.8

b[(Intercept) Hatchery_n:Farm_n:Hatchery_42:Farm_11] 0.0 0.4 -0.9 0.7

b[(Intercept) Hatchery_n:Farm_n:Hatchery_43:Farm_11] 0.0 0.4 -0.9 0.8

b[(Intercept) Hatchery_n:Farm_n:Hatchery_45:Farm_2] -0.1 0.4 -1.0 0.6

b[(Intercept) Hatchery_n:Farm_n:Hatchery_46:Farm_19] 0.0 0.4 -0.8 0.8

b[(Intercept) Hatchery_n:Farm_n:Hatchery_47:Farm_18] 0.0 0.4 -0.9 0.8

b[(Intercept) Hatchery_n:Farm_n:Hatchery_48:Farm_18] 0.0 0.4 -0.8 0.8

b[(Intercept) Hatchery_n:Farm_n:Hatchery_49:Farm_16] 0.0 0.4 -0.9 0.8

b[(Intercept) Hatchery_n:Farm_n:Hatchery_5:Farm_18] -0.1 0.4 -1.0 0.7

b[(Intercept) Hatchery_n:Farm_n:Hatchery_5:Farm_19] 0.0 0.3 -0.7 0.8

b[(Intercept) Hatchery_n:Farm_n:Hatchery_50:Farm_4] 0.0 0.4 -1.0 0.6

b[(Intercept) Hatchery_n:Farm_n:Hatchery_51:Farm_14] 0.0 0.4 -0.9 0.8

b[(Intercept) Hatchery_n:Farm_n:Hatchery_51:Farm_18] 0.0 0.4 -0.8 0.8

b[(Intercept) Hatchery_n:Farm_n:Hatchery_52:Farm_17] 0.0 0.4 -0.9 0.8

b[(Intercept) Hatchery_n:Farm_n:Hatchery_53:Farm_19] 0.0 0.4 -0.9 0.7

b[(Intercept) Hatchery_n:Farm_n:Hatchery_54:Farm_14] 0.0 0.4 -0.9 0.8

b[(Intercept) Hatchery_n:Farm_n:Hatchery_55:Farm_16] 0.0 0.4 -0.9 0.8

b[(Intercept) Hatchery_n:Farm_n:Hatchery_56:Farm_10] 0.0 0.4 -0.8 0.8

b[(Intercept) Hatchery_n:Farm_n:Hatchery_57:Farm_19] 0.0 0.4 -0.9 0.8

b[(Intercept) Hatchery_n:Farm_n:Hatchery_58:Farm_3] 0.0 0.4 -0.9 0.7

b[(Intercept) Hatchery_n:Farm_n:Hatchery_59:Farm_16] 0.1 0.4 -0.4 1.2

b[(Intercept) Hatchery_n:Farm_n:Hatchery_59:Farm_17] 0.0 0.4 -0.8 0.8

b[(Intercept) Hatchery_n:Farm_n:Hatchery_6:Farm_4] 0.0 0.4 -0.9 0.8

b[(Intercept) Hatchery_n:Farm_n:Hatchery_60:Farm_17] -0.1 0.4 -1.0 0.7

b[(Intercept) Hatchery_n:Farm_n:Hatchery_60:Farm_9] 0.0 0.4 -0.9 0.7

b[(Intercept) Hatchery_n:Farm_n:Hatchery_61:Farm_5] 0.1 0.4 -0.5 1.1

b[(Intercept) Hatchery_n:Farm_n:Hatchery_62:Farm_17] 0.1 0.4 -0.5 1.2

b[(Intercept) Hatchery_n:Farm_n:Hatchery_63:Farm_1] 0.0 0.3 -0.7 0.8

b[(Intercept) Hatchery_n:Farm_n:Hatchery_63:Farm_4] 0.0 0.4 -0.9 0.7

b[(Intercept) Hatchery_n:Farm_n:Hatchery_64:Farm_5] -0.1 0.4 -1.0 0.7

b[(Intercept) Hatchery_n:Farm_n:Hatchery_64:Farm_6] 0.0 0.4 -1.0 0.7

b[(Intercept) Hatchery_n:Farm_n:Hatchery_65:Farm_14] 0.0 0.4 -0.9 0.8

b[(Intercept) Hatchery_n:Farm_n:Hatchery_66:Farm_2] -0.1 0.4 -1.0 0.6

b[(Intercept) Hatchery_n:Farm_n:Hatchery_67:Farm_12] 0.0 0.3 -0.6 0.9

b[(Intercept) Hatchery_n:Farm_n:Hatchery_67:Farm_9] 0.0 0.4 -0.9 0.7

b[(Intercept) Hatchery_n:Farm_n:Hatchery_68:Farm_12] 0.0 0.4 -1.0 0.7

b[(Intercept) Hatchery_n:Farm_n:Hatchery_69:Farm_12] 0.0 0.4 -0.9 0.7

b[(Intercept) Hatchery_n:Farm_n:Hatchery_7:Farm_13] -0.1 0.4 -1.0 0.6

b[(Intercept) Hatchery_n:Farm_n:Hatchery_8:Farm_7] 0.0 0.4 -0.9 0.7

b[(Intercept) Hatchery_n:Farm_n:Hatchery_9:Farm_19] 0.1 0.3 -0.6 0.9

b[(Intercept) Farm_n:Farm_1] 0.6 0.8 -0.6 2.4

b[(Intercept) Farm_n:Farm_10] 0.1 0.7 -1.3 1.7

b[(Intercept) Farm_n:Farm_11] 0.2 0.7 -1.2 1.9

b[(Intercept) Farm_n:Farm_12] 0.3 0.7 -0.9 1.9

b[(Intercept) Farm_n:Farm_13] 0.7 0.8 -0.5 2.6

b[(Intercept) Farm_n:Farm_14] -0.8 1.1 -3.3 0.8

b[(Intercept) Farm_n:Farm_15] 0.1 0.7 -1.1 1.6

b[(Intercept) Farm_n:Farm_16] 1.1 1.0 -0.2 3.3

b[(Intercept) Farm_n:Farm_17] 0.0 0.7 -1.4 1.6

b[(Intercept) Farm_n:Farm_18] 0.0 0.7 -1.4 1.4

b[(Intercept) Farm_n:Farm_19] 0.4 0.7 -0.8 2.2

b[(Intercept) Farm_n:Farm_2] 0.2 0.7 -1.1 1.8

b[(Intercept) Farm_n:Farm_3] -0.9 1.1 -3.5 0.6

b[(Intercept) Farm_n:Farm_4] -0.9 1.1 -3.5 0.6

b[(Intercept) Farm_n:Farm_5] 0.3 0.6 -0.8 1.8

b[(Intercept) Farm_n:Farm_6] -0.9 1.1 -3.6 0.5

b[(Intercept) Farm_n:Farm_7] -0.9 1.1 -3.6 0.6

b[(Intercept) Farm_n:Farm_8] 1.0 0.9 -0.2 3.0

b[(Intercept) Farm_n:Farm_9] -0.9 1.1 -3.6 0.6

reciprocal_dispersion 0.6 0.3 0.2 1.2

Sigma[Hatchery_n:Farm_n:(Intercept),(Intercept)] 0.1 0.3 0.0 0.9

Sigma[Farm_n:(Intercept),(Intercept)] 1.2 1.4 0.0 4.8

Fit Diagnostics:

mean sd 2.5% 97.5%

mean_PPD 1.2 0.5 0.6 2.2

The mean_ppd is the sample average posterior predictive distribution of the outcome variable (for details see help('summary.stanreg')).

MCMC diagnostics

mcse Rhat n_eff

(Intercept) 0.0 1.0 6254

factor(Cycle)2 0.0 1.0 9677

factor(Cycle)3 0.0 1.0 8613

factor(Cycle)4 0.0 1.0 8244

factor(Cycle)5 0.0 1.0 6493

factor(Cycle)6 0.0 1.0 7195

factor(Season)1 0.0 1.0 10441

N_Broilers 0.0 1.0 6191

firstweekmort 0.0 1.0 4540

Ext_Bio 0.0 1.0 5901

Emp_Eq 0.0 1.0 7152

Purpose 0.0 1.0 8381

F_W_Mng 0.0 1.0 7188

Int_Bio 0.0 1.0 8496

Mng_Vet_D 0.0 1.0 5589

Mov_Pur 0.0 1.0 6740

factor(Phase)Pre 0.0 1.0 2525

b[(Intercept) Hatchery_n:Farm_n:Hatchery_1:Farm_18] 0.0 1.0 4118

b[(Intercept) Hatchery_n:Farm_n:Hatchery_1:Farm_19] 0.0 1.0 6510

b[(Intercept) Hatchery_n:Farm_n:Hatchery_10:Farm_1] 0.0 1.0 9328

b[(Intercept) Hatchery_n:Farm_n:Hatchery_10:Farm_2] 0.0 1.0 9596

b[(Intercept) Hatchery_n:Farm_n:Hatchery_11:Farm_3] 0.0 1.0 9123

b[(Intercept) Hatchery_n:Farm_n:Hatchery_12:Farm_10] 0.0 1.0 6123

b[(Intercept) Hatchery_n:Farm_n:Hatchery_13:Farm_13] 0.0 1.0 4612

b[(Intercept) Hatchery_n:Farm_n:Hatchery_14:Farm_11] 0.0 1.0 3636

b[(Intercept) Hatchery_n:Farm_n:Hatchery_15:Farm_13] 0.0 1.0 9000

b[(Intercept) Hatchery_n:Farm_n:Hatchery_16:Farm_14] 0.0 1.0 8282

b[(Intercept) Hatchery_n:Farm_n:Hatchery_17:Farm_15] 0.0 1.0 6334

b[(Intercept) Hatchery_n:Farm_n:Hatchery_18:Farm_3] 0.0 1.0 8114

b[(Intercept) Hatchery_n:Farm_n:Hatchery_18:Farm_4] 0.0 1.0 7650

b[(Intercept) Hatchery_n:Farm_n:Hatchery_19:Farm_3] 0.0 1.0 8762

b[(Intercept) Hatchery_n:Farm_n:Hatchery_2:Farm_15] 0.0 1.0 6688

b[(Intercept) Hatchery_n:Farm_n:Hatchery_2:Farm_18] 0.0 1.0 8632

b[(Intercept) Hatchery_n:Farm_n:Hatchery_2:Farm_7] 0.0 1.0 5841

b[(Intercept) Hatchery_n:Farm_n:Hatchery_2:Farm_8] 0.0 1.0 7080

b[(Intercept) Hatchery_n:Farm_n:Hatchery_2:Farm_9] 0.0 1.0 9022

b[(Intercept) Hatchery_n:Farm_n:Hatchery_20:Farm_12] 0.0 1.0 9075

b[(Intercept) Hatchery_n:Farm_n:Hatchery_21:Farm_4] 0.0 1.0 9996

b[(Intercept) Hatchery_n:Farm_n:Hatchery_22:Farm_14] 0.0 1.0 6267

b[(Intercept) Hatchery_n:Farm_n:Hatchery_22:Farm_15] 0.0 1.0 7280

b[(Intercept) Hatchery_n:Farm_n:Hatchery_22:Farm_2] 0.0 1.0 7676

b[(Intercept) Hatchery_n:Farm_n:Hatchery_22:Farm_5] 0.0 1.0 6679

b[(Intercept) Hatchery_n:Farm_n:Hatchery_22:Farm_6] 0.0 1.0 7489

b[(Intercept) Hatchery_n:Farm_n:Hatchery_23:Farm_5] 0.0 1.0 6764

b[(Intercept) Hatchery_n:Farm_n:Hatchery_24:Farm_6] 0.0 1.0 8461

b[(Intercept) Hatchery_n:Farm_n:Hatchery_25:Farm_6] 0.0 1.0 7468

b[(Intercept) Hatchery_n:Farm_n:Hatchery_26:Farm_5] 0.0 1.0 8586

b[(Intercept) Hatchery_n:Farm_n:Hatchery_27:Farm_5] 0.0 1.0 7884

b[(Intercept) Hatchery_n:Farm_n:Hatchery_28:Farm_13] 0.0 1.0 9308

b[(Intercept) Hatchery_n:Farm_n:Hatchery_29:Farm_12] 0.0 1.0 7707

b[(Intercept) Hatchery_n:Farm_n:Hatchery_29:Farm_13] 0.0 1.0 3869

b[(Intercept) Hatchery_n:Farm_n:Hatchery_29:Farm_6] 0.0 1.0 9199

b[(Intercept) Hatchery_n:Farm_n:Hatchery_3:Farm_8] 0.0 1.0 2992

b[(Intercept) Hatchery_n:Farm_n:Hatchery_30:Farm_13] 0.0 1.0 8262

b[(Intercept) Hatchery_n:Farm_n:Hatchery_31:Farm_10] 0.0 1.0 6624

b[(Intercept) Hatchery_n:Farm_n:Hatchery_31:Farm_11] 0.0 1.0 6370

b[(Intercept) Hatchery_n:Farm_n:Hatchery_31:Farm_15] 0.0 1.0 7783

b[(Intercept) Hatchery_n:Farm_n:Hatchery_32:Farm_8] 0.0 1.0 8432

b[(Intercept) Hatchery_n:Farm_n:Hatchery_33:Farm_13] 0.0 1.0 9440

b[(Intercept) Hatchery_n:Farm_n:Hatchery_34:Farm_7] 0.0 1.0 8644

b[(Intercept) Hatchery_n:Farm_n:Hatchery_34:Farm_8] 0.0 1.0 9590

b[(Intercept) Hatchery_n:Farm_n:Hatchery_35:Farm_9] 0.0 1.0 9119

b[(Intercept) Hatchery_n:Farm_n:Hatchery_36:Farm_12] 0.0 1.0 8421

b[(Intercept) Hatchery_n:Farm_n:Hatchery_37:Farm_19] 0.0 1.0 8106

b[(Intercept) Hatchery_n:Farm_n:Hatchery_38:Farm_14] 0.0 1.0 9643

b[(Intercept) Hatchery_n:Farm_n:Hatchery_39:Farm_2] 0.0 1.0 4875

b[(Intercept) Hatchery_n:Farm_n:Hatchery_40:Farm_1] 0.0 1.0 7292

b[(Intercept) Hatchery_n:Farm_n:Hatchery_40:Farm_10] 0.0 1.0 8229

b[(Intercept) Hatchery_n:Farm_n:Hatchery_40:Farm_11] 0.0 1.0 4191

b[(Intercept) Hatchery_n:Farm_n:Hatchery_40:Farm_16] 0.0 1.0 5683

b[(Intercept) Hatchery_n:Farm_n:Hatchery_40:Farm_17] 0.0 1.0 6725

b[(Intercept) Hatchery_n:Farm_n:Hatchery_40:Farm_9] 0.0 1.0 9141

b[(Intercept) Hatchery_n:Farm_n:Hatchery_41:Farm_16] 0.0 1.0 8238

b[(Intercept) Hatchery_n:Farm_n:Hatchery_42:Farm_11] 0.0 1.0 8672

b[(Intercept) Hatchery_n:Farm_n:Hatchery_43:Farm_11] 0.0 1.0 9581

b[(Intercept) Hatchery_n:Farm_n:Hatchery_45:Farm_2] 0.0 1.0 8417

b[(Intercept) Hatchery_n:Farm_n:Hatchery_46:Farm_19] 0.0 1.0 9628

b[(Intercept) Hatchery_n:Farm_n:Hatchery_47:Farm_18] 0.0 1.0 8882

b[(Intercept) Hatchery_n:Farm_n:Hatchery_48:Farm_18] 0.0 1.0 9134

b[(Intercept) Hatchery_n:Farm_n:Hatchery_49:Farm_16] 0.0 1.0 9134

b[(Intercept) Hatchery_n:Farm_n:Hatchery_5:Farm_18] 0.0 1.0 7056

b[(Intercept) Hatchery_n:Farm_n:Hatchery_5:Farm_19] 0.0 1.0 9576

b[(Intercept) Hatchery_n:Farm_n:Hatchery_50:Farm_4] 0.0 1.0 8114

b[(Intercept) Hatchery_n:Farm_n:Hatchery_51:Farm_14] 0.0 1.0 9957

b[(Intercept) Hatchery_n:Farm_n:Hatchery_51:Farm_18] 0.0 1.0 8375

b[(Intercept) Hatchery_n:Farm_n:Hatchery_52:Farm_17] 0.0 1.0 9387

b[(Intercept) Hatchery_n:Farm_n:Hatchery_53:Farm_19] 0.0 1.0 8763

b[(Intercept) Hatchery_n:Farm_n:Hatchery_54:Farm_14] 0.0 1.0 8174

b[(Intercept) Hatchery_n:Farm_n:Hatchery_55:Farm_16] 0.0 1.0 8199

b[(Intercept) Hatchery_n:Farm_n:Hatchery_56:Farm_10] 0.0 1.0 8235

b[(Intercept) Hatchery_n:Farm_n:Hatchery_57:Farm_19] 0.0 1.0 8252

b[(Intercept) Hatchery_n:Farm_n:Hatchery_58:Farm_3] 0.0 1.0 9641

b[(Intercept) Hatchery_n:Farm_n:Hatchery_59:Farm_16] 0.0 1.0 4367

b[(Intercept) Hatchery_n:Farm_n:Hatchery_59:Farm_17] 0.0 1.0 9304

b[(Intercept) Hatchery_n:Farm_n:Hatchery_6:Farm_4] 0.0 1.0 8001

b[(Intercept) Hatchery_n:Farm_n:Hatchery_60:Farm_17] 0.0 1.0 7933

b[(Intercept) Hatchery_n:Farm_n:Hatchery_60:Farm_9] 0.0 1.0 9176

b[(Intercept) Hatchery_n:Farm_n:Hatchery_61:Farm_5] 0.0 1.0 4821

b[(Intercept) Hatchery_n:Farm_n:Hatchery_62:Farm_17] 0.0 1.0 3835

b[(Intercept) Hatchery_n:Farm_n:Hatchery_63:Farm_1] 0.0 1.0 8969

b[(Intercept) Hatchery_n:Farm_n:Hatchery_63:Farm_4] 0.0 1.0 9374

b[(Intercept) Hatchery_n:Farm_n:Hatchery_64:Farm_5] 0.0 1.0 7917

b[(Intercept) Hatchery_n:Farm_n:Hatchery_64:Farm_6] 0.0 1.0 7770

b[(Intercept) Hatchery_n:Farm_n:Hatchery_65:Farm_14] 0.0 1.0 8075

b[(Intercept) Hatchery_n:Farm_n:Hatchery_66:Farm_2] 0.0 1.0 7075

b[(Intercept) Hatchery_n:Farm_n:Hatchery_67:Farm_12] 0.0 1.0 7918

b[(Intercept) Hatchery_n:Farm_n:Hatchery_67:Farm_9] 0.0 1.0 8677

b[(Intercept) Hatchery_n:Farm_n:Hatchery_68:Farm_12] 0.0 1.0 8081

b[(Intercept) Hatchery_n:Farm_n:Hatchery_69:Farm_12] 0.0 1.0 7953

b[(Intercept) Hatchery_n:Farm_n:Hatchery_7:Farm_13] 0.0 1.0 6778

b[(Intercept) Hatchery_n:Farm_n:Hatchery_8:Farm_7] 0.0 1.0 7089

b[(Intercept) Hatchery_n:Farm_n:Hatchery_9:Farm_19] 0.0 1.0 6897

b[(Intercept) Farm_n:Farm_1] 0.0 1.0 2085

b[(Intercept) Farm_n:Farm_10] 0.0 1.0 5341

b[(Intercept) Farm_n:Farm_11] 0.0 1.0 6096

b[(Intercept) Farm_n:Farm_12] 0.0 1.0 3183

b[(Intercept) Farm_n:Farm_13] 0.0 1.0 1591

b[(Intercept) Farm_n:Farm_14] 0.0 1.0 1913

b[(Intercept) Farm_n:Farm_15] 0.0 1.0 6041

b[(Intercept) Farm_n:Farm_16] 0.0 1.0 1117

b[(Intercept) Farm_n:Farm_17] 0.0 1.0 7845

b[(Intercept) Farm_n:Farm_18] 0.0 1.0 8537

b[(Intercept) Farm_n:Farm_19] 0.0 1.0 2749

b[(Intercept) Farm_n:Farm_2] 0.0 1.0 4926

b[(Intercept) Farm_n:Farm_3] 0.0 1.0 1729

b[(Intercept) Farm_n:Farm_4] 0.0 1.0 1602

b[(Intercept) Farm_n:Farm_5] 0.0 1.0 3971

b[(Intercept) Farm_n:Farm_6] 0.0 1.0 1436

b[(Intercept) Farm_n:Farm_7] 0.0 1.0 1664

b[(Intercept) Farm_n:Farm_8] 0.0 1.0 1106

b[(Intercept) Farm_n:Farm_9] 0.0 1.0 1636

reciprocal_dispersion 0.0 1.0 1056

Sigma[Hatchery_n:Farm_n:(Intercept),(Intercept)] 0.0 1.0 1899

Sigma[Farm_n:(Intercept),(Intercept)] 0.0 1.0 925

mean_PPD 0.0 1.0 8245

log-posterior 0.4 1.0 860

For each parameter, mcse is Monte Carlo standard error, n_eff is a crude measure of effective sample size, and Rhat is the potential scale reduction factor on split chains (at convergence Rhat=1).

# ***Fluoroquinolone hierarchical Bayesian negative-binomial regression model***

Relevant for model fit: the Monte Carlo Standard Error (MCSE) for each parameter was small, indicating high precision in the posterior mean estimates. The potential scale reduction factor (Rhat) values were close to 1.00, suggesting that the model had likely converged effectively. Additionally, the effective sample size (n_eff) was sufficiently large, reflecting a high number of independent samples and providing confidence in the reliability of the parameter estimates.

> summary(fit.flu,probs=c(0.025,0.975))

Model Info:

function: stan_glmer

family: neg_binomial_2 [log]

formula: FLU_count ~ factor(Cycle) + factor(Season) + N_Broilers + firstweekmort +

Ext_Bio + Emp_Eq + F_W_Mng + Int_Bio + Mng_Vet_D + Mov_Pur +

factor(Phase) + (1 | Farm_n/Hatchery_n)

algorithm: sampling

sample: 8000 (posterior sample size)

priors: see help('prior_summary')

observations: 143

groups: Hatchery_n:Farm_n (95), Farm_n (19)

Estimates:

mean sd 2.5% 97.5%

(Intercept) 0.1 0.5 -1.0 1.2

factor(Cycle)2 0.0 0.1 -0.1 0.2

factor(Cycle)3 0.0 0.1 -0.2 0.1

factor(Cycle)4 0.0 0.1 -0.1 0.2

factor(Cycle)5 0.0 0.1 -0.3 0.2

factor(Cycle)6 0.0 0.2 -0.5 0.3

factor(Season)1 0.0 0.1 -0.1 0.2

N_Broilers 0.0 0.0 0.0 0.0

firstweekmort 0.0 0.0 -0.1 0.0

Ext_Bio 0.0 0.2 -0.3 0.5

Emp_Eq 0.0 0.2 -0.3 0.4

F_W_Mng 0.0 0.3 -0.6 0.6

Int_Bio 0.0 0.3 -0.7 0.6

Mng_Vet_D 0.0 0.1 -0.2 0.3

Mov_Pur 0.0 0.2 -0.4 0.3

factor(Phase)Pre 0.0 0.1 -0.1 0.1

b[(Intercept) Hatchery_n:Farm_n:Hatchery_1:Farm_18] 0.1 0.5 -0.9 1.3

b[(Intercept) Hatchery_n:Farm_n:Hatchery_1:Farm_19] -0.1 0.6 -1.4 1.1

b[(Intercept) Hatchery_n:Farm_n:Hatchery_10:Farm_1] -0.1 0.5 -1.2 1.0

b[(Intercept) Hatchery_n:Farm_n:Hatchery_10:Farm_2] -0.4 0.6 -1.7 0.6

b[(Intercept) Hatchery_n:Farm_n:Hatchery_11:Farm_3] 0.1 0.5 -0.8 1.2

b[(Intercept) Hatchery_n:Farm_n:Hatchery_12:Farm_10] 0.2 0.5 -0.7 1.3

b[(Intercept) Hatchery_n:Farm_n:Hatchery_13:Farm_13] -0.1 0.6 -1.4 1.0

b[(Intercept) Hatchery_n:Farm_n:Hatchery_14:Farm_11] -0.4 0.6 -1.7 0.7

b[(Intercept) Hatchery_n:Farm_n:Hatchery_15:Farm_13] -0.1 0.6 -1.4 1.0

b[(Intercept) Hatchery_n:Farm_n:Hatchery_16:Farm_14] 0.1 0.5 -0.8 1.0

b[(Intercept) Hatchery_n:Farm_n:Hatchery_17:Farm_15] 0.0 0.5 -0.9 1.0

b[(Intercept) Hatchery_n:Farm_n:Hatchery_18:Farm_3] 0.1 0.5 -0.8 1.0

b[(Intercept) Hatchery_n:Farm_n:Hatchery_18:Farm_4] 0.1 0.4 -0.7 1.0

b[(Intercept) Hatchery_n:Farm_n:Hatchery_19:Farm_3] 0.2 0.5 -0.6 1.3

b[(Intercept) Hatchery_n:Farm_n:Hatchery_2:Farm_15] 0.1 0.5 -0.8 1.2

b[(Intercept) Hatchery_n:Farm_n:Hatchery_2:Farm_18] -0.1 0.6 -1.5 1.0

b[(Intercept) Hatchery_n:Farm_n:Hatchery_2:Farm_7] 0.4 0.5 -0.6 1.6

b[(Intercept) Hatchery_n:Farm_n:Hatchery_2:Farm_8] -0.5 0.6 -1.8 0.5

b[(Intercept) Hatchery_n:Farm_n:Hatchery_2:Farm_9] -0.1 0.6 -1.3 1.1

b[(Intercept) Hatchery_n:Farm_n:Hatchery_20:Farm_12] -0.1 0.6 -1.4 1.2

b[(Intercept) Hatchery_n:Farm_n:Hatchery_21:Farm_4] -0.1 0.5 -1.1 0.9

b[(Intercept) Hatchery_n:Farm_n:Hatchery_22:Farm_14] 0.1 0.4 -0.6 0.9

b[(Intercept) Hatchery_n:Farm_n:Hatchery_22:Farm_15] 0.0 0.5 -1.0 1.0

b[(Intercept) Hatchery_n:Farm_n:Hatchery_22:Farm_2] 0.5 0.6 -0.4 1.7

b[(Intercept) Hatchery_n:Farm_n:Hatchery_22:Farm_5] -0.1 0.6 -1.4 1.1

b[(Intercept) Hatchery_n:Farm_n:Hatchery_22:Farm_6] -0.4 0.6 -1.7 0.6

b[(Intercept) Hatchery_n:Farm_n:Hatchery_23:Farm_5] 0.0 0.6 -1.4 1.2

b[(Intercept) Hatchery_n:Farm_n:Hatchery_24:Farm_6] 0.2 0.5 -0.7 1.3

b[(Intercept) Hatchery_n:Farm_n:Hatchery_25:Farm_6] 0.7 0.5 -0.2 1.7

b[(Intercept) Hatchery_n:Farm_n:Hatchery_26:Farm_5] -0.1 0.6 -1.4 1.1

b[(Intercept) Hatchery_n:Farm_n:Hatchery_27:Farm_5] -0.1 0.6 -1.4 1.1

b[(Intercept) Hatchery_n:Farm_n:Hatchery_28:Farm_13] -0.1 0.6 -1.4 1.0

b[(Intercept) Hatchery_n:Farm_n:Hatchery_29:Farm_12] -0.1 0.6 -1.4 1.1

b[(Intercept) Hatchery_n:Farm_n:Hatchery_29:Farm_13] -0.1 0.6 -1.4 1.0

b[(Intercept) Hatchery_n:Farm_n:Hatchery_29:Farm_6] 0.2 0.5 -0.7 1.4

b[(Intercept) Hatchery_n:Farm_n:Hatchery_3:Farm_8] -0.4 0.6 -1.7 0.6

b[(Intercept) Hatchery_n:Farm_n:Hatchery_30:Farm_13] -0.2 0.6 -1.4 0.8

b[(Intercept) Hatchery_n:Farm_n:Hatchery_31:Farm_10] -0.4 0.6 -1.8 0.6

b[(Intercept) Hatchery_n:Farm_n:Hatchery_31:Farm_11] -0.4 0.6 -1.6 0.7

b[(Intercept) Hatchery_n:Farm_n:Hatchery_31:Farm_15] 0.0 0.5 -1.0 1.0

b[(Intercept) Hatchery_n:Farm_n:Hatchery_32:Farm_8] -0.4 0.6 -1.7 0.7

b[(Intercept) Hatchery_n:Farm_n:Hatchery_33:Farm_13] 0.7 0.6 -0.3 2.1

b[(Intercept) Hatchery_n:Farm_n:Hatchery_34:Farm_7] -0.1 0.6 -1.2 1.0

b[(Intercept) Hatchery_n:Farm_n:Hatchery_34:Farm_8] 1.2 0.7 0.0 2.7

b[(Intercept) Hatchery_n:Farm_n:Hatchery_35:Farm_9] -0.1 0.6 -1.4 1.1

b[(Intercept) Hatchery_n:Farm_n:Hatchery_36:Farm_12] 0.0 0.6 -1.3 1.2

b[(Intercept) Hatchery_n:Farm_n:Hatchery_37:Farm_19] -0.1 0.6 -1.3 1.1

b[(Intercept) Hatchery_n:Farm_n:Hatchery_38:Farm_14] 0.1 0.5 -0.8 1.0

b[(Intercept) Hatchery_n:Farm_n:Hatchery_39:Farm_2] -0.4 0.6 -1.7 0.6

b[(Intercept) Hatchery_n:Farm_n:Hatchery_40:Farm_1] -0.4 0.5 -1.6 0.5

b[(Intercept) Hatchery_n:Farm_n:Hatchery_40:Farm_10] 0.2 0.4 -0.7 1.1

b[(Intercept) Hatchery_n:Farm_n:Hatchery_40:Farm_11] 0.4 0.5 -0.4 1.5

b[(Intercept) Hatchery_n:Farm_n:Hatchery_40:Farm_16] 0.0 0.4 -0.9 0.8

b[(Intercept) Hatchery_n:Farm_n:Hatchery_40:Farm_17] -0.3 0.5 -1.3 0.6

b[(Intercept) Hatchery_n:Farm_n:Hatchery_40:Farm_9] -0.1 0.6 -1.4 1.1

b[(Intercept) Hatchery_n:Farm_n:Hatchery_41:Farm_16] 0.1 0.5 -0.9 1.1

b[(Intercept) Hatchery_n:Farm_n:Hatchery_42:Farm_11] 0.0 0.5 -0.9 1.0

b[(Intercept) Hatchery_n:Farm_n:Hatchery_43:Farm_11] 0.3 0.5 -0.6 1.5

b[(Intercept) Hatchery_n:Farm_n:Hatchery_45:Farm_2] -0.2 0.5 -1.4 0.8

b[(Intercept) Hatchery_n:Farm_n:Hatchery_46:Farm_19] -0.1 0.6 -1.4 1.0

b[(Intercept) Hatchery_n:Farm_n:Hatchery_47:Farm_18] -0.1 0.6 -1.4 1.0

b[(Intercept) Hatchery_n:Farm_n:Hatchery_48:Farm_18] -0.1 0.6 -1.4 1.0

b[(Intercept) Hatchery_n:Farm_n:Hatchery_49:Farm_16] 0.0 0.5 -1.0 1.0

b[(Intercept) Hatchery_n:Farm_n:Hatchery_5:Farm_18] -0.1 0.6 -1.4 1.0

b[(Intercept) Hatchery_n:Farm_n:Hatchery_5:Farm_19] 0.5 0.6 -0.5 1.9

b[(Intercept) Hatchery_n:Farm_n:Hatchery_50:Farm_4] 0.1 0.4 -0.7 1.0

b[(Intercept) Hatchery_n:Farm_n:Hatchery_51:Farm_14] 0.1 0.5 -0.8 1.1

b[(Intercept) Hatchery_n:Farm_n:Hatchery_51:Farm_18] 0.2 0.5 -0.8 1.4

b[(Intercept) Hatchery_n:Farm_n:Hatchery_52:Farm_17] 0.1 0.5 -0.9 1.1

b[(Intercept) Hatchery_n:Farm_n:Hatchery_53:Farm_19] -0.2 0.6 -1.5 0.9

b[(Intercept) Hatchery_n:Farm_n:Hatchery_54:Farm_14] -0.1 0.5 -1.1 0.8

b[(Intercept) Hatchery_n:Farm_n:Hatchery_55:Farm_16] 0.1 0.5 -0.9 1.2

b[(Intercept) Hatchery_n:Farm_n:Hatchery_56:Farm_10] 0.2 0.5 -0.8 1.3

b[(Intercept) Hatchery_n:Farm_n:Hatchery_57:Farm_19] -0.1 0.6 -1.4 1.1

b[(Intercept) Hatchery_n:Farm_n:Hatchery_58:Farm_3] -0.2 0.5 -1.2 0.9

b[(Intercept) Hatchery_n:Farm_n:Hatchery_59:Farm_16] 0.0 0.5 -1.0 1.0

b[(Intercept) Hatchery_n:Farm_n:Hatchery_59:Farm_17] 0.1 0.5 -0.9 1.1

b[(Intercept) Hatchery_n:Farm_n:Hatchery_6:Farm_4] -0.1 0.5 -1.1 0.9

b[(Intercept) Hatchery_n:Farm_n:Hatchery_60:Farm_17] -0.1 0.5 -1.1 0.9

b[(Intercept) Hatchery_n:Farm_n:Hatchery_60:Farm_9] -0.1 0.6 -1.4 1.2

b[(Intercept) Hatchery_n:Farm_n:Hatchery_61:Farm_5] 0.0 0.6 -1.4 1.2

b[(Intercept) Hatchery_n:Farm_n:Hatchery_62:Farm_17] 0.3 0.5 -0.6 1.4

b[(Intercept) Hatchery_n:Farm_n:Hatchery_63:Farm_1] 0.6 0.6 -0.3 1.8

b[(Intercept) Hatchery_n:Farm_n:Hatchery_63:Farm_4] 0.1 0.5 -0.8 1.2

b[(Intercept) Hatchery_n:Farm_n:Hatchery_64:Farm_5] 0.0 0.6 -1.3 1.1

b[(Intercept) Hatchery_n:Farm_n:Hatchery_64:Farm_6] -0.6 0.6 -1.9 0.3

b[(Intercept) Hatchery_n:Farm_n:Hatchery_65:Farm_14] 0.1 0.5 -0.8 1.0

b[(Intercept) Hatchery_n:Farm_n:Hatchery_66:Farm_2] 0.6 0.6 -0.3 1.8

b[(Intercept) Hatchery_n:Farm_n:Hatchery_67:Farm_12] -0.1 0.6 -1.4 1.1

b[(Intercept) Hatchery_n:Farm_n:Hatchery_67:Farm_9] -0.1 0.6 -1.3 1.1

b[(Intercept) Hatchery_n:Farm_n:Hatchery_68:Farm_12] -0.1 0.6 -1.4 1.1

b[(Intercept) Hatchery_n:Farm_n:Hatchery_69:Farm_12] -0.1 0.6 -1.3 1.2

b[(Intercept) Hatchery_n:Farm_n:Hatchery_7:Farm_13] -0.1 0.6 -1.4 1.0

b[(Intercept) Hatchery_n:Farm_n:Hatchery_8:Farm_7] -0.3 0.6 -1.6 0.7

b[(Intercept) Hatchery_n:Farm_n:Hatchery_9:Farm_19] -0.1 0.6 -1.4 1.1

b[(Intercept) Farm_n:Farm_1] 0.8 0.7 -0.5 2.1

b[(Intercept) Farm_n:Farm_10] 0.8 0.6 -0.3 2.0

b[(Intercept) Farm_n:Farm_11] 0.5 0.6 -0.7 1.7

b[(Intercept) Farm_n:Farm_12] -2.7 1.1 -5.1 -0.9

b[(Intercept) Farm_n:Farm_13] -1.1 0.7 -2.5 0.2

b[(Intercept) Farm_n:Farm_14] 2.0 0.5 1.0 3.2

b[(Intercept) Farm_n:Farm_15] 0.9 0.6 -0.2 2.2

b[(Intercept) Farm_n:Farm_16] 1.0 0.6 -0.1 2.2

b[(Intercept) Farm_n:Farm_17] 0.7 0.6 -0.4 2.0

b[(Intercept) Farm_n:Farm_18] -1.1 0.7 -2.4 0.2

b[(Intercept) Farm_n:Farm_19] -1.4 0.7 -2.9 0.0

b[(Intercept) Farm_n:Farm_2] 0.7 0.6 -0.5 2.0

b[(Intercept) Farm_n:Farm_3] 1.7 0.6 0.6 2.9

b[(Intercept) Farm_n:Farm_4] 1.5 0.6 0.4 2.6

b[(Intercept) Farm_n:Farm_5] -2.7 1.1 -5.2 -0.9

b[(Intercept) Farm_n:Farm_6] 0.7 0.6 -0.4 1.9

b[(Intercept) Farm_n:Farm_7] 0.2 0.7 -1.2 1.5

b[(Intercept) Farm_n:Farm_8] -0.3 0.6 -1.6 0.9

b[(Intercept) Farm_n:Farm_9] -2.3 1.1 -4.8 -0.4

reciprocal_dispersion 3.9 1.5 1.7 7.4

Sigma[Hatchery_n:Farm_n:(Intercept),(Intercept)] 0.4 0.3 0.0 1.0

Sigma[Farm_n:(Intercept),(Intercept)] 2.9 1.5 1.1 6.7

Fit Diagnostics:

mean sd 2.5% 97.5%

mean_PPD 2.8 0.4 2.2 3.5

The mean_ppd is the sample average posterior predictive distribution of the outcome variable (for details see help('summary.stanreg')).

MCMC diagnostics

mcse Rhat n_eff

(Intercept) 0.0 1.0 3448

factor(Cycle)2 0.0 1.0 8432

factor(Cycle)3 0.0 1.0 8244

factor(Cycle)4 0.0 1.0 9466

factor(Cycle)5 0.0 1.0 9129

factor(Cycle)6 0.0 1.0 10037

factor(Season)1 0.0 1.0 7558

N_Broilers 0.0 1.0 7430

firstweekmort 0.0 1.0 6720

Ext_Bio 0.0 1.0 5832

Emp_Eq 0.0 1.0 6412

F_W_Mng 0.0 1.0 6575

Int_Bio 0.0 1.0 8671

Mng_Vet_D 0.0 1.0 7259

Mov_Pur 0.0 1.0 7390

factor(Phase)Pre 0.0 1.0 8791

b[(Intercept) Hatchery_n:Farm_n:Hatchery_1:Farm_18] 0.0 1.0 11793

b[(Intercept) Hatchery_n:Farm_n:Hatchery_1:Farm_19] 0.0 1.0 14441

b[(Intercept) Hatchery_n:Farm_n:Hatchery_10:Farm_1] 0.0 1.0 12363

b[(Intercept) Hatchery_n:Farm_n:Hatchery_10:Farm_2] 0.0 1.0 7084

b[(Intercept) Hatchery_n:Farm_n:Hatchery_11:Farm_3] 0.0 1.0 11766

b[(Intercept) Hatchery_n:Farm_n:Hatchery_12:Farm_10] 0.0 1.0 9701

b[(Intercept) Hatchery_n:Farm_n:Hatchery_13:Farm_13] 0.0 1.0 11962

b[(Intercept) Hatchery_n:Farm_n:Hatchery_14:Farm_11] 0.0 1.0 6504

b[(Intercept) Hatchery_n:Farm_n:Hatchery_15:Farm_13] 0.0 1.0 14997

b[(Intercept) Hatchery_n:Farm_n:Hatchery_16:Farm_14] 0.0 1.0 11995

b[(Intercept) Hatchery_n:Farm_n:Hatchery_17:Farm_15] 0.0 1.0 11656

b[(Intercept) Hatchery_n:Farm_n:Hatchery_18:Farm_3] 0.0 1.0 11555

b[(Intercept) Hatchery_n:Farm_n:Hatchery_18:Farm_4] 0.0 1.0 10576

b[(Intercept) Hatchery_n:Farm_n:Hatchery_19:Farm_3] 0.0 1.0 8153

b[(Intercept) Hatchery_n:Farm_n:Hatchery_2:Farm_15] 0.0 1.0 12015

b[(Intercept) Hatchery_n:Farm_n:Hatchery_2:Farm_18] 0.0 1.0 11822

b[(Intercept) Hatchery_n:Farm_n:Hatchery_2:Farm_7] 0.0 1.0 6182

b[(Intercept) Hatchery_n:Farm_n:Hatchery_2:Farm_8] 0.0 1.0 6016

b[(Intercept) Hatchery_n:Farm_n:Hatchery_2:Farm_9] 0.0 1.0 13539

b[(Intercept) Hatchery_n:Farm_n:Hatchery_20:Farm_12] 0.0 1.0 14919

b[(Intercept) Hatchery_n:Farm_n:Hatchery_21:Farm_4] 0.0 1.0 12127

b[(Intercept) Hatchery_n:Farm_n:Hatchery_22:Farm_14] 0.0 1.0 9492

b[(Intercept) Hatchery_n:Farm_n:Hatchery_22:Farm_15] 0.0 1.0 14308

b[(Intercept) Hatchery_n:Farm_n:Hatchery_22:Farm_2] 0.0 1.0 4260

b[(Intercept) Hatchery_n:Farm_n:Hatchery_22:Farm_5] 0.0 1.0 13638

b[(Intercept) Hatchery_n:Farm_n:Hatchery_22:Farm_6] 0.0 1.0 6545

b[(Intercept) Hatchery_n:Farm_n:Hatchery_23:Farm_5] 0.0 1.0 15119

b[(Intercept) Hatchery_n:Farm_n:Hatchery_24:Farm_6] 0.0 1.0 8825

b[(Intercept) Hatchery_n:Farm_n:Hatchery_25:Farm_6] 0.0 1.0 3027

b[(Intercept) Hatchery_n:Farm_n:Hatchery_26:Farm_5] 0.0 1.0 14257

b[(Intercept) Hatchery_n:Farm_n:Hatchery_27:Farm_5] 0.0 1.0 13973

b[(Intercept) Hatchery_n:Farm_n:Hatchery_28:Farm_13] 0.0 1.0 12865

b[(Intercept) Hatchery_n:Farm_n:Hatchery_29:Farm_12] 0.0 1.0 15056

b[(Intercept) Hatchery_n:Farm_n:Hatchery_29:Farm_13] 0.0 1.0 12410

b[(Intercept) Hatchery_n:Farm_n:Hatchery_29:Farm_6] 0.0 1.0 7756

b[(Intercept) Hatchery_n:Farm_n:Hatchery_3:Farm_8] 0.0 1.0 6905

b[(Intercept) Hatchery_n:Farm_n:Hatchery_30:Farm_13] 0.0 1.0 11667

b[(Intercept) Hatchery_n:Farm_n:Hatchery_31:Farm_10] 0.0 1.0 5889

b[(Intercept) Hatchery_n:Farm_n:Hatchery_31:Farm_11] 0.0 1.0 6663

b[(Intercept) Hatchery_n:Farm_n:Hatchery_31:Farm_15] 0.0 1.0 13086

b[(Intercept) Hatchery_n:Farm_n:Hatchery_32:Farm_8] 0.0 1.0 6827

b[(Intercept) Hatchery_n:Farm_n:Hatchery_33:Farm_13] 0.0 1.0 3138

b[(Intercept) Hatchery_n:Farm_n:Hatchery_34:Farm_7] 0.0 1.0 13508

b[(Intercept) Hatchery_n:Farm_n:Hatchery_34:Farm_8] 0.0 1.0 1866

b[(Intercept) Hatchery_n:Farm_n:Hatchery_35:Farm_9] 0.0 1.0 13204

b[(Intercept) Hatchery_n:Farm_n:Hatchery_36:Farm_12] 0.0 1.0 13621

b[(Intercept) Hatchery_n:Farm_n:Hatchery_37:Farm_19] 0.0 1.0 13444

b[(Intercept) Hatchery_n:Farm_n:Hatchery_38:Farm_14] 0.0 1.0 11894

b[(Intercept) Hatchery_n:Farm_n:Hatchery_39:Farm_2] 0.0 1.0 5791

b[(Intercept) Hatchery_n:Farm_n:Hatchery_40:Farm_1] 0.0 1.0 6278

b[(Intercept) Hatchery_n:Farm_n:Hatchery_40:Farm_10] 0.0 1.0 8890

b[(Intercept) Hatchery_n:Farm_n:Hatchery_40:Farm_11] 0.0 1.0 4711

b[(Intercept) Hatchery_n:Farm_n:Hatchery_40:Farm_16] 0.0 1.0 10533

b[(Intercept) Hatchery_n:Farm_n:Hatchery_40:Farm_17] 0.0 1.0 9646

b[(Intercept) Hatchery_n:Farm_n:Hatchery_40:Farm_9] 0.0 1.0 14671

b[(Intercept) Hatchery_n:Farm_n:Hatchery_41:Farm_16] 0.0 1.0 10520

b[(Intercept) Hatchery_n:Farm_n:Hatchery_42:Farm_11] 0.0 1.0 11379

b[(Intercept) Hatchery_n:Farm_n:Hatchery_43:Farm_11] 0.0 1.0 7671

b[(Intercept) Hatchery_n:Farm_n:Hatchery_45:Farm_2] 0.0 1.0 11653

b[(Intercept) Hatchery_n:Farm_n:Hatchery_46:Farm_19] 0.0 1.0 13913

b[(Intercept) Hatchery_n:Farm_n:Hatchery_47:Farm_18] 0.0 1.0 14188

b[(Intercept) Hatchery_n:Farm_n:Hatchery_48:Farm_18] 0.0 1.0 10900

b[(Intercept) Hatchery_n:Farm_n:Hatchery_49:Farm_16] 0.0 1.0 13356

b[(Intercept) Hatchery_n:Farm_n:Hatchery_5:Farm_18] 0.0 1.0 12563

b[(Intercept) Hatchery_n:Farm_n:Hatchery_5:Farm_19] 0.0 1.0 4401

b[(Intercept) Hatchery_n:Farm_n:Hatchery_50:Farm_4] 0.0 1.0 10478

b[(Intercept) Hatchery_n:Farm_n:Hatchery_51:Farm_14] 0.0 1.0 11883

b[(Intercept) Hatchery_n:Farm_n:Hatchery_51:Farm_18] 0.0 1.0 10053

b[(Intercept) Hatchery_n:Farm_n:Hatchery_52:Farm_17] 0.0 1.0 13528

b[(Intercept) Hatchery_n:Farm_n:Hatchery_53:Farm_19] 0.0 1.0 11239

b[(Intercept) Hatchery_n:Farm_n:Hatchery_54:Farm_14] 0.0 1.0 11476

b[(Intercept) Hatchery_n:Farm_n:Hatchery_55:Farm_16] 0.0 1.0 10851

b[(Intercept) Hatchery_n:Farm_n:Hatchery_56:Farm_10] 0.0 1.0 10218

b[(Intercept) Hatchery_n:Farm_n:Hatchery_57:Farm_19] 0.0 1.0 15144

b[(Intercept) Hatchery_n:Farm_n:Hatchery_58:Farm_3] 0.0 1.0 10571

b[(Intercept) Hatchery_n:Farm_n:Hatchery_59:Farm_16] 0.0 1.0 13627

b[(Intercept) Hatchery_n:Farm_n:Hatchery_59:Farm_17] 0.0 1.0 13674

b[(Intercept) Hatchery_n:Farm_n:Hatchery_6:Farm_4] 0.0 1.0 12743

b[(Intercept) Hatchery_n:Farm_n:Hatchery_60:Farm_17] 0.0 1.0 12909

b[(Intercept) Hatchery_n:Farm_n:Hatchery_60:Farm_9] 0.0 1.0 15159

b[(Intercept) Hatchery_n:Farm_n:Hatchery_61:Farm_5] 0.0 1.0 15693

b[(Intercept) Hatchery_n:Farm_n:Hatchery_62:Farm_17] 0.0 1.0 8009

b[(Intercept) Hatchery_n:Farm_n:Hatchery_63:Farm_1] 0.0 1.0 3531

b[(Intercept) Hatchery_n:Farm_n:Hatchery_63:Farm_4] 0.0 1.0 11373

b[(Intercept) Hatchery_n:Farm_n:Hatchery_64:Farm_5] 0.0 1.0 13347

b[(Intercept) Hatchery_n:Farm_n:Hatchery_64:Farm_6] 0.0 1.0 4452

b[(Intercept) Hatchery_n:Farm_n:Hatchery_65:Farm_14] 0.0 1.0 12722

b[(Intercept) Hatchery_n:Farm_n:Hatchery_66:Farm_2] 0.0 1.0 3510

b[(Intercept) Hatchery_n:Farm_n:Hatchery_67:Farm_12] 0.0 1.0 14835

b[(Intercept) Hatchery_n:Farm_n:Hatchery_67:Farm_9] 0.0 1.0 15381

b[(Intercept) Hatchery_n:Farm_n:Hatchery_68:Farm_12] 0.0 1.0 16564

b[(Intercept) Hatchery_n:Farm_n:Hatchery_69:Farm_12] 0.0 1.0 15325

b[(Intercept) Hatchery_n:Farm_n:Hatchery_7:Farm_13] 0.0 1.0 12697

b[(Intercept) Hatchery_n:Farm_n:Hatchery_8:Farm_7] 0.0 1.0 8335

b[(Intercept) Hatchery_n:Farm_n:Hatchery_9:Farm_19] 0.0 1.0 13415

b[(Intercept) Farm_n:Farm_1] 0.0 1.0 4577

b[(Intercept) Farm_n:Farm_10] 0.0 1.0 4515

b[(Intercept) Farm_n:Farm_11] 0.0 1.0 4383

b[(Intercept) Farm_n:Farm_12] 0.0 1.0 7462

b[(Intercept) Farm_n:Farm_13] 0.0 1.0 5177

b[(Intercept) Farm_n:Farm_14] 0.0 1.0 3973

b[(Intercept) Farm_n:Farm_15] 0.0 1.0 4390

b[(Intercept) Farm_n:Farm_16] 0.0 1.0 4279

b[(Intercept) Farm_n:Farm_17] 0.0 1.0 4062

b[(Intercept) Farm_n:Farm_18] 0.0 1.0 5021

b[(Intercept) Farm_n:Farm_19] 0.0 1.0 6043

b[(Intercept) Farm_n:Farm_2] 0.0 1.0 4581

b[(Intercept) Farm_n:Farm_3] 0.0 1.0 4483

b[(Intercept) Farm_n:Farm_4] 0.0 1.0 4154

b[(Intercept) Farm_n:Farm_5] 0.0 1.0 7629

b[(Intercept) Farm_n:Farm_6] 0.0 1.0 3842

b[(Intercept) Farm_n:Farm_7] 0.0 1.0 5381

b[(Intercept) Farm_n:Farm_8] 0.0 1.0 5221

b[(Intercept) Farm_n:Farm_9] 0.0 1.0 7133

reciprocal_dispersion 0.0 1.0 3175

Sigma[Hatchery_n:Farm_n:(Intercept),(Intercept)] 0.0 1.0 1248

Sigma[Farm_n:(Intercept),(Intercept)] 0.0 1.0 3631

mean_PPD 0.0 1.0 7244

log-posterior 0.4 1.0 1111

For each parameter, mcse is Monte Carlo standard error, n_eff is a crude measure of effective sample size, and Rhat is the potential scale reduction factor on split chains (at convergence Rhat=1).

# ***Macrolide hierarchical Bayesian negative-binomial regression model***

Relevant for model fit: the Monte Carlo Standard Error (MCSE) for each parameter was small, indicating high precision in the posterior mean estimates. The potential scale reduction factor (Rhat) values were close to 1.00, suggesting that the model had likely converged effectively. Additionally, the effective sample size (n_eff) was sufficiently large, reflecting a high number of independent samples and providing confidence in the reliability of the parameter estimates.

> summary(fit.mac,probs=c(0.025,0.975))

Model Info:

function: stan_glmer

family: neg_binomial_2 [log]

formula: MAC_count ~ factor(Cycle) + factor(Season) + N_Broilers + firstweekmort +

Purpose + Ext_Bio + Emp_Eq + F_W_Mng + Int_Bio + Mng_Vet_D +

Mov_Pur + factor(Phase) + (1 | Farm_n/Hatchery_n)

algorithm: sampling

sample: 8000 (posterior sample size)

priors: see help('prior_summary')

observations: 142

groups: Hatchery_n:Farm_n (95), Farm_n (19)

Estimates:

mean sd 2.5% 97.5%

(Intercept) 0.3 0.5 -0.7 1.2

factor(Cycle)2 0.0 0.1 -0.1 0.2

factor(Cycle)3 0.0 0.1 -0.1 0.2

factor(Cycle)4 0.0 0.1 -0.3 0.1

factor(Cycle)5 0.0 0.1 -0.2 0.2

factor(Cycle)6 0.0 0.2 -0.5 0.4

factor(Season)1 0.0 0.1 -0.2 0.1

N_Broilers 0.0 0.0 0.0 0.0

firstweekmort 0.0 0.0 -0.1 0.0

Purpose 0.0 0.1 -0.3 0.2

Ext_Bio 0.1 0.3 -0.2 0.8

Emp_Eq 0.0 0.2 -0.3 0.4

F_W_Mng 0.0 0.3 -0.5 0.8

Int_Bio 0.0 0.4 -0.6 0.9

Mng_Vet_D 0.0 0.1 -0.2 0.3

Mov_Pur 0.0 0.2 -0.4 0.3

factor(Phase)Pre 0.0 0.1 -0.2 0.1

b[(Intercept) Hatchery_n:Farm_n:Hatchery_1:Farm_18] -0.3 0.5 -1.7 0.5

b[(Intercept) Hatchery_n:Farm_n:Hatchery_1:Farm_19] -0.1 0.5 -1.3 0.8

b[(Intercept) Hatchery_n:Farm_n:Hatchery_10:Farm_1] -0.2 0.5 -1.4 0.8

b[(Intercept) Hatchery_n:Farm_n:Hatchery_10:Farm_2] -0.1 0.5 -1.3 1.0

b[(Intercept) Hatchery_n:Farm_n:Hatchery_11:Farm_3] 0.1 0.4 -0.8 1.1

b[(Intercept) Hatchery_n:Farm_n:Hatchery_12:Farm_10] 0.1 0.5 -0.7 1.3

b[(Intercept) Hatchery_n:Farm_n:Hatchery_13:Farm_13] -0.2 0.5 -1.5 0.7

b[(Intercept) Hatchery_n:Farm_n:Hatchery_14:Farm_11] -0.2 0.5 -1.6 0.7

b[(Intercept) Hatchery_n:Farm_n:Hatchery_15:Farm_13] 0.3 0.5 -0.5 1.6

b[(Intercept) Hatchery_n:Farm_n:Hatchery_16:Farm_14] 0.1 0.4 -0.7 1.1

b[(Intercept) Hatchery_n:Farm_n:Hatchery_17:Farm_15] 0.0 0.4 -0.9 0.9

b[(Intercept) Hatchery_n:Farm_n:Hatchery_18:Farm_3] -0.1 0.4 -1.0 0.8

b[(Intercept) Hatchery_n:Farm_n:Hatchery_18:Farm_4] 0.1 0.4 -0.8 1.0

b[(Intercept) Hatchery_n:Farm_n:Hatchery_19:Farm_3] 0.1 0.4 -0.7 1.2

b[(Intercept) Hatchery_n:Farm_n:Hatchery_2:Farm_15] 0.1 0.5 -0.8 1.1

b[(Intercept) Hatchery_n:Farm_n:Hatchery_2:Farm_18] -0.1 0.5 -1.4 0.8

b[(Intercept) Hatchery_n:Farm_n:Hatchery_2:Farm_7] -0.1 0.5 -1.1 0.9

b[(Intercept) Hatchery_n:Farm_n:Hatchery_2:Farm_8] -0.2 0.5 -1.5 0.9

b[(Intercept) Hatchery_n:Farm_n:Hatchery_2:Farm_9] 0.1 0.4 -0.8 1.1

b[(Intercept) Hatchery_n:Farm_n:Hatchery_20:Farm_12] -0.1 0.5 -1.3 0.9

b[(Intercept) Hatchery_n:Farm_n:Hatchery_21:Farm_4] -0.2 0.5 -1.6 0.7

b[(Intercept) Hatchery_n:Farm_n:Hatchery_22:Farm_14] 0.2 0.4 -0.5 1.1

b[(Intercept) Hatchery_n:Farm_n:Hatchery_22:Farm_15] 0.0 0.5 -0.9 1.1

b[(Intercept) Hatchery_n:Farm_n:Hatchery_22:Farm_2] -0.1 0.5 -1.3 1.0

b[(Intercept) Hatchery_n:Farm_n:Hatchery_22:Farm_5] -0.2 0.5 -1.5 0.6

b[(Intercept) Hatchery_n:Farm_n:Hatchery_22:Farm_6] 0.1 0.5 -0.7 1.2

b[(Intercept) Hatchery_n:Farm_n:Hatchery_23:Farm_5] 0.2 0.5 -0.7 1.4

b[(Intercept) Hatchery_n:Farm_n:Hatchery_24:Farm_6] -0.3 0.5 -1.6 0.6

b[(Intercept) Hatchery_n:Farm_n:Hatchery_25:Farm_6] 0.6 0.6 -0.2 1.9

b[(Intercept) Hatchery_n:Farm_n:Hatchery_26:Farm_5] -0.2 0.5 -1.5 0.6

b[(Intercept) Hatchery_n:Farm_n:Hatchery_27:Farm_5] -0.2 0.5 -1.6 0.7

b[(Intercept) Hatchery_n:Farm_n:Hatchery_28:Farm_13] 0.3 0.5 -0.5 1.6

b[(Intercept) Hatchery_n:Farm_n:Hatchery_29:Farm_12] -0.1 0.5 -1.4 0.8

b[(Intercept) Hatchery_n:Farm_n:Hatchery_29:Farm_13] -0.2 0.5 -1.4 0.7

b[(Intercept) Hatchery_n:Farm_n:Hatchery_29:Farm_6] 0.1 0.5 -0.7 1.2

b[(Intercept) Hatchery_n:Farm_n:Hatchery_3:Farm_8] -0.1 0.5 -1.4 0.9

b[(Intercept) Hatchery_n:Farm_n:Hatchery_30:Farm_13] -0.3 0.5 -1.7 0.5

b[(Intercept) Hatchery_n:Farm_n:Hatchery_31:Farm_10] -0.2 0.5 -1.6 0.6

b[(Intercept) Hatchery_n:Farm_n:Hatchery_31:Farm_11] -0.2 0.5 -1.6 0.7

b[(Intercept) Hatchery_n:Farm_n:Hatchery_31:Farm_15] 0.0 0.5 -0.9 1.1

b[(Intercept) Hatchery_n:Farm_n:Hatchery_32:Farm_8] -0.1 0.5 -1.4 0.9

b[(Intercept) Hatchery_n:Farm_n:Hatchery_33:Farm_13] 0.2 0.4 -0.6 1.2

b[(Intercept) Hatchery_n:Farm_n:Hatchery_34:Farm_7] 0.2 0.5 -0.7 1.4

b[(Intercept) Hatchery_n:Farm_n:Hatchery_34:Farm_8] -0.1 0.5 -1.3 1.0

b[(Intercept) Hatchery_n:Farm_n:Hatchery_35:Farm_9] 0.1 0.4 -0.7 1.2

b[(Intercept) Hatchery_n:Farm_n:Hatchery_36:Farm_12] -0.1 0.5 -1.3 0.9

b[(Intercept) Hatchery_n:Farm_n:Hatchery_37:Farm_19] 0.3 0.5 -0.5 1.6

b[(Intercept) Hatchery_n:Farm_n:Hatchery_38:Farm_14] 0.1 0.4 -0.7 1.1

b[(Intercept) Hatchery_n:Farm_n:Hatchery_39:Farm_2] -0.1 0.5 -1.3 1.0

b[(Intercept) Hatchery_n:Farm_n:Hatchery_40:Farm_1] 0.1 0.4 -0.7 1.1

b[(Intercept) Hatchery_n:Farm_n:Hatchery_40:Farm_10] 0.1 0.4 -0.7 1.0

b[(Intercept) Hatchery_n:Farm_n:Hatchery_40:Farm_11] 0.2 0.4 -0.5 1.3

b[(Intercept) Hatchery_n:Farm_n:Hatchery_40:Farm_16] 0.0 0.4 -0.8 0.9

b[(Intercept) Hatchery_n:Farm_n:Hatchery_40:Farm_17] -0.1 0.4 -0.9 0.8

b[(Intercept) Hatchery_n:Farm_n:Hatchery_40:Farm_9] 0.1 0.4 -0.7 1.2

b[(Intercept) Hatchery_n:Farm_n:Hatchery_41:Farm_16] -0.2 0.5 -1.6 0.7

b[(Intercept) Hatchery_n:Farm_n:Hatchery_42:Farm_11] 0.2 0.4 -0.5 1.3

b[(Intercept) Hatchery_n:Farm_n:Hatchery_43:Farm_11] 0.2 0.5 -0.7 1.3

b[(Intercept) Hatchery_n:Farm_n:Hatchery_45:Farm_2] -0.1 0.5 -1.2 1.0

b[(Intercept) Hatchery_n:Farm_n:Hatchery_46:Farm_19] 0.4 0.6 -0.4 1.8

b[(Intercept) Hatchery_n:Farm_n:Hatchery_47:Farm_18] -0.1 0.5 -1.4 0.8

b[(Intercept) Hatchery_n:Farm_n:Hatchery_48:Farm_18] 0.3 0.5 -0.5 1.6

b[(Intercept) Hatchery_n:Farm_n:Hatchery_49:Farm_16] -0.2 0.5 -1.5 0.7

b[(Intercept) Hatchery_n:Farm_n:Hatchery_5:Farm_18] -0.1 0.5 -1.4 0.8

b[(Intercept) Hatchery_n:Farm_n:Hatchery_5:Farm_19] -0.2 0.5 -1.6 0.7

b[(Intercept) Hatchery_n:Farm_n:Hatchery_50:Farm_4] 0.3 0.5 -0.4 1.4

b[(Intercept) Hatchery_n:Farm_n:Hatchery_51:Farm_14] 0.1 0.4 -0.7 1.1

b[(Intercept) Hatchery_n:Farm_n:Hatchery_51:Farm_18] 0.3 0.5 -0.5 1.7

b[(Intercept) Hatchery_n:Farm_n:Hatchery_52:Farm_17] -0.1 0.5 -1.2 0.8

b[(Intercept) Hatchery_n:Farm_n:Hatchery_53:Farm_19] -0.2 0.5 -1.5 0.6

b[(Intercept) Hatchery_n:Farm_n:Hatchery_54:Farm_14] -0.3 0.6 -1.9 0.5

b[(Intercept) Hatchery_n:Farm_n:Hatchery_55:Farm_16] 0.3 0.5 -0.5 1.5

b[(Intercept) Hatchery_n:Farm_n:Hatchery_56:Farm_10] 0.1 0.5 -0.7 1.3

b[(Intercept) Hatchery_n:Farm_n:Hatchery_57:Farm_19] -0.1 0.5 -1.3 0.9

b[(Intercept) Hatchery_n:Farm_n:Hatchery_58:Farm_3] 0.1 0.5 -0.8 1.1

b[(Intercept) Hatchery_n:Farm_n:Hatchery_59:Farm_16] 0.2 0.5 -0.6 1.4

b[(Intercept) Hatchery_n:Farm_n:Hatchery_59:Farm_17] 0.0 0.5 -1.0 1.0

b[(Intercept) Hatchery_n:Farm_n:Hatchery_6:Farm_4] -0.2 0.5 -1.5 0.7

b[(Intercept) Hatchery_n:Farm_n:Hatchery_60:Farm_17] -0.1 0.5 -1.2 0.9

b[(Intercept) Hatchery_n:Farm_n:Hatchery_60:Farm_9] -0.1 0.5 -1.1 0.8

b[(Intercept) Hatchery_n:Farm_n:Hatchery_61:Farm_5] 0.4 0.6 -0.3 1.9

b[(Intercept) Hatchery_n:Farm_n:Hatchery_62:Farm_17] 0.4 0.5 -0.4 1.6

b[(Intercept) Hatchery_n:Farm_n:Hatchery_63:Farm_1] 0.3 0.5 -0.5 1.5

b[(Intercept) Hatchery_n:Farm_n:Hatchery_63:Farm_4] 0.1 0.5 -0.7 1.2

b[(Intercept) Hatchery_n:Farm_n:Hatchery_64:Farm_5] -0.1 0.5 -1.4 0.8

b[(Intercept) Hatchery_n:Farm_n:Hatchery_64:Farm_6] -0.4 0.6 -1.8 0.4

b[(Intercept) Hatchery_n:Farm_n:Hatchery_65:Farm_14] 0.1 0.4 -0.7 1.2

b[(Intercept) Hatchery_n:Farm_n:Hatchery_66:Farm_2] -0.1 0.5 -1.3 1.0

b[(Intercept) Hatchery_n:Farm_n:Hatchery_67:Farm_12] 0.2 0.5 -0.7 1.5

b[(Intercept) Hatchery_n:Farm_n:Hatchery_67:Farm_9] 0.1 0.4 -0.7 1.2

b[(Intercept) Hatchery_n:Farm_n:Hatchery_68:Farm_12] -0.2 0.5 -1.4 0.8

b[(Intercept) Hatchery_n:Farm_n:Hatchery_69:Farm_12] -0.1 0.5 -1.3 0.9

b[(Intercept) Hatchery_n:Farm_n:Hatchery_7:Farm_13] -0.2 0.5 -1.4 0.7

b[(Intercept) Hatchery_n:Farm_n:Hatchery_8:Farm_7] -0.2 0.5 -1.5 0.8

b[(Intercept) Hatchery_n:Farm_n:Hatchery_9:Farm_19] -0.1 0.5 -1.4 0.8

b[(Intercept) Farm_n:Farm_1] 0.9 0.6 -0.2 2.1

b[(Intercept) Farm_n:Farm_10] 0.5 0.5 -0.6 1.5

b[(Intercept) Farm_n:Farm_11] 0.5 0.5 -0.5 1.5

b[(Intercept) Farm_n:Farm_12] -1.4 0.7 -2.9 -0.2

b[(Intercept) Farm_n:Farm_13] -0.2 0.5 -1.2 0.8

b[(Intercept) Farm_n:Farm_14] 1.3 0.5 0.4 2.3

b[(Intercept) Farm_n:Farm_15] 0.5 0.5 -0.5 1.7

b[(Intercept) Farm_n:Farm_16] 0.2 0.5 -0.8 1.2

b[(Intercept) Farm_n:Farm_17] 0.4 0.5 -0.6 1.5

b[(Intercept) Farm_n:Farm_18] -0.6 0.6 -1.8 0.5

b[(Intercept) Farm_n:Farm_19] -0.7 0.5 -1.8 0.4

b[(Intercept) Farm_n:Farm_2] -1.7 0.9 -3.7 -0.1

b[(Intercept) Farm_n:Farm_3] 0.8 0.6 -0.3 1.9

b[(Intercept) Farm_n:Farm_4] 0.2 0.5 -0.8 1.2

b[(Intercept) Farm_n:Farm_5] -0.7 0.6 -1.9 0.3

b[(Intercept) Farm_n:Farm_6] 0.7 0.5 -0.3 1.7

b[(Intercept) Farm_n:Farm_7] -0.1 0.6 -1.4 1.1

b[(Intercept) Farm_n:Farm_8] -2.0 0.9 -4.0 -0.5

b[(Intercept) Farm_n:Farm_9] 1.5 0.5 0.5 2.6

reciprocal_dispersion 1.9 0.9 0.8 4.2

Sigma[Hatchery_n:Farm_n:(Intercept),(Intercept)] 0.3 0.3 0.0 1.1

Sigma[Farm_n:(Intercept),(Intercept)] 1.4 0.8 0.5 3.5

Fit Diagnostics:

mean sd 2.5% 97.5%

mean_PPD 2.6 0.4 1.9 3.5

The mean_ppd is the sample average posterior predictive distribution of the outcome variable (for details see help('summary.stanreg')).

MCMC diagnostics

mcse Rhat n_eff

(Intercept) 0.0 1.0 3658

factor(Cycle)2 0.0 1.0 8055

factor(Cycle)3 0.0 1.0 8882

factor(Cycle)4 0.0 1.0 7879

factor(Cycle)5 0.0 1.0 9031

factor(Cycle)6 0.0 1.0 9249

factor(Season)1 0.0 1.0 6693

N_Broilers 0.0 1.0 7803

firstweekmort 0.0 1.0 5891

Purpose 0.0 1.0 8716

Ext_Bio 0.0 1.0 4558

Emp_Eq 0.0 1.0 7924

F_W_Mng 0.0 1.0 5623

Int_Bio 0.0 1.0 5815

Mng_Vet_D 0.0 1.0 6716

Mov_Pur 0.0 1.0 6770

factor(Phase)Pre 0.0 1.0 8324

b[(Intercept) Hatchery_n:Farm_n:Hatchery_1:Farm_18] 0.0 1.0 2865

b[(Intercept) Hatchery_n:Farm_n:Hatchery_1:Farm_19] 0.0 1.0 8080

b[(Intercept) Hatchery_n:Farm_n:Hatchery_10:Farm_1] 0.0 1.0 6092

b[(Intercept) Hatchery_n:Farm_n:Hatchery_10:Farm_2] 0.0 1.0 13097

b[(Intercept) Hatchery_n:Farm_n:Hatchery_11:Farm_3] 0.0 1.0 11535

b[(Intercept) Hatchery_n:Farm_n:Hatchery_12:Farm_10] 0.0 1.0 5124

b[(Intercept) Hatchery_n:Farm_n:Hatchery_13:Farm_13] 0.0 1.0 4987

b[(Intercept) Hatchery_n:Farm_n:Hatchery_14:Farm_11] 0.0 1.0 3571

b[(Intercept) Hatchery_n:Farm_n:Hatchery_15:Farm_13] 0.0 1.0 2198

b[(Intercept) Hatchery_n:Farm_n:Hatchery_16:Farm_14] 0.0 1.0 6930

b[(Intercept) Hatchery_n:Farm_n:Hatchery_17:Farm_15] 0.0 1.0 11662

b[(Intercept) Hatchery_n:Farm_n:Hatchery_18:Farm_3] 0.0 1.0 10692

b[(Intercept) Hatchery_n:Farm_n:Hatchery_18:Farm_4] 0.0 1.0 8528

b[(Intercept) Hatchery_n:Farm_n:Hatchery_19:Farm_3] 0.0 1.0 6844

b[(Intercept) Hatchery_n:Farm_n:Hatchery_2:Farm_15] 0.0 1.0 12343

b[(Intercept) Hatchery_n:Farm_n:Hatchery_2:Farm_18] 0.0 1.0 6340

b[(Intercept) Hatchery_n:Farm_n:Hatchery_2:Farm_7] 0.0 1.0 11849

b[(Intercept) Hatchery_n:Farm_n:Hatchery_2:Farm_8] 0.0 1.0 7100

b[(Intercept) Hatchery_n:Farm_n:Hatchery_2:Farm_9] 0.0 1.0 9620

b[(Intercept) Hatchery_n:Farm_n:Hatchery_20:Farm_12] 0.0 1.0 11425

b[(Intercept) Hatchery_n:Farm_n:Hatchery_21:Farm_4] 0.0 1.0 4645

b[(Intercept) Hatchery_n:Farm_n:Hatchery_22:Farm_14] 0.0 1.0 3321

b[(Intercept) Hatchery_n:Farm_n:Hatchery_22:Farm_15] 0.0 1.0 12163

b[(Intercept) Hatchery_n:Farm_n:Hatchery_22:Farm_2] 0.0 1.0 11123

b[(Intercept) Hatchery_n:Farm_n:Hatchery_22:Farm_5] 0.0 1.0 4361

b[(Intercept) Hatchery_n:Farm_n:Hatchery_22:Farm_6] 0.0 1.0 6003

b[(Intercept) Hatchery_n:Farm_n:Hatchery_23:Farm_5] 0.0 1.0 5107

b[(Intercept) Hatchery_n:Farm_n:Hatchery_24:Farm_6] 0.0 1.0 3318

b[(Intercept) Hatchery_n:Farm_n:Hatchery_25:Farm_6] 0.0 1.0 1294

b[(Intercept) Hatchery_n:Farm_n:Hatchery_26:Farm_5] 0.0 1.0 4609

b[(Intercept) Hatchery_n:Farm_n:Hatchery_27:Farm_5] 0.0 1.0 4012

b[(Intercept) Hatchery_n:Farm_n:Hatchery_28:Farm_13] 0.0 1.0 2523

b[(Intercept) Hatchery_n:Farm_n:Hatchery_29:Farm_12] 0.0 1.0 6619

b[(Intercept) Hatchery_n:Farm_n:Hatchery_29:Farm_13] 0.0 1.0 5553

b[(Intercept) Hatchery_n:Farm_n:Hatchery_29:Farm_6] 0.0 1.0 5463

b[(Intercept) Hatchery_n:Farm_n:Hatchery_3:Farm_8] 0.0 1.0 8758

b[(Intercept) Hatchery_n:Farm_n:Hatchery_30:Farm_13] 0.0 1.0 2924

b[(Intercept) Hatchery_n:Farm_n:Hatchery_31:Farm_10] 0.0 1.0 3026

b[(Intercept) Hatchery_n:Farm_n:Hatchery_31:Farm_11] 0.0 1.0 4225

b[(Intercept) Hatchery_n:Farm_n:Hatchery_31:Farm_15] 0.0 1.0 10902

b[(Intercept) Hatchery_n:Farm_n:Hatchery_32:Farm_8] 0.0 1.0 9020

b[(Intercept) Hatchery_n:Farm_n:Hatchery_33:Farm_13] 0.0 1.0 4590

b[(Intercept) Hatchery_n:Farm_n:Hatchery_34:Farm_7] 0.0 1.0 4159

b[(Intercept) Hatchery_n:Farm_n:Hatchery_34:Farm_8] 0.0 1.0 12676

b[(Intercept) Hatchery_n:Farm_n:Hatchery_35:Farm_9] 0.0 1.0 7126

b[(Intercept) Hatchery_n:Farm_n:Hatchery_36:Farm_12] 0.0 1.0 11465

b[(Intercept) Hatchery_n:Farm_n:Hatchery_37:Farm_19] 0.0 1.0 2391

b[(Intercept) Hatchery_n:Farm_n:Hatchery_38:Farm_14] 0.0 1.0 5727

b[(Intercept) Hatchery_n:Farm_n:Hatchery_39:Farm_2] 0.0 1.0 11356

b[(Intercept) Hatchery_n:Farm_n:Hatchery_40:Farm_1] 0.0 1.0 5540

b[(Intercept) Hatchery_n:Farm_n:Hatchery_40:Farm_10] 0.0 1.0 6999

b[(Intercept) Hatchery_n:Farm_n:Hatchery_40:Farm_11] 0.0 1.0 3154

b[(Intercept) Hatchery_n:Farm_n:Hatchery_40:Farm_16] 0.0 1.0 10288

b[(Intercept) Hatchery_n:Farm_n:Hatchery_40:Farm_17] 0.0 1.0 12133

b[(Intercept) Hatchery_n:Farm_n:Hatchery_40:Farm_9] 0.0 1.0 6929

b[(Intercept) Hatchery_n:Farm_n:Hatchery_41:Farm_16] 0.0 1.0 3678

b[(Intercept) Hatchery_n:Farm_n:Hatchery_42:Farm_11] 0.0 1.0 3002

b[(Intercept) Hatchery_n:Farm_n:Hatchery_43:Farm_11] 0.0 1.0 4861

b[(Intercept) Hatchery_n:Farm_n:Hatchery_45:Farm_2] 0.0 1.0 10851

b[(Intercept) Hatchery_n:Farm_n:Hatchery_46:Farm_19] 0.0 1.0 1738

b[(Intercept) Hatchery_n:Farm_n:Hatchery_47:Farm_18] 0.0 1.0 7029

b[(Intercept) Hatchery_n:Farm_n:Hatchery_48:Farm_18] 0.0 1.0 2510

b[(Intercept) Hatchery_n:Farm_n:Hatchery_49:Farm_16] 0.0 1.0 3844

b[(Intercept) Hatchery_n:Farm_n:Hatchery_5:Farm_18] 0.0 1.0 6457

b[(Intercept) Hatchery_n:Farm_n:Hatchery_5:Farm_19] 0.0 1.0 3814

b[(Intercept) Hatchery_n:Farm_n:Hatchery_50:Farm_4] 0.0 1.0 2633

b[(Intercept) Hatchery_n:Farm_n:Hatchery_51:Farm_14] 0.0 1.0 6064

b[(Intercept) Hatchery_n:Farm_n:Hatchery_51:Farm_18] 0.0 1.0 2722

b[(Intercept) Hatchery_n:Farm_n:Hatchery_52:Farm_17] 0.0 1.0 8648

b[(Intercept) Hatchery_n:Farm_n:Hatchery_53:Farm_19] 0.0 1.0 4109

b[(Intercept) Hatchery_n:Farm_n:Hatchery_54:Farm_14] 0.0 1.0 2317

b[(Intercept) Hatchery_n:Farm_n:Hatchery_55:Farm_16] 0.0 1.0 2738

b[(Intercept) Hatchery_n:Farm_n:Hatchery_56:Farm_10] 0.0 1.0 5537

b[(Intercept) Hatchery_n:Farm_n:Hatchery_57:Farm_19] 0.0 1.0 7759

b[(Intercept) Hatchery_n:Farm_n:Hatchery_58:Farm_3] 0.0 1.0 9397

b[(Intercept) Hatchery_n:Farm_n:Hatchery_59:Farm_16] 0.0 1.0 4464

b[(Intercept) Hatchery_n:Farm_n:Hatchery_59:Farm_17] 0.0 1.0 13883

b[(Intercept) Hatchery_n:Farm_n:Hatchery_6:Farm_4] 0.0 1.0 4326

b[(Intercept) Hatchery_n:Farm_n:Hatchery_60:Farm_17] 0.0 1.0 9461

b[(Intercept) Hatchery_n:Farm_n:Hatchery_60:Farm_9] 0.0 1.0 9779

b[(Intercept) Hatchery_n:Farm_n:Hatchery_61:Farm_5] 0.0 1.0 1702

b[(Intercept) Hatchery_n:Farm_n:Hatchery_62:Farm_17] 0.0 1.0 1999

b[(Intercept) Hatchery_n:Farm_n:Hatchery_63:Farm_1] 0.0 1.0 2742

b[(Intercept) Hatchery_n:Farm_n:Hatchery_63:Farm_4] 0.0 1.0 5761

b[(Intercept) Hatchery_n:Farm_n:Hatchery_64:Farm_5] 0.0 1.0 7500

b[(Intercept) Hatchery_n:Farm_n:Hatchery_64:Farm_6] 0.0 1.0 1814

b[(Intercept) Hatchery_n:Farm_n:Hatchery_65:Farm_14] 0.0 1.0 5477

b[(Intercept) Hatchery_n:Farm_n:Hatchery_66:Farm_2] 0.0 1.0 13039

b[(Intercept) Hatchery_n:Farm_n:Hatchery_67:Farm_12] 0.0 1.0 4030

b[(Intercept) Hatchery_n:Farm_n:Hatchery_67:Farm_9] 0.0 1.0 6344

b[(Intercept) Hatchery_n:Farm_n:Hatchery_68:Farm_12] 0.0 1.0 6675

b[(Intercept) Hatchery_n:Farm_n:Hatchery_69:Farm_12] 0.0 1.0 9331

b[(Intercept) Hatchery_n:Farm_n:Hatchery_7:Farm_13] 0.0 1.0 5519

b[(Intercept) Hatchery_n:Farm_n:Hatchery_8:Farm_7] 0.0 1.0 5034

b[(Intercept) Hatchery_n:Farm_n:Hatchery_9:Farm_19] 0.0 1.0 6807

b[(Intercept) Farm_n:Farm_1] 0.0 1.0 5555

b[(Intercept) Farm_n:Farm_10] 0.0 1.0 4739

b[(Intercept) Farm_n:Farm_11] 0.0 1.0 4573

b[(Intercept) Farm_n:Farm_12] 0.0 1.0 7428

b[(Intercept) Farm_n:Farm_13] 0.0 1.0 5746

b[(Intercept) Farm_n:Farm_14] 0.0 1.0 4497

b[(Intercept) Farm_n:Farm_15] 0.0 1.0 5204

b[(Intercept) Farm_n:Farm_16] 0.0 1.0 5189

b[(Intercept) Farm_n:Farm_17] 0.0 1.0 5593

b[(Intercept) Farm_n:Farm_18] 0.0 1.0 7160

b[(Intercept) Farm_n:Farm_19] 0.0 1.0 6172

b[(Intercept) Farm_n:Farm_2] 0.0 1.0 8459

b[(Intercept) Farm_n:Farm_3] 0.0 1.0 4862

b[(Intercept) Farm_n:Farm_4] 0.0 1.0 5111

b[(Intercept) Farm_n:Farm_5] 0.0 1.0 6588

b[(Intercept) Farm_n:Farm_6] 0.0 1.0 3558

b[(Intercept) Farm_n:Farm_7] 0.0 1.0 7749

b[(Intercept) Farm_n:Farm_8] 0.0 1.0 7169

b[(Intercept) Farm_n:Farm_9] 0.0 1.0 4624

reciprocal_dispersion 0.0 1.0 1329

Sigma[Hatchery_n:Farm_n:(Intercept),(Intercept)] 0.0 1.0 905

Sigma[Farm_n:(Intercept),(Intercept)] 0.0 1.0 3297

mean_PPD 0.0 1.0 8142

log-posterior 0.5 1.0 920

For each parameter, mcse is Monte Carlo standard error, n_eff is a crude measure of effective sample size, and Rhat is the potential scale reduction factor on split chains (at convergence Rhat=1).

# ***Penicillin hierarchical Bayesian negative-binomial regression model***

Relevant for model fit: the Monte Carlo Standard Error (MCSE) for each parameter was small, indicating high precision in the posterior mean estimates. The potential scale reduction factor (Rhat) values were close to 1.00, suggesting that the model had likely converged effectively. Additionally, the effective sample size (n_eff) was sufficiently large, reflecting a high number of independent samples and providing confidence in the reliability of the parameter estimates.

> summary(fit.pen,probs=c(0.025,0.975))

Model Info:

function: stan_glmer

family: neg_binomial_2 [log]

formula: PEN_count ~ factor(Cycle) + factor(Season) + N_Broilers + firstweekmort +

Purpose + Ext_Bio + Emp_Eq + F_W_Mng + Int_Bio + Mng_Vet_D +

Mov_Pur + factor(Phase) + (1 | Farm_n/Hatchery_n)

algorithm: sampling

sample: 8000 (posterior sample size)

priors: see help('prior_summary')

observations: 142

groups: Hatchery_n:Farm_n (95), Farm_n (19)

Estimates:

mean sd 2.5% 97.5%

(Intercept) 0.2 0.9 -1.7 2.2

factor(Cycle)2 -0.1 0.2 -0.6 0.3

factor(Cycle)3 -0.1 0.2 -0.6 0.3

factor(Cycle)4 0.0 0.2 -0.6 0.4

factor(Cycle)5 0.0 0.3 -0.6 0.6

factor(Cycle)6 0.0 0.6 -1.4 1.3

factor(Season)1 0.0 0.2 -0.3 0.5

N_Broilers 0.0 0.0 0.0 0.0

firstweekmort 0.0 0.1 -0.1 0.2

Purpose -0.1 0.3 -0.9 0.5

Ext_Bio -0.5 0.8 -2.6 0.6

Emp_Eq -0.4 0.6 -1.9 0.6

F_W_Mng -0.2 0.9 -2.4 1.6

Int_Bio -0.1 1.0 -2.2 2.2

Mng_Vet_D -0.3 0.5 -1.4 0.4

Mov_Pur 0.1 0.5 -0.9 1.2

factor(Phase)Pre 0.6 0.5 0.0 1.6

b[(Intercept) Hatchery_n:Farm_n:Hatchery_1:Farm_18] 0.1 0.4 -0.5 1.2

b[(Intercept) Hatchery_n:Farm_n:Hatchery_1:Farm_19] 0.1 0.4 -0.6 0.9

b[(Intercept) Hatchery_n:Farm_n:Hatchery_10:Farm_1] 0.0 0.3 -0.7 0.9

b[(Intercept) Hatchery_n:Farm_n:Hatchery_10:Farm_2] 0.0 0.4 -0.7 0.8

b[(Intercept) Hatchery_n:Farm_n:Hatchery_11:Farm_3] 0.0 0.4 -1.0 0.7

b[(Intercept) Hatchery_n:Farm_n:Hatchery_12:Farm_10] -0.1 0.4 -1.0 0.6

b[(Intercept) Hatchery_n:Farm_n:Hatchery_13:Farm_13] 0.2 0.4 -0.4 1.2

b[(Intercept) Hatchery_n:Farm_n:Hatchery_14:Farm_11] 0.2 0.4 -0.4 1.3

b[(Intercept) Hatchery_n:Farm_n:Hatchery_15:Farm_13] -0.1 0.4 -1.0 0.7

b[(Intercept) Hatchery_n:Farm_n:Hatchery_16:Farm_14] 0.0 0.4 -0.9 0.8

b[(Intercept) Hatchery_n:Farm_n:Hatchery_17:Farm_15] 0.0 0.3 -0.6 0.8

b[(Intercept) Hatchery_n:Farm_n:Hatchery_18:Farm_3] -0.1 0.4 -1.0 0.6

b[(Intercept) Hatchery_n:Farm_n:Hatchery_18:Farm_4] -0.1 0.4 -1.0 0.6

b[(Intercept) Hatchery_n:Farm_n:Hatchery_19:Farm_3] 0.0 0.4 -1.0 0.8

b[(Intercept) Hatchery_n:Farm_n:Hatchery_2:Farm_15] 0.0 0.4 -0.7 0.9

b[(Intercept) Hatchery_n:Farm_n:Hatchery_2:Farm_18] -0.1 0.4 -1.0 0.7

b[(Intercept) Hatchery_n:Farm_n:Hatchery_2:Farm_7] -0.1 0.4 -1.2 0.5

b[(Intercept) Hatchery_n:Farm_n:Hatchery_2:Farm_8] 0.0 0.3 -0.6 0.8

b[(Intercept) Hatchery_n:Farm_n:Hatchery_2:Farm_9] -0.1 0.4 -1.1 0.7

b[(Intercept) Hatchery_n:Farm_n:Hatchery_20:Farm_12] 0.0 0.3 -0.7 0.8

b[(Intercept) Hatchery_n:Farm_n:Hatchery_21:Farm_4] 0.0 0.4 -0.9 0.8

b[(Intercept) Hatchery_n:Farm_n:Hatchery_22:Farm_14] -0.1 0.4 -1.2 0.5

b[(Intercept) Hatchery_n:Farm_n:Hatchery_22:Farm_15] 0.0 0.4 -0.7 0.9

b[(Intercept) Hatchery_n:Farm_n:Hatchery_22:Farm_2] 0.1 0.4 -0.6 0.9

b[(Intercept) Hatchery_n:Farm_n:Hatchery_22:Farm_5] 0.1 0.4 -0.5 1.1

b[(Intercept) Hatchery_n:Farm_n:Hatchery_22:Farm_6] -0.1 0.4 -1.1 0.7

b[(Intercept) Hatchery_n:Farm_n:Hatchery_23:Farm_5] -0.1 0.4 -1.0 0.7

b[(Intercept) Hatchery_n:Farm_n:Hatchery_24:Farm_6] 0.0 0.4 -1.0 0.7

b[(Intercept) Hatchery_n:Farm_n:Hatchery_25:Farm_6] -0.1 0.4 -1.2 0.5

b[(Intercept) Hatchery_n:Farm_n:Hatchery_26:Farm_5] -0.1 0.4 -1.1 0.6

b[(Intercept) Hatchery_n:Farm_n:Hatchery_27:Farm_5] -0.1 0.4 -1.0 0.6

b[(Intercept) Hatchery_n:Farm_n:Hatchery_28:Farm_13] -0.1 0.4 -1.1 0.7

b[(Intercept) Hatchery_n:Farm_n:Hatchery_29:Farm_12] 0.0 0.3 -0.8 0.7

b[(Intercept) Hatchery_n:Farm_n:Hatchery_29:Farm_13] 0.1 0.4 -0.5 1.2

b[(Intercept) Hatchery_n:Farm_n:Hatchery_29:Farm_6] 0.2 0.5 -0.3 1.5

b[(Intercept) Hatchery_n:Farm_n:Hatchery_3:Farm_8] 0.1 0.4 -0.5 1.1

b[(Intercept) Hatchery_n:Farm_n:Hatchery_30:Farm_13] 0.0 0.3 -0.7 0.9

b[(Intercept) Hatchery_n:Farm_n:Hatchery_31:Farm_10] 0.1 0.4 -0.5 1.1

b[(Intercept) Hatchery_n:Farm_n:Hatchery_31:Farm_11] 0.1 0.4 -0.5 1.1

b[(Intercept) Hatchery_n:Farm_n:Hatchery_31:Farm_15] 0.0 0.3 -0.7 0.9

b[(Intercept) Hatchery_n:Farm_n:Hatchery_32:Farm_8] 0.0 0.3 -0.6 0.9

b[(Intercept) Hatchery_n:Farm_n:Hatchery_33:Farm_13] -0.1 0.4 -1.1 0.6

b[(Intercept) Hatchery_n:Farm_n:Hatchery_34:Farm_7] -0.1 0.4 -1.0 0.6

b[(Intercept) Hatchery_n:Farm_n:Hatchery_34:Farm_8] 0.0 0.4 -0.7 0.9

b[(Intercept) Hatchery_n:Farm_n:Hatchery_35:Farm_9] -0.1 0.4 -1.0 0.7

b[(Intercept) Hatchery_n:Farm_n:Hatchery_36:Farm_12] 0.1 0.4 -0.6 1.0

b[(Intercept) Hatchery_n:Farm_n:Hatchery_37:Farm_19] -0.1 0.4 -1.1 0.6

b[(Intercept) Hatchery_n:Farm_n:Hatchery_38:Farm_14] 0.0 0.4 -0.9 0.8

b[(Intercept) Hatchery_n:Farm_n:Hatchery_39:Farm_2] 0.1 0.4 -0.6 1.1

b[(Intercept) Hatchery_n:Farm_n:Hatchery_40:Farm_1] 0.1 0.3 -0.6 0.8

b[(Intercept) Hatchery_n:Farm_n:Hatchery_40:Farm_10] -0.1 0.3 -0.8 0.6

b[(Intercept) Hatchery_n:Farm_n:Hatchery_40:Farm_11] -0.2 0.4 -1.3 0.4

b[(Intercept) Hatchery_n:Farm_n:Hatchery_40:Farm_16] 0.1 0.4 -0.5 1.0

b[(Intercept) Hatchery_n:Farm_n:Hatchery_40:Farm_17] -0.1 0.3 -0.9 0.6

b[(Intercept) Hatchery_n:Farm_n:Hatchery_40:Farm_9] -0.1 0.4 -1.0 0.6

b[(Intercept) Hatchery_n:Farm_n:Hatchery_41:Farm_16] 0.0 0.4 -1.0 0.7

b[(Intercept) Hatchery_n:Farm_n:Hatchery_42:Farm_11] -0.1 0.4 -1.1 0.6

b[(Intercept) Hatchery_n:Farm_n:Hatchery_43:Farm_11] 0.0 0.4 -1.0 0.7

b[(Intercept) Hatchery_n:Farm_n:Hatchery_45:Farm_2] -0.1 0.4 -1.0 0.6

b[(Intercept) Hatchery_n:Farm_n:Hatchery_46:Farm_19] 0.2 0.4 -0.4 1.3

b[(Intercept) Hatchery_n:Farm_n:Hatchery_47:Farm_18] 0.0 0.4 -0.9 0.7

b[(Intercept) Hatchery_n:Farm_n:Hatchery_48:Farm_18] 0.0 0.4 -1.0 0.7

b[(Intercept) Hatchery_n:Farm_n:Hatchery_49:Farm_16] 0.0 0.4 -1.0 0.7

b[(Intercept) Hatchery_n:Farm_n:Hatchery_5:Farm_18] -0.1 0.4 -1.0 0.6

b[(Intercept) Hatchery_n:Farm_n:Hatchery_5:Farm_19] 0.0 0.3 -0.8 0.7

b[(Intercept) Hatchery_n:Farm_n:Hatchery_50:Farm_4] -0.1 0.4 -1.0 0.6

b[(Intercept) Hatchery_n:Farm_n:Hatchery_51:Farm_14] 0.0 0.4 -0.9 0.8

b[(Intercept) Hatchery_n:Farm_n:Hatchery_51:Farm_18] 0.0 0.4 -1.0 0.7

b[(Intercept) Hatchery_n:Farm_n:Hatchery_52:Farm_17] -0.1 0.4 -1.0 0.6

b[(Intercept) Hatchery_n:Farm_n:Hatchery_53:Farm_19] -0.1 0.4 -1.2 0.5

b[(Intercept) Hatchery_n:Farm_n:Hatchery_54:Farm_14] 0.0 0.4 -0.9 0.8

b[(Intercept) Hatchery_n:Farm_n:Hatchery_55:Farm_16] 0.0 0.4 -0.9 0.7

b[(Intercept) Hatchery_n:Farm_n:Hatchery_56:Farm_10] 0.0 0.4 -0.9 0.7

b[(Intercept) Hatchery_n:Farm_n:Hatchery_57:Farm_19] 0.1 0.4 -0.5 1.1

b[(Intercept) Hatchery_n:Farm_n:Hatchery_58:Farm_3] 0.0 0.4 -1.0 0.7

b[(Intercept) Hatchery_n:Farm_n:Hatchery_59:Farm_16] 0.1 0.4 -0.5 1.1

b[(Intercept) Hatchery_n:Farm_n:Hatchery_59:Farm_17] 0.0 0.4 -0.8 0.8

b[(Intercept) Hatchery_n:Farm_n:Hatchery_6:Farm_4] 0.0 0.4 -0.9 0.8

b[(Intercept) Hatchery_n:Farm_n:Hatchery_60:Farm_17] 0.0 0.4 -0.8 0.8

b[(Intercept) Hatchery_n:Farm_n:Hatchery_60:Farm_9] 0.2 0.4 -0.4 1.3

b[(Intercept) Hatchery_n:Farm_n:Hatchery_61:Farm_5] 0.2 0.4 -0.4 1.3

b[(Intercept) Hatchery_n:Farm_n:Hatchery_62:Farm_17] 0.1 0.4 -0.6 1.0

b[(Intercept) Hatchery_n:Farm_n:Hatchery_63:Farm_1] 0.0 0.3 -0.7 0.8

b[(Intercept) Hatchery_n:Farm_n:Hatchery_63:Farm_4] 0.0 0.4 -0.9 0.7

b[(Intercept) Hatchery_n:Farm_n:Hatchery_64:Farm_5] -0.1 0.4 -1.0 0.6

b[(Intercept) Hatchery_n:Farm_n:Hatchery_64:Farm_6] -0.1 0.4 -1.0 0.6

b[(Intercept) Hatchery_n:Farm_n:Hatchery_65:Farm_14] 0.0 0.4 -0.9 0.8

b[(Intercept) Hatchery_n:Farm_n:Hatchery_66:Farm_2] -0.1 0.4 -1.1 0.6

b[(Intercept) Hatchery_n:Farm_n:Hatchery_67:Farm_12] -0.1 0.4 -0.9 0.7

b[(Intercept) Hatchery_n:Farm_n:Hatchery_67:Farm_9] -0.1 0.4 -1.0 0.7

b[(Intercept) Hatchery_n:Farm_n:Hatchery_68:Farm_12] 0.1 0.4 -0.5 1.1

b[(Intercept) Hatchery_n:Farm_n:Hatchery_69:Farm_12] 0.1 0.4 -0.6 1.0

b[(Intercept) Hatchery_n:Farm_n:Hatchery_7:Farm_13] -0.1 0.4 -1.0 0.6

b[(Intercept) Hatchery_n:Farm_n:Hatchery_8:Farm_7] 0.1 0.4 -0.5 1.2

b[(Intercept) Hatchery_n:Farm_n:Hatchery_9:Farm_19] 0.0 0.4 -0.6 0.9

b[(Intercept) Farm_n:Farm_1] 0.5 0.6 -0.5 1.7

b[(Intercept) Farm_n:Farm_10] -0.1 0.5 -1.2 1.0

b[(Intercept) Farm_n:Farm_11] 0.0 0.5 -1.1 1.1

b[(Intercept) Farm_n:Farm_12] 0.7 0.6 -0.2 1.9

b[(Intercept) Farm_n:Farm_13] 0.2 0.5 -0.7 1.3

b[(Intercept) Farm_n:Farm_14] -0.9 0.8 -2.9 0.3

b[(Intercept) Farm_n:Farm_15] 0.6 0.5 -0.3 1.8

b[(Intercept) Farm_n:Farm_16] 0.4 0.6 -0.6 1.6

b[(Intercept) Farm_n:Farm_17] -0.2 0.6 -1.3 0.9

b[(Intercept) Farm_n:Farm_18] -0.3 0.5 -1.4 0.7

b[(Intercept) Farm_n:Farm_19] 0.6 0.6 -0.3 1.8

b[(Intercept) Farm_n:Farm_2] 0.1 0.5 -1.0 1.2

b[(Intercept) Farm_n:Farm_3] -0.8 0.8 -2.6 0.4

b[(Intercept) Farm_n:Farm_4] -1.0 0.8 -2.8 0.2

b[(Intercept) Farm_n:Farm_5] 0.1 0.5 -0.9 1.1

b[(Intercept) Farm_n:Farm_6] -0.3 0.6 -1.5 0.7

b[(Intercept) Farm_n:Farm_7] -0.2 0.6 -1.5 0.9

b[(Intercept) Farm_n:Farm_8] 0.8 0.6 -0.1 2.0

b[(Intercept) Farm_n:Farm_9] -0.2 0.6 -1.4 1.0

reciprocal_dispersion 0.9 0.4 0.4 1.9

Sigma[Hatchery_n:Farm_n:(Intercept),(Intercept)] 0.2 0.2 0.0 0.8

Sigma[Farm_n:(Intercept),(Intercept)] 0.7 0.6 0.0 2.2

Fit Diagnostics:

mean sd 2.5% 97.5%

mean_PPD 1.3 0.3 0.8 2.1

The mean_ppd is the sample average posterior predictive distribution of the outcome variable (for details see help('summary.stanreg')).

MCMC diagnostics

mcse Rhat n_eff

(Intercept) 0.0 1.0 5297

factor(Cycle)2 0.0 1.0 8465

factor(Cycle)3 0.0 1.0 7407

factor(Cycle)4 0.0 1.0 10068

factor(Cycle)5 0.0 1.0 10516

factor(Cycle)6 0.0 1.0 11842

factor(Season)1 0.0 1.0 11247

N_Broilers 0.0 1.0 5832

firstweekmort 0.0 1.0 6216

Purpose 0.0 1.0 7982

Ext_Bio 0.0 1.0 3808

Emp_Eq 0.0 1.0 4971

F_W_Mng 0.0 1.0 6956

Int_Bio 0.0 1.0 7819

Mng_Vet_D 0.0 1.0 4550

Mov_Pur 0.0 1.0 7647

factor(Phase)Pre 0.0 1.0 1728

b[(Intercept) Hatchery_n:Farm_n:Hatchery_1:Farm_18] 0.0 1.0 2914

b[(Intercept) Hatchery_n:Farm_n:Hatchery_1:Farm_19] 0.0 1.0 7846

b[(Intercept) Hatchery_n:Farm_n:Hatchery_10:Farm_1] 0.0 1.0 9189

b[(Intercept) Hatchery_n:Farm_n:Hatchery_10:Farm_2] 0.0 1.0 10128

b[(Intercept) Hatchery_n:Farm_n:Hatchery_11:Farm_3] 0.0 1.0 10277

b[(Intercept) Hatchery_n:Farm_n:Hatchery_12:Farm_10] 0.0 1.0 8860

b[(Intercept) Hatchery_n:Farm_n:Hatchery_13:Farm_13] 0.0 1.0 3367

b[(Intercept) Hatchery_n:Farm_n:Hatchery_14:Farm_11] 0.0 1.0 3439

b[(Intercept) Hatchery_n:Farm_n:Hatchery_15:Farm_13] 0.0 1.0 9446

b[(Intercept) Hatchery_n:Farm_n:Hatchery_16:Farm_14] 0.0 1.0 12837

b[(Intercept) Hatchery_n:Farm_n:Hatchery_17:Farm_15] 0.0 1.0 10104

b[(Intercept) Hatchery_n:Farm_n:Hatchery_18:Farm_3] 0.0 1.0 7150

b[(Intercept) Hatchery_n:Farm_n:Hatchery_18:Farm_4] 0.0 1.0 7853

b[(Intercept) Hatchery_n:Farm_n:Hatchery_19:Farm_3] 0.0 1.0 9827

b[(Intercept) Hatchery_n:Farm_n:Hatchery_2:Farm_15] 0.0 1.0 10669

b[(Intercept) Hatchery_n:Farm_n:Hatchery_2:Farm_18] 0.0 1.0 9803

b[(Intercept) Hatchery_n:Farm_n:Hatchery_2:Farm_7] 0.0 1.0 5110

b[(Intercept) Hatchery_n:Farm_n:Hatchery_2:Farm_8] 0.0 1.0 7534

b[(Intercept) Hatchery_n:Farm_n:Hatchery_2:Farm_9] 0.0 1.0 8758

b[(Intercept) Hatchery_n:Farm_n:Hatchery_20:Farm_12] 0.0 1.0 11913

b[(Intercept) Hatchery_n:Farm_n:Hatchery_21:Farm_4] 0.0 1.0 11878

b[(Intercept) Hatchery_n:Farm_n:Hatchery_22:Farm_14] 0.0 1.0 5682

b[(Intercept) Hatchery_n:Farm_n:Hatchery_22:Farm_15] 0.0 1.0 8261

b[(Intercept) Hatchery_n:Farm_n:Hatchery_22:Farm_2] 0.0 1.0 7486

b[(Intercept) Hatchery_n:Farm_n:Hatchery_22:Farm_5] 0.0 1.0 5223

b[(Intercept) Hatchery_n:Farm_n:Hatchery_22:Farm_6] 0.0 1.0 8714

b[(Intercept) Hatchery_n:Farm_n:Hatchery_23:Farm_5] 0.0 1.0 8818

b[(Intercept) Hatchery_n:Farm_n:Hatchery_24:Farm_6] 0.0 1.0 9210

b[(Intercept) Hatchery_n:Farm_n:Hatchery_25:Farm_6] 0.0 1.0 5398

b[(Intercept) Hatchery_n:Farm_n:Hatchery_26:Farm_5] 0.0 1.0 7782

b[(Intercept) Hatchery_n:Farm_n:Hatchery_27:Farm_5] 0.0 1.0 7392

b[(Intercept) Hatchery_n:Farm_n:Hatchery_28:Farm_13] 0.0 1.0 9455

b[(Intercept) Hatchery_n:Farm_n:Hatchery_29:Farm_12] 0.0 1.0 9047

b[(Intercept) Hatchery_n:Farm_n:Hatchery_29:Farm_13] 0.0 1.0 4421

b[(Intercept) Hatchery_n:Farm_n:Hatchery_29:Farm_6] 0.0 1.0 2402

b[(Intercept) Hatchery_n:Farm_n:Hatchery_3:Farm_8] 0.0 1.0 5362

b[(Intercept) Hatchery_n:Farm_n:Hatchery_30:Farm_13] 0.0 1.0 9379

b[(Intercept) Hatchery_n:Farm_n:Hatchery_31:Farm_10] 0.0 1.0 4784

b[(Intercept) Hatchery_n:Farm_n:Hatchery_31:Farm_11] 0.0 1.0 4996

b[(Intercept) Hatchery_n:Farm_n:Hatchery_31:Farm_15] 0.0 1.0 10474

b[(Intercept) Hatchery_n:Farm_n:Hatchery_32:Farm_8] 0.0 1.0 7753

b[(Intercept) Hatchery_n:Farm_n:Hatchery_33:Farm_13] 0.0 1.0 6585

b[(Intercept) Hatchery_n:Farm_n:Hatchery_34:Farm_7] 0.0 1.0 7758

b[(Intercept) Hatchery_n:Farm_n:Hatchery_34:Farm_8] 0.0 1.0 9184

b[(Intercept) Hatchery_n:Farm_n:Hatchery_35:Farm_9] 0.0 1.0 8746

b[(Intercept) Hatchery_n:Farm_n:Hatchery_36:Farm_12] 0.0 1.0 5943

b[(Intercept) Hatchery_n:Farm_n:Hatchery_37:Farm_19] 0.0 1.0 5657

b[(Intercept) Hatchery_n:Farm_n:Hatchery_38:Farm_14] 0.0 1.0 12071

b[(Intercept) Hatchery_n:Farm_n:Hatchery_39:Farm_2] 0.0 1.0 5066

b[(Intercept) Hatchery_n:Farm_n:Hatchery_40:Farm_1] 0.0 1.0 7539

b[(Intercept) Hatchery_n:Farm_n:Hatchery_40:Farm_10] 0.0 1.0 9512

b[(Intercept) Hatchery_n:Farm_n:Hatchery_40:Farm_11] 0.0 1.0 3325

b[(Intercept) Hatchery_n:Farm_n:Hatchery_40:Farm_16] 0.0 1.0 4383

b[(Intercept) Hatchery_n:Farm_n:Hatchery_40:Farm_17] 0.0 1.0 9983

b[(Intercept) Hatchery_n:Farm_n:Hatchery_40:Farm_9] 0.0 1.0 7913

b[(Intercept) Hatchery_n:Farm_n:Hatchery_41:Farm_16] 0.0 1.0 11102

b[(Intercept) Hatchery_n:Farm_n:Hatchery_42:Farm_11] 0.0 1.0 6634

b[(Intercept) Hatchery_n:Farm_n:Hatchery_43:Farm_11] 0.0 1.0 10930

b[(Intercept) Hatchery_n:Farm_n:Hatchery_45:Farm_2] 0.0 1.0 6897

b[(Intercept) Hatchery_n:Farm_n:Hatchery_46:Farm_19] 0.0 1.0 2921

b[(Intercept) Hatchery_n:Farm_n:Hatchery_47:Farm_18] 0.0 1.0 10151

b[(Intercept) Hatchery_n:Farm_n:Hatchery_48:Farm_18] 0.0 1.0 10608

b[(Intercept) Hatchery_n:Farm_n:Hatchery_49:Farm_16] 0.0 1.0 9671

b[(Intercept) Hatchery_n:Farm_n:Hatchery_5:Farm_18] 0.0 1.0 8066

b[(Intercept) Hatchery_n:Farm_n:Hatchery_5:Farm_19] 0.0 1.0 10478

b[(Intercept) Hatchery_n:Farm_n:Hatchery_50:Farm_4] 0.0 1.0 7323

b[(Intercept) Hatchery_n:Farm_n:Hatchery_51:Farm_14] 0.0 1.0 11684

b[(Intercept) Hatchery_n:Farm_n:Hatchery_51:Farm_18] 0.0 1.0 10015

b[(Intercept) Hatchery_n:Farm_n:Hatchery_52:Farm_17] 0.0 1.0 9240

b[(Intercept) Hatchery_n:Farm_n:Hatchery_53:Farm_19] 0.0 1.0 4994

b[(Intercept) Hatchery_n:Farm_n:Hatchery_54:Farm_14] 0.0 1.0 12288

b[(Intercept) Hatchery_n:Farm_n:Hatchery_55:Farm_16] 0.0 1.0 12432

b[(Intercept) Hatchery_n:Farm_n:Hatchery_56:Farm_10] 0.0 1.0 10607

b[(Intercept) Hatchery_n:Farm_n:Hatchery_57:Farm_19] 0.0 1.0 4677

b[(Intercept) Hatchery_n:Farm_n:Hatchery_58:Farm_3] 0.0 1.0 10548

b[(Intercept) Hatchery_n:Farm_n:Hatchery_59:Farm_16] 0.0 1.0 4296

b[(Intercept) Hatchery_n:Farm_n:Hatchery_59:Farm_17] 0.0 1.0 10565

b[(Intercept) Hatchery_n:Farm_n:Hatchery_6:Farm_4] 0.0 1.0 9882

b[(Intercept) Hatchery_n:Farm_n:Hatchery_60:Farm_17] 0.0 1.0 12586

b[(Intercept) Hatchery_n:Farm_n:Hatchery_60:Farm_9] 0.0 1.0 2935

b[(Intercept) Hatchery_n:Farm_n:Hatchery_61:Farm_5] 0.0 1.0 3409

b[(Intercept) Hatchery_n:Farm_n:Hatchery_62:Farm_17] 0.0 1.0 7461

b[(Intercept) Hatchery_n:Farm_n:Hatchery_63:Farm_1] 0.0 1.0 9961

b[(Intercept) Hatchery_n:Farm_n:Hatchery_63:Farm_4] 0.0 1.0 9625

b[(Intercept) Hatchery_n:Farm_n:Hatchery_64:Farm_5] 0.0 1.0 7429

b[(Intercept) Hatchery_n:Farm_n:Hatchery_64:Farm_6] 0.0 1.0 6116

b[(Intercept) Hatchery_n:Farm_n:Hatchery_65:Farm_14] 0.0 1.0 11834

b[(Intercept) Hatchery_n:Farm_n:Hatchery_66:Farm_2] 0.0 1.0 7366

b[(Intercept) Hatchery_n:Farm_n:Hatchery_67:Farm_12] 0.0 1.0 8295

b[(Intercept) Hatchery_n:Farm_n:Hatchery_67:Farm_9] 0.0 1.0 8427

b[(Intercept) Hatchery_n:Farm_n:Hatchery_68:Farm_12] 0.0 1.0 3699

b[(Intercept) Hatchery_n:Farm_n:Hatchery_69:Farm_12] 0.0 1.0 6445

b[(Intercept) Hatchery_n:Farm_n:Hatchery_7:Farm_13] 0.0 1.0 6667

b[(Intercept) Hatchery_n:Farm_n:Hatchery_8:Farm_7] 0.0 1.0 3720

b[(Intercept) Hatchery_n:Farm_n:Hatchery_9:Farm_19] 0.0 1.0 9600

b[(Intercept) Farm_n:Farm_1] 0.0 1.0 4290

b[(Intercept) Farm_n:Farm_10] 0.0 1.0 7752

b[(Intercept) Farm_n:Farm_11] 0.0 1.0 8317

b[(Intercept) Farm_n:Farm_12] 0.0 1.0 2489

b[(Intercept) Farm_n:Farm_13] 0.0 1.0 6690

b[(Intercept) Farm_n:Farm_14] 0.0 1.0 2981

b[(Intercept) Farm_n:Farm_15] 0.0 1.0 3479

b[(Intercept) Farm_n:Farm_16] 0.0 1.0 3507

b[(Intercept) Farm_n:Farm_17] 0.0 1.0 6739

b[(Intercept) Farm_n:Farm_18] 0.0 1.0 6989

b[(Intercept) Farm_n:Farm_19] 0.0 1.0 2891

b[(Intercept) Farm_n:Farm_2] 0.0 1.0 6565

b[(Intercept) Farm_n:Farm_3] 0.0 1.0 3537

b[(Intercept) Farm_n:Farm_4] 0.0 1.0 2874

b[(Intercept) Farm_n:Farm_5] 0.0 1.0 8025

b[(Intercept) Farm_n:Farm_6] 0.0 1.0 6270

b[(Intercept) Farm_n:Farm_7] 0.0 1.0 8390

b[(Intercept) Farm_n:Farm_8] 0.0 1.0 2229

b[(Intercept) Farm_n:Farm_9] 0.0 1.0 8944

reciprocal_dispersion 0.0 1.0 1832

Sigma[Hatchery_n:Farm_n:(Intercept),(Intercept)] 0.0 1.0 1433

Sigma[Farm_n:(Intercept),(Intercept)] 0.0 1.0 1692

mean_PPD 0.0 1.0 7872

log-posterior 0.4 1.0 1153

For each parameter, mcse is Monte Carlo standard error, n_eff is a crude measure of effective sample size, and Rhat is the potential scale reduction factor on split chains (at convergence Rhat=1).

REFERENCE

1. Piironen, J. and A. Vehtari, *Sparsity information and regularization in the horseshoe and other shrinkage priors.* Electronic Journal of Statistics, 2017. **11**(2): p. 5018-5051, 34.
